# Systematic Identification of Immune-Related SnoRNAs: Potential Dual Roles in Tumor Progression and Immunotherapy Response

**DOI:** 10.3390/genes17050581

**Published:** 2026-05-18

**Authors:** Hongling Li, Lihua Zhang, Zhaobin Li, Shuchen Lin

**Affiliations:** 1 Department of Radiation Oncology, Yangtze River Delta Integration Demonstration Zone (QingPu), The Obstetrics & Gynecology Hospital of Fudan University, Shanghai 201700, China; q6022@fckqp.org.cn; 2 Department of Radiation Oncology, Jiahui International Cancer Center, Jiahui Health, Shanghai 200030, China; lihua.zhang@jiahui.com; 3 Department of Radiation Oncology, The Sixth Hospital of Shanghai Jiao Tong University, 600 Yi Shan Rd., Shanghai 200233, China; 4 Department of Radiation Oncology, The Obstetrics & Gynecology Hospital of Fudan University, 128 Shen Yang Rd., Shanghai 200090, China

**Keywords:** pan-cancer, snoRNA, cancer stem cells, immunotherapy response, biomarkers

## Abstract

Background: Immune-related snoRNAs remain largely uncharacterized in cancer. Methods: We comprehensively investigated their functions and clinical relevance through an integrative pan-cancer analysis of The Cancer Genome Atlas (TCGA) datasets. We systematically identified immune-related snoRNAs via partial correlation with immune pathways and GSEA, validated their functions in vitro, and performed molecular subtyping in non-small-cell lung cancer (NSCLC) and head and neck squamous cell carcinoma (HNSC). Results: We established a comprehensive landscape of immune-related snoRNAs associated with core immune pathways, the majority of which were dysregulated across cancers and correlated with tumor immune cell infiltration. Functional screening revealed that numerous immune-related snoRNAs were aberrantly expressed in cancer stem cells; notably, SNORD116-19 potently suppressed breast cancer stemness and metastasis. Using a panel of immune-related snoRNAs, we classified NSCLC into three molecular subtypes with distinct molecular features and immune microenvironments, suggesting divergent immunotherapy response patterns. This classification framework was successfully extrapolated to HNSC. Conclusions: Our findings suggest that immune-related snoRNAs may serve as potential regulators linking tumor progression and immunity and could be explored as candidate biomarkers for molecular subtyping with the potential to inform personalized immunotherapy strategies.

## 1. Introduction

While traditional cancer research has centered on protein-coding genes, the completion of the Human Genome Project revealed a surprising truth: these genes account for only 3~24% of the human genome. The majority are transcribed into non-coding RNAs (ncRNAs), which play pivotal roles in post-transcriptional regulation and are increasingly implicated in tumorigenesis and cancer progression [1]. Among these ncRNAs, small nucleolar RNAs (snoRNAs)—short, conserved molecules composed of between 60 and 300 nucleotides—have remained particularly enigmatic [2]. Despite their abundance and conservation, their precise functional contributions to cancer biology remain poorly understood.

Initially regarded as transcriptional “noise”, small nucleolar RNAs (snoRNAs) have emerged as functionally versatile non-coding RNAs implicated in a wide spectrum of human diseases, especially cancer [3,4]. Beyond the canonical function in rRNA modification and ribosome biogenesis [5,6], snoRNAs exert non-canonical actions including protein interaction [7,8,9,10,11] and production of miRNA-like fragments [12]. These mechanisms enable snoRNAs to regulate diverse cancer-related phenotypes, including proliferation, invasion, metastasis, apoptosis and cancer stemness maintenance [13,14]. Beyond these cell-autonomous roles, mounting evidence suggests that snoRNAs participate in immune regulation—by shaping immune cell differentiation and polarization [15], modulating macrophage M1/M2 switching [16], influencing immune checkpoint expression [17], and even being secreted via extracellular vesicles for intercellular communication [18]. Notably, certain snoRNAs such as SNORA38B have been shown to sensitize tumors to immunotherapy by remodeling the tumor microenvironment [11], and snoRNPs can act as molecular switches in innate immune responses [19].

Despite the annotation of over 2000 snoRNAs in humans [20], the functional relevance of most, particularly within an immuno-oncological context, remains poorly defined. Although previous studies have explored immune-associated snoRNAs in single cancer types [21] or established pan-cancer predictive signatures using a small set of snoRNAs [22], no study has systematically defined cancer-type-specific immune-related snoRNA landscapes. Critically, no study has integrated these cancer-specific immune snoRNAs with cancer stem cell regulation, nor used them to develop cancer-type-specific molecular subtyping systems for stratifying tumors into immune-hot/cold subtypes to guide immunotherapy.

To address this gap, we developed a robust computational pipeline integrating partial correlation analysis (controlling for tumor purity) with gene set enrichment analysis (GSEA) to systematically identify immune-related snoRNAs across 31 cancer types from The Cancer Genome Atlas (TCGA). We further investigated their dysregulation patterns, association with immune cell infiltration, and expression in cancer stem cells. Based on these analyses, we constructed snoRNA-based molecular subtypes for NSCLC and head and neck squamous cell carcinoma (HNSC) and evaluated their ability to stratify patients by predicted immunotherapy response. Our findings suggest that immune-related snoRNAs may serve as potential dual regulators of tumor progression and immune modulation, and that a minimal set of such snoRNAs could inform personalized immunotherapy strategies pending prospective validation.

## 2. Materials and Methods

### 2.1. Data Acquisition and Preprocessing

SnoRNA expression data and corresponding clinical information for 31 cancer types were downloaded from The Cancer Genome Atlas (TCGA) data portal using the R package “TCGAbiolinks” (version 2.38.0). The GSE212550 dataset was obtained from the Gene Expression Omnibus (GEO) for independent external validation. For TCGA snoRNA expression data, we applied log2(x + 1) transformation followed by quantile normalization. For GEO datasets, raw data were normalized using the Robust Multi-Array Average method. All analyses were performed using R version 4.5.1. Seventeen immune-related pathways, comprising 1811 genes, were sourced from the ImmPort (Immunology Database and Analysis Portal) database [23]. All cell lines used in this study were authenticated by the American Type Culture Collection (ATCC) (Manassas, VA, USA) prior to experimental use, verifying that the species origin and genetic characteristics matched the expected identities. In addition, mycoplasma contamination detection was performed on all cell lines before the experiment, and all tested cell lines were confirmed to be free of mycoplasma contamination.

Statistical significance thresholds: Unless otherwise specified, a two-sided *p* < 0.05 was considered statistically significant. For analyses involving multiple comparisons (i.e., partial correlation analysis, gene set enrichment analysis (GSEA), somatic mutation association), the Benjamini–Hochberg false discovery rate (FDR) method was applied to adjust for multiple testing, and adjusted *p*-values (or q-values) are reported accordingly. Detailed thresholds for each specific analysis are provided in the respective sections below.

### 2.2. Estimation of Tumor Microenvironment Scores

The R package “ESTIMATE” (version 1.0.13) was employed to quantitatively characterize the tumor microenvironment (TME) across all cancer types. This generated three key metrics for each tumor sample: the immune score (reflecting immune cell infiltration), the stromal score (indicating stromal content), and tumor purity (the proportion of cancer cells).

### 2.3. Systematic Identification of Immune-Related snoRNAs

**Figure 1:**
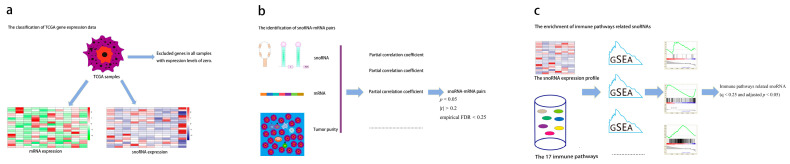
Schematic diagram depicting the strategy for identifying immune-related snoRNAs. (**a**) Preprocessing and classification of TCGA gene expression data. Genes with zero expression across all samples were excluded, and mRNA and snoRNA expression profiles were generated (heat maps: red indicates high expression, green/blue indicates low expression). (**b**) Identification of snoRNA-mRNA pairs. Partial correlation analysis was performed to control for tumor purity, and pairs meeting the criteria of *p* < 0.05, |r| > 0.2 and empirical FDR < 0.25 were retained. (**c**) Enrichment analysis of immune pathway-related snoRNAs. GSEA was performed using the snoRNA expression profile and 17 immune pathway gene sets. Immune pathway-related snoRNAs were defined as those with q < 0.25 and adjusted *p* < 0.05.

Building upon a validated framework for identifying immune-related lncRNAs [24], we developed a robust computational pipeline to define immune-related snoRNAs. First, we excluded genes with expression levels of zero in all samples and classified the remaining genes into snoRNAs and mRNAs. Next we performed partial correlation analysis between each snoRNA and genes from the 17 immune pathways, controlling for tumor purity as a covariate [25] using the “ppcor” R package (version 1.1). For each cancer type, the total number of partial correlation tests was calculated as (number of expressed snoRNAs) × (total number of genes in the 17 immune pathways). Statistical significance was defined as *p* < 0.05. To account for multiple testing in the partial correlation analysis, we applied Benjamini–Hochberg false discovery rate (FDR) correction; however, given that the primary goal of this step was to prioritize candidate associations for subsequent intersection with the GSEA results, we retained the nominal *p* < 0.05 threshold for initial filtering to avoid excluding potentially relevant snoRNAs. The final high-confidence set was determined by intersection with the GSEA results, which provided an additional layer of statistical control. Concurrently, batch GSEA was conducted using clusterProfiler (version 4.18.4) with the following parameters to assess snoRNA–pathway associations: nPerm = 1000, minGSSize = 10, and maxGSSize = 500 to assess snoRNA-pathway associations. For GSEA, samples were dichotomized into high- and low-expression groups for each snoRNA using the median expression value as the cutoff. The association between snoRNA expression and each immune pathway was assessed by comparing these two groups. In GSEA, we adopted a more stringent threshold (q < 0.25 and adjusted *p* < 0.05) because GSEA aggregates information across gene sets, reducing the number of independent tests and enhancing robustness against false positives. The use of the q-value (FDR) here is standard for pathway-level analyses. The final high-confidence set of immune-related snoRNAs was defined as the intersection of snoRNAs that satisfied the following: (i) partial correlation with at least one immune pathway gene at nominal *p* < 0.05, |r| > 0.2 and empirical FDR < 0.25 (from permutation test), and (ii) GSEA enrichment for any immune pathway at q < 0.25 and adjusted *p* < 0.05. This pipeline was applied uniformly across the 31 cancer types (Figure 1).

### 2.4. Permutation Test and Sensitivity Analyses for Partial Correlation

To statistically validate the partial correlation associations, we performed A permutation test for each candidate snoRNA–immune gene pair (i.e., pairs with nominal partial correlation *p* < 0.05). For each pair, the snoRNA expression vector was randomly permuted 1000 times, while immune gene expression and tumor purity (covariate) remained fixed. For each permuted Dataset, we recalculated the partial correlation coefficient using A shrinkage estimator (pcor.shrink from the corpcor R package (version 1.6.10)). The empirical *p*-value was defined as (number of permutations with absolute partial correlation ≥ the observed absolute value + 1)/(1000 + 1). Empirical false discovery rates (empirical fdrs) were then calculated using the Benjamini–Hochberg method across all tested pairs. A snoRNA–immune gene pair was considered robust if it satisfied (i) a nominal partial correlation *p* < 0.05 and (ii) an empirical fdr < 0.25. Note that this permutation test was applied only to the partial correlation analysis and not to the Gsea, for which standard implementation already includes gene set permutations.

To evaluate the robustness of our identification pipeline, we performed sensitivity analyses by varying three key parameters: the absolute partial correlation coefficient threshold (denoted as |r|), the false discovery rate (FDR) threshold, and the *p*-value threshold. |r| thresholds were as follows: 0.00, 0.02, 0.05, 0.08, 0.10, 0.12, 0.15, 0.20, 0.25, and 0.30. FDR thresholds were as follows: 0.01, 0.05, 0.10, 0.15, 0.20, 0.25, 0.30, 0.40, and 0.50. *p*-value thresholds were as follows: 0.001, 0.005, 0.01, 0.02, 0.05, 0.10, 0.15, 0.20, and 0.30. For each threshold series, we varied one parameter while holding the other two at their baseline values. For every combination, we calculated three metrics: (i) the number of robust snoRNA–immune pathway pairs satisfying the given thresholds, (ii) the Jaccard similarity between the resulting set and the baseline set, and (iii) the retention rate of the top 100 most significant pairs (based on original *p*-values) under the tested thresholds.

### 2.5. Analysis of snoRNA Expression Perturbation in Cancer

Differential expression analysis of snoRNAs was performed on 15 cancer types in the TCGA with sufficient normal samples (*n* ≥ 5). For snoRNAs expressed in >70% of samples, we performed differential expression using Student’s *t*-test. For snoRNAs with a high zero-expression rate (≥30% of samples), we employed an “On/Off” status analysis: a snoRNA was considered “expressed” (On) if its normalized expression level log2 (transcripts per million (TPM) + 1) was >0, and “not expressed” (Off) otherwise. Fisher’s exact test was then used to compare the proportion of “On” samples between tumor and normal groups. For all differential expression analyses, *p*-values were adjusted using the Benjamini–Hochberg method, and an FDR < 0.05 was considered significant.

### 2.6. Inference of snoRNA Expression in Immune Cell Types

Leveraging the RESPECTEx deconvolution framework [25], we inferred immune cell-specific expression profiles of snoRNAs from bulk RNA-Seq data. This method operates on the principle that the observed expression of each gene in a tumor sample represents a mixture of contributions from multiple cell types, weighted by their relative abundance. Specifically, the bulk expression *B_g_*_,*s*_ of gene *g* in sample *s* can be modeled as follows:

$$B_{g,s}=[\beta_{g,1},\beta_{g,2},\beta_{g,3}\cdots ,\beta_{g,c}]\left(\begin{array}{c} x_{1,s}\\ x_{2,s}\\ x_{3,s}\\ \cdots \\ x_{c,s} \end{array}\right)$$

where *β_g_*_,*c*_ denotes the expression level of gene *g* in cell type *c*, and *Χ_C_*_,*s*_ represents the proportion of cell type cc in sample *s*. Tumor purity was used as the estimate for tumor cell proportion, while immune cell fractions were inferred using Tumor Immune Estimation Resource (TIMER) (R package version 2.0, default settings) and Cell-type Identification By Estimating Relative Subsets Of RNA Transcripts (CIBERSORT) (1000 permutations, LM22 signature matrix). We then applied the Wilcoxon rank-sum test to systematically compare the average expression levels of immune-related snoRNAs against all other snoRNAs across the reconstructed cell type-specific expression profiles.

### 2.7. Association Analysis with Immune Cell Infiltration

Spearman correlation coefficients (SCCs) were calculated between each snoRNA and the abundance of six major immune cell types (B cells, CD8+ T cells, CD4+ T cells, macrophages, neutrophils, and dendritic cells) estimated by TIMER [26]. The analysis was performed using the cor.test function in R with method = “spearman”, exact = FALSE. No additional multiple testing correction was applied at this stage, as each snoRNA–immune cell pair was tested independently for exploratory purposes. snoRNAs with a correlation coefficient ≥ 0.3 and *p* < 0.05 were deemed infiltration-related. Fisher’s exact test assessed the enrichment of these snoRNAs among the previously identified immune-related snoRNAs. To ensure robustness, this analysis was repeated using immune proportions estimated by CIBERSORT [27], with consistency evaluated via hypergeometric testing.

### 2.8. Molecular Subtyping Based on Immune-Related snoRNAs

We applied our pipeline to a pan-NSCLC cohort (integrating lung adenocarcinoma (LUAD) and lung squamous cell carcinoma (LUSC)) to identify immune-related snoRNAs. The immune-related snoRNAs were defined as the intersection of snoRNAs satisfying (i) partial correlation with any immune pathway gene at nominal *p* < 0.05, |r| > 0.2, and an empirical FDR < 0.25 (from permutation test), and (ii) GSEA enrichment for any immune pathway at q < 0.25 and adjusted *p* < 0.05, as described in Section 2.3. To focus on snoRNAs that broadly shape the tumor immune microenvironment, we further required that they be significantly correlated with any immune cell type as estimated by TIMER (|correlation coefficient| ≥ 0.3, *p* < 0.05). The intersection of these criteria yielded 46 high-confidence snoRNAs for NSCLC subtyping. Expression data were log10-transformed. Then, the snoRNAs with the highest median absolute deviation (MAD) were selected for further analysis. For each selected snoRNA, expression values were centered by subtracting the median (no scaling by MAD). Unsupervised consensus clustering was performed using ConsensusClusterPlus (version 1.74.0) with the following parameters: 1000 iterations, 80% resampling (pItem = 0.8), Spearman distance, hierarchical clustering algorithm (clusterAlg = “hc”), and k ranging from 2 to 6. Consensus clustering, cumulative distribution function (CDF) and delta area plots consistently identified k = 3 as the optimal and most robust number of subtypes. To evaluate robustness against overfitting, we conducted a repeated Monte Carlo cross-validation (80/20 train–test split, 100 independent iterations, fixed random seed). In each iteration, the same consensus clustering procedure (46 snoRNAs, Spearman distance, hierarchical clustering, k = 3) was applied to the training set to derive cluster centroids. Validation samples were assigned to the nearest centroid, and the predicted labels were compared with the full-dataset reference labels using the adjusted Rand index (ARI). The mean ARI and its 95% confidence interval were calculated across 100 iterations. Analyses were performed using R packages mclust (version 6.1.2).

The resulting subtypes were characterized using multi-omics data, including molecular subtypes, pathway activities, stemness indices, and somatic mutation profiles. Of them, molecular subtypes and pathway activities were curated from Chen et al. [28], stemness indices were obtained from Malta et al. [29], and gene set enrichment was assessed via GSVA. Somatic mutations were analyzed with the R package maftools (version 2.26.0), with significance thresholds set at |logFC| > 0.1 and FDR-adjusted *p* < 0.05 (Benjamini–Hochberg).

The same workflow was applied to HNSC for validation, yielding 39 high-confidence snoRNAs. The immunotherapeutic relevance of the resulting subtypes was evaluated using the TIDE platform [30] to estimate immune escape potential and predict response to immune checkpoint blockade. The GSE212550 clinical trial dataset [31] was used to validate clinical benefit from immunotherapy.


**Step**

**Criterion**

**Quantitative Threshold**

**Cancer Type**
1Expression filteringRemove snoRNAs with zero expression in all samplesNSCLC, HNSC2Identification of immune-related snoRNAs (partial correlation)|r| > 0.2, nominal *p* < 0.05, empirical FDR < 0.25NSCLC, HNSC3Identification of immune-related snoRNAs (GSEA)q < 0.25, adjusted *p* < 0.05NSCLC, HNSC4Final immune-related snoRNA setIntersection of step 2 and step 3NSCLC, HNSC5Correlation with immune infiltra-tion (TIMER)|Spearman r| ≥ 0.3, *p* < 0.05 for any immune cell typesNSCLC, HNSC6Final subtyping snoRNA setIntersection of step 4 and step 5NSCLC: 46 genes; HNSC: 39 genes

### 2.9. Assessment of Immune Landscape and Therapeutic Potential

To comprehensively characterize the tumor immune landscape, we evaluated a panel of immune-related scores. We first assessed the overall immune activity using a collection of 143 immune expression signatures derived from a published study [32]. Additionally, based on established methodologies [33,34], we calculated two specific scores: the MHC score, defined as the mean log-transformed and median-centered expression of a core set of MHC-I genes (*HLA-A*, *HLA-B*, *HLA-C*, *TAP1*, *TAP2*, *NLRC5*, *PSMB9*, *PSMB8*, and *B2M*), and the Cytolytic Activity (CYT) score, computed as the mean expression of *GZMA* and *PRF1* following the same normalization. Finally, to predict response to immune checkpoint inhibitors (ICIs), we utilized the immunophenoscore (IPS). The immune phenotype score (IPS) integrates Z-scores of immune-related genes, where higher scores indicate a better response tendency to immune checkpoint inhibitors (ICIs) [35]. IPS values were obtained from The Cancer Immunome Atlas (TCIA, https://tcia.at/home, accessed 15 March 2023), and pre-calculated scores corresponding to TCGA-LUAD and TCGA-LUSC samples were adopted in this study. Additionally, TIDE scores were calculated using the online TIDE platform (http://tide.dfci.harvard.edu/, accessed 15 March 2023) with default parameters (gene expression data log2-transformed, no additional normalization).

### 2.10. Experimental Validation of Immune-Related snoRNA Function

First, using breast cancer as a model, we investigated the prevalence of immune-related snoRNAs dysregulated in breast cancer stem cells (CSCs). We enriched CSCs via mammosphere culture and subsequently quantified the differential expression of snoRNAs between CSCs and adherent tumor cells using RT-qPCR.

Second, based on the above initial screening, SNORD116-19 was chosen for in-depth functional analysis. We generated stable SNORD116-19-overexpressing clones in triple-negative breast cancer (TNBC) cell lines to assess its impact on malignant phenotypes.

Cellular phenotyping: The impact on malignant behaviors was evaluated through a series of functional assays. Cell proliferation was measured by using a colony formation assay; migratory and invasive capabilities were assessed using Transwell chambers (8-μm pore size, 6.5-mm diameter; Corning Costar, 3422) with or without Matrigel coating, respectively.

Stemness assessment: CSC self-renewal potential was specifically quantified using the mammosphere formation assay. The ALDH-positive cell population, a marker of stemness, was analyzed by flow cytometry using the Aldefluor kit (STEMCELL Technologies, Vancouver, BC, Canada).

Mechanistic investigation: To uncover the underlying molecular pathways, global transcriptomic changes upon SNORD116-19 overexpression were profiled by RNA sequencing (RNA-seq). Differential expression and pathway enrichment analyses were performed.

Detailed experimental procedures are described in the Supplementary Materials and Methods.

## 3. Results

### 3.1. Immune-Related snoRNAs Are Prevalent and Linked to Core Immunological Pathways

**Figure 2:**
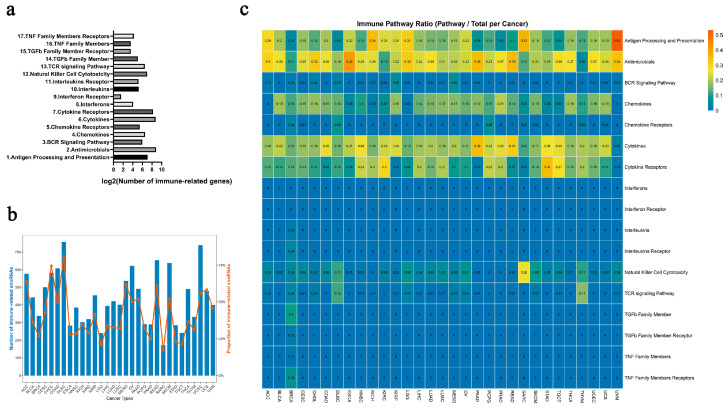
Prevalence of immune-related snoRNAs across 31 cancer types and their association with core immunological pathways. (**a**) The number of genes in the 17 immunologically significant gene sets. (**b**) The number (blue bars, left y-axis) and proportion (orange line, right y-axis) of immune-related snoRNAs identified in each cancer type. (**c**) Heatmap illustrating the percentage of immune-related snoRNAs associated with 17 immunological pathways across 31 cancer types. Color intensity corresponds to the pathway ratio (pathway/total per cancer), ranging from 0 (dark blue) to 0.5 (dark orange), as shown in the color bar.

Employing a systematic multi-omics approach, we identified a comprehensive set of immune-related snoRNAs across 31 cancer types (sample details in Table S1). We hypothesized that these snoRNAs play functional roles not only in tumor development but also in shaping the immune microenvironment. To test this, we analyzed their association with 17 well-defined immune pathways from the ImmPort database [36], which encompassed between 3 and 516 genes per pathway (Figure 2a, Table S2). Strikingly, between 17.0% and 81.1% of expressed snoRNAs per cancer type were classified as immune-related, reflecting their widespread involvement in immuno-regulatory processes (Figure 2b). Notably, these snoRNAs were most frequently linked to fundamental immunological mechanisms such as “antigen processing and presentation”, “antimicrobials”, “chemokines”, “cytokines”, and “cytokine receptors” (Figure 2c)—pathways increasingly recognized as actionable targets for immunotherapy [37,38,39]. These results suggest that immune-related snoRNAs could be promising molecular candidates, potentially offering new avenues for mechanistic investigation and the development of personalized immunotherapeutic strategies.

Permutation testing and sensitivity analysis corroborated the robustness of our identification pipeline. Permutation tests (1000 shuffles) for each candidate snoRNA–immune gene pair demonstrated that the observed partial correlations were highly significant, with the majority of pairs achieving an empirical FDR < 0.25. Sensitivity analysis in breast cancer (BRCA) further showed that varying the partial correlation threshold from 0 to 0.25 did not alter the number of immune-related snoRNA–pathway pairs (9976 pairs, Jaccard = 1.0, top 100 retention = 0.96); only at |r| > 0.30 did the pair count drop to 2777. Changing the FDR threshold (0.01–0.5) or the nominal *p*-value threshold (0.001–0.3) had negligible or no impact on any metric (all Jaccard ≥ 0.999, top 100 retention ≥ 0.92) (Figure S1, Table S3). Similar stability was observed across all other cancer types. Based on these results, we selected the following thresholds for final identification: partial correlation *p* < 0.05, empirical FDR < 0.25, and |r| > 0.2.

### 3.2. Immune-Related snoRNAs Drive Oncogenesis and Modulate Cancer Stemness

**Figure 3:**
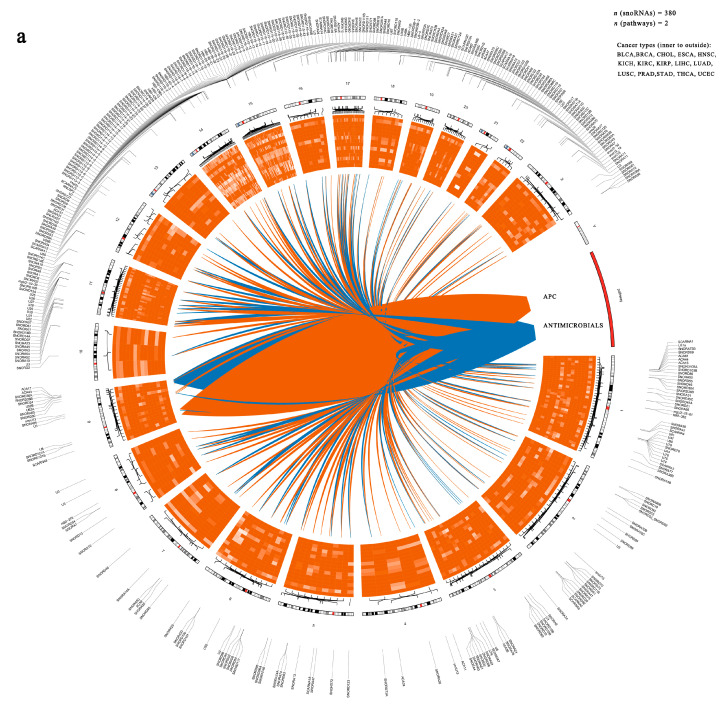

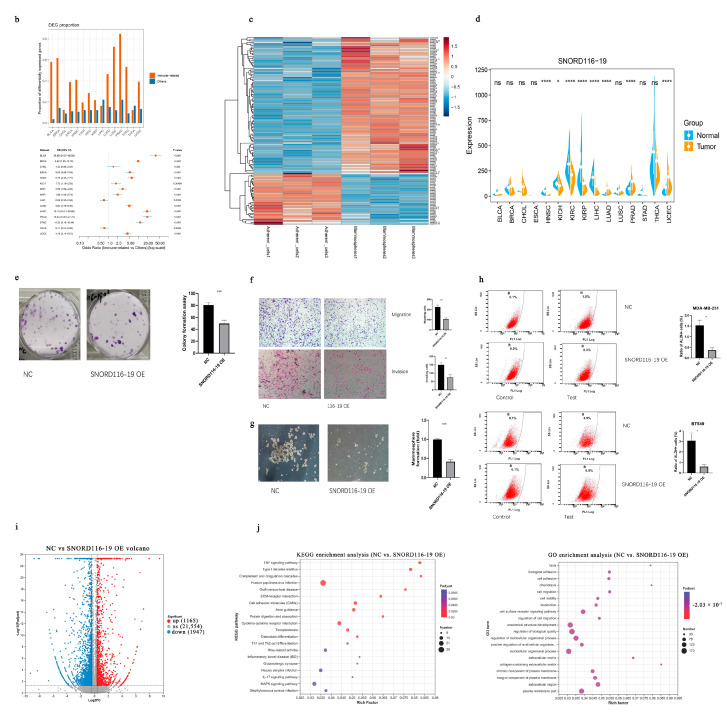
The important role of immune-related snoRNAs in cancer. (**a**) Circos plot displaying the top 500 snoRNA–immune pathway pairs across cancer types. The inner heatmap shows expression patterns of immune-related snoRNAs (orange–red: upregulation; dark blue: downregulation). Ribbons connect snoRNAs to the two main pathways (orange: APC; blue: Antimicrobials). The outer circle indicates the genomic locations of the corresponding snoRNAs. (**b**) Top: Bar plot showing the proportion of aberrant expressed immune-related snoRNAs (orange) and other snoRNAs (blue) across cancer types. Bottom: Forest plot summarizing odds ratios (ORs) and 95% confidence intervals (CIs) for immune-related snoRNA enrichment. Except for CHOL, all *p* values are < 0.05 (two-sided Fisher’s exact test). Error bars represent 95% CIs. (**c**) Heatmap of snoRNA expression differences between mammosphere cells and adherent MDA-MB-231 cells. Color intensity represents normalized expression (orange: high; blue: low). (**d**) Split violin plots illustrating SNORD116-19 expression in tumor vs. adjacent normal tissues across cancer types. (**e**) Colony formation assay of stably transfected MDA-MB-231 cells over 14 days. Representative images and quantitative analysis are shown. (**f**) Transwell migration and invasion assays of stably transfected MDA-MB-231 cells (scale bar: 100 μm). Representative images and quantitative data are presented. (**g**) Mammosphere formation assay results for SNORD116-19 overexpression (OE) in MDA-MB-231 cells. Data represent the mean ± S.D. from three independent experiments. (**h**) Aldefluor assay for the ALDH^+^ cell population in NC and SNORD116-19 OE cells. DEAB was used as the negative control. Flow cytometry plots and quantitative analysis are shown. (**i**) Volcano plot of differentially expressed genes from RNA-seq of NC vs. SNORD116-19 OE MDA-MB-231 cells (red: upregulated, blue: downregulated, gray: not significant). (**j**) Top 20 enriched Kyoto Encyclopedia of Genes and Genomes (KEGG) pathways and Gene Ontology (GO) terms for genes differentially expressed between NC and SNORD116-19 OE cells. Statistical significance is indicated as: * *p* < 0.05, ** *p* < 0.01, *** *p* < 0.001, **** *p* < 0.0001; ns, not significant.

To uncover the functional significance of immune-related snoRNAs in cancer, we began by constructing a regulatory network based on the top 500 snoRNA–pathway associations across multiple cancer types (Figure 3a and Table S4). This network comprised 380 immune-related snoRNAs, most of which were upregulated in tumors (Figure 3a).

We then assessed the broader relevance of immune-related snoRNAs in tumorigenesis, we analyzed their expression dysregulation across 15 cancer types. In most cancers, immune-related snoRNAs were significantly more likely to be dysregulated than other snoRNA populations. However, CHOL showed no significant difference, while LIHC and THCA exhibited an opposite trend, with immune-related snoRNAs being less frequently dysregulated (Figure 3b). This bias was especially strong in immunotherapy-sensitive cancers such as BLCA, BRCA, PRAD, and LUSC. For instance, in BRCA, 62% of immune-related snoRNAs showed aberrant expression versus only 14% for other snoRNAs. These results suggest that our approach may help identify functionally relevant snoRNAs with diagnostic and therapeutic potential.

Given the established critical role of CSCs in tumor initiation, progression, metastasis, and therapy resistance [40], we sought to determine whether immune-related snoRNAs contribute to CSC regulation. Using mammosphere culture to enrich breast CSCs [41], we performed gene expression profiling and found that approximately one-third of immune-related snoRNAs (104 out of 380) were differentially expressed in CSCs relative to bulk tumor cells, with 27 downregulated and 77 upregulated (Figure 3c). These findings suggest that immune-related snoRNAs may function as important regulators of CSC maintenance.

Then, we selected SNORD116-19 for functional investigation based on the following considerations: (i) it is a previously uncharacterized snoRNA with no reported function in cancer; (ii) its expression is dysregulated in multiple cancer types (Figure 3d), suggesting broad clinical relevance; and (iii) it represents a candidate with potential tumor-suppressive activity, as its expression trended lower in several tumors, though not significantly in BRCA. We therefore selected it as a proof-of-concept molecule to test whether an immune-related snoRNA could functionally impact cancer stemness and malignancy. We acknowledge that these experiments validate the tumor-suppressive effect of a single snoRNA in one cellular context (TNBC) and do not directly address the broader immune regulatory framework proposed in our computational analyses. Strikingly, SNORD116-19 inhibited cell proliferation (Figure 3e), migration, and invasion (Figure 3f). Furthermore, its overexpression strongly suppressed mammosphere formation (Figure 3g) and reduced the proportion of ALDH+ cells (Figure 3h), indicating impaired CSC activity. To explore the underlying mechanism, we performed RNA-seq in SNORD116-19-overexpressing MDA-MB-231 cells and identified 1947 downregulated and 1165 upregulated genes (Figure 3i). These genes were enriched in metastasis-related processes—including cell adhesion, migration, and extracellular matrix organization—as revealed by Gene Ontology (GO) and Kyoto Encyclopedia of Genes and Genomes (KEGG) analyses (Figure 3j). Key pathways such as tumor necrosis factor (TNF) signaling, interleukin-17 (IL-17) signaling, and extracellular matrix (ECM)–receptor interaction were prominently affected. These RNA-seq findings suggest potential mechanisms, but they are exploratory and require independent validation by quantitative polymerase chain reaction (qPCR) or protein-level assays (i.e., Western blot) in future studies. Thus, while SNORD116-19 serves as a proof-of-concept demonstration that an immune-related snoRNA can functionally impact cancer stemness and malignancy, the generalizability of these findings to other immune-related snoRNAs and cancer types requires further experimental validation.

### 3.3. Immune-Related snoRNAs Are Enriched in Immune Cells and Correlate with Immune Infiltration

**Figure 4:**
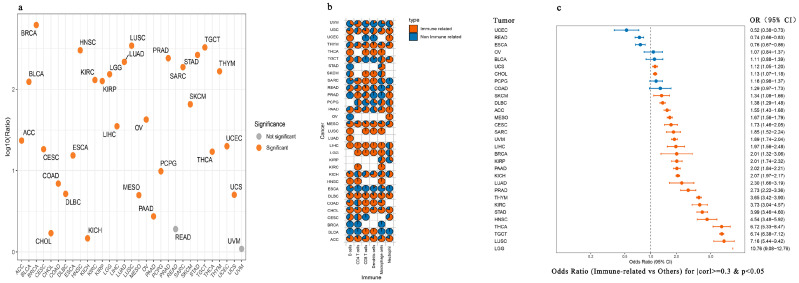
Immune-related snoRNAs are associated with immune cell infiltration. (**a**) Increased expression of immune-related snoRNAs in immune cell populations. The y-axis displays the log10(ratio) between the average expression of immune-related snoRNAs and others in immune cells. Orange-red dots represent cancer types with a significant difference (Wilcoxon rank-sum test, *p* < 0.05), while gray dots indicate non-significant results (*p* ≥ 0.05). (**b**) Distribution of immune-related (orange) and non-immune-related (blue) snoRNAs among immune cell infiltration-associated snoRNAs across cancer types and immune cell populations. (**c**) Immune-related snoRNAs are more likely to be enriched for snoRNAs associated with immune cell infiltration. The figures are presented as odds ratios (ORs) with 95% confidence intervals. Orange-red dots indicate significant enrichment (OR > 1, *p* < 0.05), while blue dots represent non-significant results. The vertical dashed line at OR = 1 indicates no enrichment.

Building on the identification of immune-related snoRNAs, we hypothesized that these molecules might be highly expressed within immune cells and correlate with immune cell infiltration in the tumor microenvironment. To test this, we leveraged TCGA bulk RNA-seq data and the RESPECTEx model [24] to define the snoRNA expression profiles of 22 immune cell types compared with tumor cells. Consistent with our hypothesis, immune-related snoRNAs displayed significantly higher expression in immune cells across most cancer types (Figure 4a).

We next probed the relationship between these snoRNAs and tumor immunity by analyzing correlations with the infiltration levels of six key immune cell types—B cells, CD4+ T cells, CD8+ T cells, macrophages, neutrophils, and dendritic cells—using TIMER. Strikingly, a substantial proportion of snoRNAs significantly associated with immune cell infiltration were indeed immune-related (*p* < 0.05, Figure 4b). This trend was particularly evident in cancers such as diffuse large B-cell lymphoma (DLBC), where over 90% of infiltration-associated snoRNAs were classified as immune-related (Figure 4b). Statistical validation using Fisher’s exact test further confirmed that immune-related snoRNAs were significantly overrepresented among those linked to immune infiltration in most cancer types (Figure 4c).

To ensure the robustness of our findings, we replicated the analysis using the CIBERSORT algorithm. The results showed a high degree of concordance with those obtained from TIMER, reinforcing the reliability of the association (Table 1).

In summary, our multi-faceted analysis indicates that immune-related snoRNAs may be enriched in immune cells and show correlations with immune cell infiltration in tumors, suggesting their possible relevance in the regulation of anti-tumor immunity.

### 3.4. Molecular Subtyping of Nsclc Using Immune-Related snoRNAs

Given the crucial regulatory role of immune-related snoRNAs in tumor initiation and progression, we proposed that they could serve as novel biomarkers for molecular cancer subtyping. Lung cancer, the leading cause of cancer-related deaths globally, stands to benefit greatly from molecular classification strategies that help tailor treatments and improve patient survival. With this goal, we sought to develop a snoRNA-based subtyping system for non-small-cell lung cancer (NSCLC).

We first compiled a set of immune-related snoRNAs. Using the TIMER database, we then identified snoRNAs whose expression was significantly correlated with immune cell infiltration, applying a threshold of |correlation coefficient| ≥ 0.3 and *p* < 0.05 for any immune cell type in NSCLC. By taking the intersection of the immune-related snoRNAs and those meeting the TIMER significance criteria, we selected 46 key snoRNAs that are involved in immune response and tumor microenvironment regulation. Using these snoRNAs, we unambiguously stratified NSCLC into three stable and biologically distinct molecular subtypes (k = 3). Figure S2 shows that CDF curve, and delta area plot both confirmed that k = 3 was the optimal number of clusters. To evaluate the stability of the snoRNA-based subtyping framework, we performed 10-fold cross-validation. The mean adjusted Rand index (ARI) between the cluster assignments in training and testing folds was 0.501 ± 0.081, indicating moderate agreement beyond random chance (Figure S3). This result suggests a moderate risk of overfitting, likely attributable to the limited sample size and high biological heterogeneity of NSCLC.

**Figure 5:**
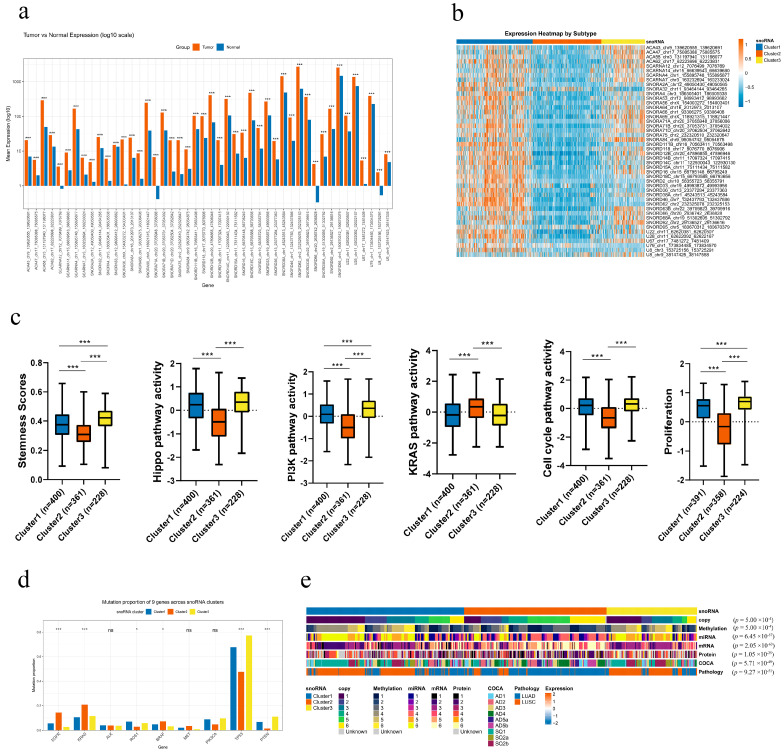
The molecular features of the three NSCLC subtypes. (**a**) Differential expression of the 46 subtyping-related snoRNAs between tumor (orange bars) and adjacent non-cancerous tissues (blue bars), shown on a log10-transformed scale. Statistical significance is indicated as follows: *** *p* < 0.001. (**b**) Expression heatmap of the 46 subtyping-related snoRNAs across the three NSCLC subtypes. Color intensity represents normalized expression levels (orange: high expression, blue: low expression). The top bar indicates the three subtypes (Cluster 1: blue, Cluster 2: orange, Cluster 3: yellow). (**c**) Box plots comparing key biological characteristics across the three subtypes, including stemness scores and the activity of the Hippo, PI3K, KRAS, cell cycle, and proliferation pathways. Boxes represent the interquartile range (IQR), with the median line inside; whiskers extend to 1.5 × IQR. Cluster 1 is shown in blue, Cluster 2 in orange, and Cluster 3 in yellow. Statistical significance is indicated as: *** *p* < 0.001. (**d**) Proportion of patients with mutations in common NSCLC driver genes across the three subtypes (Cluster 1: blue, Cluster 2: orange, Cluster 3: yellow). The corresponding *p*-values from the chi-square test are shown above each gene bar plot. Statistical significance is indicated as follows: * *p* < 0.05, *** *p* < 0.001; ns, not significant. (**e**) Concordance between the three snoRNA-based subtypes and previously published NSCLC molecular subtypes. Each row represents a different classification system, and each column represents a sample colored by its assigned subtype. The association was assessed using the chi-square test.

Expression analysis revealed that all 46 snoRNAs were significantly upregulated in tumor tissues compared with adjacent normal tissues (unpaired *t*-test, all *p* < 0.001; Figure 5a). The resulting heatmap demonstrates that most of these snoRNAs were highly expressed in Clusters 1 and 3 (Figure 5b). This new classification approach could potentially offer a meaningful framework for personalized therapy in lung cancer, though this remains to be validated.

### 3.5. Distinct Molecular Phenotypes of the snoRNA-Based Subtypes in Nsclc

Our analysis revealed distinct, clinically relevant molecular profiles for the three snoRNA-based subtypes. Clusters 1 and 3 were characterized by the most aggressive phenotype, demonstrating the highest enrichment scores for the Hippo and PI3K signaling pathways, cellular stemness, cell cycle, and proliferation. In contrast, Cluster 2 exhibited the lowest activity in these pathways and features, but showed the highest enrichment for the KRAS signaling pathway (Figure 5c).

To further dissect these differences, we performed Gene Set Variation Analysis (GSVA). This confirmed the proliferative signature of Clusters 1 and 3, showing elevated activity in cell cycle, MYC targets, DNA replication, DNA repair, G2M checkpoint and oxidative phosphorylation pathways. Conversely, Clusters 1 and 2, especially Cluster 2, were markedly enriched for a variety of immune-related pathways (Figure S4), suggesting a more immunologically active tumor microenvironment.

We next investigated the mutational landscape across the subtypes. Clusters 1 and 3 were associated with a higher mutation frequency in *ROS1, TP53,* and *PTEN*. Meanwhile, Cluster 2 showed a greater prevalence of mutations in *EGFR*, *KRAS*, and *BRAF*. No significant differences were observed in the mutation rates of *ALK*, *PIK3CA*, or *MET* (Figure 5d). This distinct mutational patterning indicates that snoRNA-based subtyping could provide valuable guidance for selecting targeted therapies.

We next compared the three snoRNA-based subtypes with previously established multi-omics classifications and clinicopathological features in NSCLC, including copy number, methylation, miRNA, mRNA, protein, ConsensusOmics Classification (COCA) molecular subtype, and pathological type. All comparisons were highly consistent and significant (all *p* < 0.001). Cluster 1 was strongly dominated by squamous-like features, with significant enrichment for SQ1, SQ2a, and SQ2b in the COCA classification and a high proportion of LUSC in pathological diagnosis. Meanwhile, Cluster 1 showed a characteristic distribution pattern in copy number, methylation, miRNA, mRNA, and protein subtypes. Cluster 2 was predominantly associated with adenocarcinoma (LUAD) and was significantly enriched in AD4, AD5a, and AD5b subtypes in the COCA classification, representing a classical LUAD-like molecular phenotype. Cluster 3 exhibited a mixed but distinctive profile, with a high proportion of LUSC and enrichment for AD1, AD2, AD3 and SQ1/SQ2b subtypes, suggesting an intermediate or invasive phenotype combining adenocarcinoma and squamous markers (Figure 5e and Table S5). Collectively, these results demonstrate that our snoRNA-based subtyping faithfully recaptures major multi-omics and pathological classifications of NSCLC, supporting its robustness and clinical relevance as a novel molecular stratification tool.

### 3.6. Immune-Related snoRNA-Based Subtyping Suggests Differential Immunotherapy Response Potential in Nsclc

**Figure 6:**
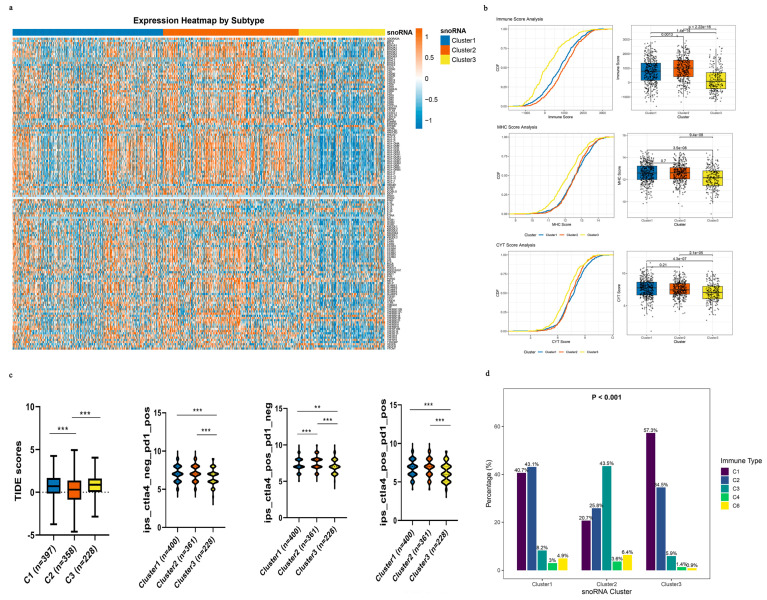
Immune-related snoRNAs could identify different immunotherapy-responsive subtypes in NSCLC. (**a**) Expression heatmap of 143 immune-related snoRNAs across the three subtypes in TCGA samples. Color intensity represents normalized snoRNA expression levels (orange: high expression, blue: low expression). The top bar indicates the three subtypes (Cluster 1: blue, Cluster 2: orange, Cluster 3: yellow). (**b**) Cumulative distribution curves (left panels) and box plots (right panels) comparing immune, CYT, and MHC scores across the three subtypes. In the box plots, Cluster 1 is shown in blue, Cluster 2 in orange, and Cluster 3 in yellow. (**c**) TIDE and IPS immunotherapy response scores across the three subtypes. TIDE scores are shown in the leftmost panel; IPS scores for anti-PD-1 monotherapy, anti-CTLA-4 monotherapy, and combination therapy are shown in the subsequent panels. Boxes represent the interquartile range, with the median line inside; whiskers extend to 1.5 × IQR. Statistical significance is indicated as: ** *p* < 0.01, *** *p* < 0.001. Cluster colors are consistent with those in panel (**a**). (**d**) Distribution of previously defined immune subtypes across the three snoRNA-based subtypes. The proportion of each immune subtype (C1–C6) within each snoRNA cluster is shown, with statistical significance assessed by the chi-square test (*p* < 0.001).

To delineate the immune landscape of the three snoRNA-defined NSCLC subtypes, we analyzed 143 immune-related gene signatures. We found that the majority of these genes were significantly upregulated in Clusters 1 and 2 compared with Cluster 3 (Figure 6a). We next quantified key biomarkers of anti-tumor immunity: immune score, immune cytolytic activity (CYT), and major histocompatibility complex (MHC) score [33,34]. Clusters 1 and 2 exhibited significantly higher immune, MHC, and CYT scores (all *p* < 0.001; Figure 6b). These findings demonstrate that Clusters 1 and 2 are characterized by enhanced anti-tumor immunity, while Cluster 3 shows an immune-depleted phenotype.

Since an immunosuppressive tumor microenvironment (TME) can drive immune escape and resistance to immune checkpoint blockade (ICB), we employed the TIDE algorithm to explore potential ICB response patterns [30]. Strikingly, Cluster 2 displayed significantly lower TIDE scores than Clusters 1 and 3 (Figure 5c), which is consistent with a lower likelihood of immune evasion as estimated by this computational tool. We further explored this using immunophenoscore (IPS) analysis. Clusters 1 and 2 showed consistently higher IPS scores across multiple treatment scenarios—anti-PD-1 alone, anti-CTLA-4 alone, and their combination—suggesting a potentially more favorable response to ICB (Figure 6c). However, TIDE and IPS are computational estimates and do not substitute for actual clinical response data.

Finally, we contextualized our subtypes within the pan-cancer immune classification system established by Thorsson et al. [32], which includes six immune subtypes (C1–C6). We focused on the C3 (inflammatory) subtype, characterized by a dominant T helper 17 (Th17) signature and favorable prognosis, which is associated with better immunotherapy outcomes. Notably, the proportion of the C3 subtype was markedly higher in Cluster 1 (8.24%) and Cluster 2 (43.47%) than in Cluster 3 (5.91%; Figure 6d). This alignment with an established immunotherapy-responsive immune subtype is consistent with the possibility that patients in Clusters 1 and 2 could be more likely to benefit from immunotherapy, although prospective confirmation is needed.

**Figure 7:**
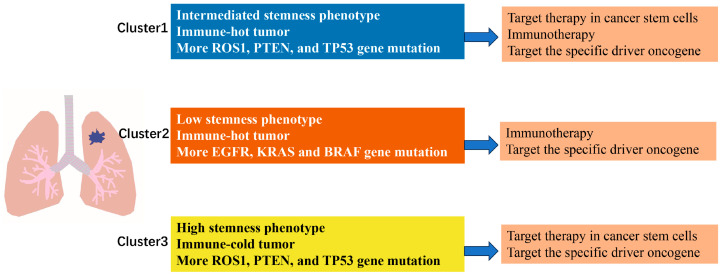
Overview of the characteristics of different NSCLC subtypes and proposed individualized treatment strategies. The three subtypes are color-coded: Cluster 1 (blue), Cluster 2 (orange), and Cluster 3 (yellow). Each subtype is characterized by distinct stemness phenotypes, immune profiles, and driver gene mutation patterns, leading to tailored therapeutic recommendations.

To provide an integrated overview of the three snoRNA-defined NSCLC subtypes, we summarized their key molecular and immune characteristics, along with proposed therapeutic strategies, in a schematic diagram (Figure 7). Clusters 1 and 3 displayed a proliferative/stem-like phenotype with high mutation frequencies in *ROS1*, *TP53*, and *PTEN*, whereas Cluster 2 showed a greater prevalence of *EGFR*, *KRAS*, and *BRAF* mutations. Immunologically, Clusters 1 and 2 exhibited active (“hot”) tumor microenvironments, while Cluster 3 was immune-cold. Based on these features, Clusters 1 and 3 may benefit more from targeted therapies against PI3K/Hippo pathways, whereas Cluster 2 patients might be better candidates for immunotherapy and EGFR/KRAS-targeted agents. This schematic serves as a practical reference for future personalized treatment design.

### 3.7. Generalization of the Immune-Related snoRNA-Based Framework to Head and Neck Squamous Cell Carcinoma (HNSC)

**Figure 8:**
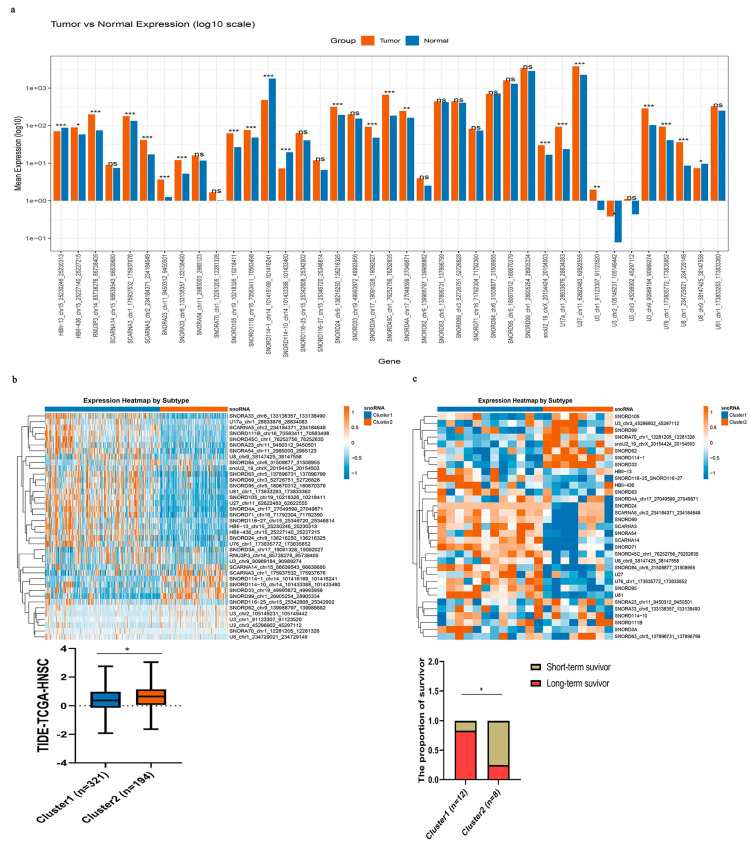
Immune-related snoRNAs could identify different immunotherapy-responsive subtypes in HNSC. (**a**) Differential expression of subtyping-related snoRNAs between tumor (orange) and normal (blue) tissues (log_10_ scale). Significance: * *p* < 0.05, ** *p* < 0.01, *** *p* < 0.001; ns, not significant. (**b**) Two HNSC subtypes defined by immune-related snoRNAs in TCGA. Top: snoRNA expression heatmap (orange: high, blue: low; Cluster 1: blue, Cluster 2: orange). Bottom: TIDE scores (* *p* < 0.05). (**c**) Validation in GSE212550. Top: snoRNA expression heatmap (same color scheme as (**b**)). Bottom: Proportion of long-term survivors in each subtype (* *p* < 0.05, chi-square test).

Applying the same analytical framework (intersection of partial correlation with any immune pathway gene at empirical FDR < 0.25, |r| > 0.2, *p* < 0.05 and GSEA enrichment for any immune pathway at q < 0.25, adjusted *p* < 0.05; followed by TIMER correlation |cor| ≥ 0.3, *p* < 0.05), we identified 39 key immune-related snoRNAs for HNSC. Among these, four snoRNAs (HBII-13, U8, SNORD114-10, and SNORD114-1) were significantly downregulated in tumor tissues, while 20 snoRNAs were markedly upregulated compared with adjacent normal tissues (Figure 8a). Using these 39 immune-related snoRNAs, we classified TCGA-HNSC samples into two distinct molecular subtypes (Figure 8b). Strikingly, Cluster 1 exhibited significantly lower TIDE scores compared with Cluster 2, suggesting a more favorable immune microenvironment. To further validate the clinical relevance of this classification, we applied the same snoRNA-based subtyping to the independent immunotherapy cohort GSE212550. Long-term survival after immunotherapy was significantly more common in Cluster 1 (83.3%) than in Cluster 2 (25.0%) (*p* = 0.021) (Figure 8c). This finding suggests that the snoRNA subtypes may serve as predictive biomarkers for durable immunotherapy responses. However, given the very limited sample size, this observation should be interpreted as preliminary and hypothesis-generating rather than conclusive.

Together, these findings suggest that immune-related snoRNAs could serve as exploratory biomarkers to identify HNSC subtypes with divergent responses to immunotherapy. Subject to further validation, they may help personalize treatment strategies for head and neck cancer patients.

## 4. Discussion

This study proposes a novel conceptual framework by identifying immune-related snoRNAs as potential modulators of the tumor immune microenvironment and candidate markers for molecular subtyping. Moving beyond their conventional role in ribosome biogenesis, our analyses suggest through a comprehensive pan-cancer analysis that snoRNAs are extensively involved in tumor progression and immune regulation. Our key findings indicate that (1) a significant fraction of snoRNAs are functionally linked to core immune pathways; (2) these snoRNAs are highly enriched within immune cells and their expression correlates with immune infiltration; (3) immune-related snoRNAs are frequently dysregulated in pan-cancer and breast CSCs; (4) functional validation using SNORD116-19 suggests its role in suppressing cancer stemness and metastasis in triple-negative breast cancer; and (5) most importantly, we developed a preliminary translational framework utilizing immune-related snoRNAs to stratify NSCLC and HNSC patients into subtypes with distinct molecular profiles and tumor microenvironments, and predicted responses to immunotherapy, though these predictions require prospective testing.

Our study offers a new perspective to the growing field of snoRNA immuno-oncology by defining distinct, cancer-type-specific landscapes of immune-related snoRNAs. Earlier pioneering work by Wang et al. [22] provided valuable pan-cancer insights by identifying 16 snoRNAs correlated with immunotherapy response across 31 cancer types. Similarly, Battaglin et al. [42] demonstrated that specific snoRNAs (SCARNA5, SCARNA6, SCARNA21) possess predictive value for treatment response in metastatic colorectal cancer patients receiving targeted biologic agents, further supporting the clinical potential of snoRNAs as predictive biomarkers beyond conventional prognostic signatures. Complementarily, Cai et al. [21] offered significant depth at a single-cancer level, developing a precise snoRNA-based scoring model for colon cancer that highlights clinical translation potential. More recently, Záveský et al. [43] systematically evaluated snoRNA expression in invasive breast cancer and identified SCARNA2 as a promising prognostic biomarker associated with better progression-free survival, while SNORD94, SNORD15B, and SCARNA3 were implicated as putative tumor suppressors. In the context of NSCLC immunotherapy, Zhuo and colleagues experimentally demonstrated that targeting SNORA38B not only suppressed tumorigenesis but also remodeled the tumor microenvironment to sensitize NSCLC to immune checkpoint blockade, providing direct functional evidence linking a specific snoRNA to immunotherapy response [11]. In comparison with these prior studies, our work provides two distinct contributions. First, while Wang et al. [22] focused on a pan-cancer set of 16 snoRNAs, our cancer type-specific analysis reveals substantial variation across tumor types (i.e., 16.3% to 83.9% of expressed snoRNAs), with particularly high dysregulation in immunotherapy-sensitive cancers such as BLCA, BRCA, and LUSC. Second, unlike Cai et al.’s [21] continuous prognostic score for colon cancer, our molecular subtyping framework classifies tumors into “cold” versus “hot” categories, capturing qualitative immune microenvironment differences that may be more directly actionable for immunotherapy decision-making. However, the pan-cancer approach may obscure cancer-specific functions, while a single-cancer model limits broader applicability. Our analysis attempts to bridge these scales by identifying a landscape of immune-related snoRNAs specific to individual cancer types within the TCGA. This strategy allowed us to observe, using NSCLC and HNSC as examples, that cancer-specific snoRNAs can effectively define molecular subtypes with distinct immune microenvironments and predictable immunotherapy outcomes. Furthermore, the findings were validated in an external GEO dataset. Importantly, our work lends support to the concept proposed by Li et al. [24] that the identification of immune-related non-coding RNAs can be extended beyond lncRNAs. To our knowledge, this is the first study to systematically apply this concept to snoRNAs, suggesting their possible relevance in cancer immunology.

The clinical potential of our findings is suggested by the specific immune pathways—such as antigen presentation, chemokine, and cytokine signaling—with which these snoRNAs are associated, all of which are critically implicated in immunotherapy outcomes [37,38,39]. The possibility to molecularly subtype tumors into “cold” and “hot” categories using a minimal set of snoRNAs may offer a practical approach for potentially informing patient response stratification. The observation that in HNSC, 10 out of 12 patients in snoRNA-defined Cluster 1 achieved long-term survival after immunotherapy in the GSE212550 cohort is preliminary and should be interpreted with caution given the small sample size and retrospective design of this study. Larger prospective cohorts are needed to validate whether this subtype can reliably predict durable immunotherapy benefit.

We posit that snoRNAs possess distinct advantages for future therapeutic exploitation. Their relatively small size and modular nature may make them more tractable targets for manipulation compared with larger ncRNAs such as lncRNAs. The prospect of modulating specific snoRNAs to influence immune cell fate or to convert immunologically “cold” tumors into “hot” ones, akin to strategies targeting proteins such as RGS1 [44], is an exciting direction for future research.

Our study found that SNORD116-19 expression did not significantly differ between breast cancer and normal tissues, nor between breast CSCs and bulk tumor cells. This contrasts with the typical pattern of tumor-suppressive snoRNAs, which are often downregulated in cancer. Nevertheless, its overexpression in TNBC cells strongly suppressed invasion, migration, and stemness. We propose two speculative (but testable) hypotheses to explain this apparent discrepancy. First, SNORD116-19 may function as a constitutively expressed “housekeeping” snoRNA whose core RNA-modifying activity is functionally silenced in cancer (i.e., via altered binding partners or subcellular localization); exogenous overexpression might bypass this silencing and restore tumor suppression. Second, its activity may be context-dependent: under tumor-specific stress signaling (i.e., hyperactive MYC or PI3K pathways), the rRNA modification it catalyzes could become critical for maintaining proteostasis and survival, making it an “Achilles’ heel” that exogenous overexpression can disrupt. These hypotheses require direct experimental testing, such as measuring its target rRNA modification efficiency or identifying its dynamic protein interactome in different oncogenic contexts. Importantly, these mechanistic speculations do not diminish the key observation that immune-related snoRNAs as a class are frequently dysregulated in CSCs and may represent novel therapeutic nodes.

Despite these conceptual and translational advances, this study has several limitations.

First, the experimental validation is preliminary and hypothesis-generating. The proposed mechanisms underlying SNORD116-19 function remain speculative because functional tests were confined to overexpression experiments in a single TNBC cell line and did not examine broader immune-regulatory roles. Key controls are lacking, including loss-of-function assays, rescue experiments, subcellular localization analyses, and direct evidence of rRNA modification (e.g., RiboMethSeq). Therefore, the functional conclusions must be interpreted cautiously. Furthermore, the RNA-seq results were not validated using orthogonal methods (e.g., qPCR or protein assays), and only one immune-related snoRNA was tested. We reiterate that the primary objective of this study is bioinformatic identification; accordingly, the experimental data serve only as proof-of-concept.

Second, the snoRNA-based NSCLC subtyping model shows only moderate internal stability. The mean cross-validation ARI was 0.51, indicating a potential risk of overfitting. This is likely due to the relatively small sample size (*n* = 1044) and the intrinsic high molecular heterogeneity of NSCLC. Consequently, future studies should employ larger independent cohorts and a more parsimonious set of snoRNA features to improve generalizability.

Third, associations with immunotherapy response are derived from computational predictions, not clinical outcomes. The TIDE and IPS algorithms, albeit widely used for hypothesis generation, have well-recognized limitations and cannot replace direct assessment of response to immune checkpoint inhibitors. Although we observed consistency in an external cohort (GSE212550), the sample size was small. Thus, prospective studies with actual patient response data are required to validate the predictive value of our subtyping framework.

Fourth, technical limitations of snoRNA detection in bulk RNA-seq may bias expression estimates. Standard RNA-seq libraries capture snoRNAs inefficiently because of their short length and the absence of dedicated small-RNA library preparation, which may lead to underestimation of expression and increased false negative findings.

Fifth, computational estimates of immune cell infiltration carry inherent errors. Algorithms such as TIMER and CIBERSORT are influenced by tumor purity, batch effects, and cellular heterogeneity, all of which may bias correlation analyses between snoRNAs and immune cell abundance.

Sixth, therapeutic targeting of snoRNAs faces practical challenges. Delivery efficiency and specificity remain unresolved issues that must be addressed in future translational research.

## 5. Conclusions

In summary, this study suggests that immune-related snoRNAs may be important molecular players with dual roles in regulating tumor progression and the anti-tumor immune response. Through a systematic pan-cancer analysis, we have delineated a comprehensive landscape of snoRNAs functionally linked to core immunological pathways and observed their prevalent dysregulation across cancers, including within cancer stem cells. Functional validation using SNORD116-19 indicates the capacity of these molecules to suppress stemness and metastatic potential. Most importantly, we propose a preliminary translational framework based on a minimal set of immune-related snoRNAs, which may help stratify NSCLC and HNSC patients into molecular subtypes with distinct tumor microenvironments and suggest differential responses to immunotherapy. This classification identified patient subgroups with superior survival benefits in independent cohorts, though these findings are retrospective and require prospective validation. Our findings suggest a potential expansion of the functional paradigm of snoRNAs in oncology and point toward a candidate biomarker strategy that might eventually inform personalized cancer immunotherapy, pending further mechanistic and clinical studies.

## Figures and Tables

**Table 1:** Overlap of snoRNAs identified by TIMER with CIBERSORT, expressed as *p*-values.

Cancers	B Cells	Macrophages	Dendritic Cells	Neutrophil	CD4+ T Cells	CD8+ T Cells
ACC	0	9.64 × 10^−7^	0.000514855	4.89 × 10^−31^	0.8716862	1.05 × 10^−2^
BLCA	0.001725197	1.86 × 10^−24^	0.5434576	9.84 × 10^−1^	6.25 × 10^−24^	1.83 × 10^−1^
BRCA	7.31 × 10^−23^	9.52 × 10^−16^	5.32 × 10^−2^	3.73 × 10^−2^	4.38 × 10^−16^	1.83 × 10^−12^
CESC	1.36 × 10^−3^	7.95 × 10^−1^	8.20 × 10^−4^	6.09 × 10^−1^	4.17 × 10^−1^	1.70 × 10^−4^
CHOL	3.37 × 10^−124^	7.75 × 10^−2^	0.2781415	0.8881242	2.71 × 10^−123^	0.5831955
COAD	0.000134637	1.11 × 10^−37^	1.58 × 10^−8^	7.67 × 10^−8^	3.37 × 10^−21^	8.04 × 10^−7^
DLBC	0.2859313	0.000146168	0.9968618	0.7929443	8.69 × 10^−7^	0.2936191
ESCA	3.73 × 10^−56^	2.91 × 10^−12^	0.1115102	0.09755062	0.407884	5.57 × 10^−6^
HNSC	1.02 × 10^−51^	4.92 × 10^−28^	0.4231745	0.8840514	9.70 × 10^−28^	1.04 × 10^−43^
KICH	0.9964794	3.34 × 10^−10^	0.9900563	0.8145457	0.000144348	0.7792685
KIRC	1.08 × 10^−9^	5.12 × 10^−9^	0.000162787	0.03466863	0.07561106	2.38 × 10^−12^
KIRP	1.80 × 10^−15^	1.72 × 10^−5^	0.1581215	6.13 × 10^−6^	9.93 × 10^−12^	0.000141409
LGG	5.19 × 10^−27^	5.47 × 10^−18^	0.4371587	0.1834694	5.85 × 10^−16^	0.0014817
LIHC	2.66 × 10^−7^	1.61 × 10^−5^	0.6020427	0.001582464	0.003700548	2.66 × 10^−10^
LUAD	4.87 × 10^−35^	2.62 × 10^−7^	0.1978027	0.000417827	1.91 × 10^−14^	1.09 × 10^−5^
LUSC	5.52 × 10^−17^	2.06 × 10^−11^	0.04039687	0.0128479	3.17 × 10^−57^	2.94 × 10^−5^
MESO	1.21 × 10^−114^	0.005740786	0.005558234	0.04509911	0.000180287	2.19 × 10^−12^
OV	0.5829572	0.006828906	0.000938342	0.08519513	0.000634034	0.4575554
PAAD	1.72 × 10^−79^	2.91 × 10^−15^	0.5498204	0.9969005	2.56 × 10^−27^	0.02671072
PCPG	0.5813277	0.006037756	0.7731533	0.6787109	0.05909795	9.33 × 10^−10^
PRAD	7.87 × 10^−8^	1.21 × 10^−43^	0.9397916	0.02500553	5.15 × 10^−29^	1.51 × 10^−33^
READ	0.05658329	7.54 × 10^−33^	0.01158724	3.41 × 10^−17^	0.2979904	1.23 × 10^−25^
SARC	0.01173038	0.09849348	2.49 × 10^−5^	0.000757975	3.84 × 10^−5^	5.80 × 10^−9^
SKCM	1.78 × 10^−33^	6.17 × 10^−7^	0.03962026	0.8109472	5.21 × 10^−12^	1.50 × 10^−12^
STAD	2.27 × 10^−10^	1.57 × 10^−14^	0.1067971	0.000174964	1.69 × 10^−33^	0.007622005
TGCT	7.01 × 10^−15^	0.000853644	0.05542729	0.5922325	4.51 × 10^−69^	3.38 × 10^−32^
THCA	5.90 × 10^−15^	0.000668974	1.23 × 10^−22^	0.000172601	1.41 × 10^−20^	1.46 × 10^−5^
THYM	1.40 × 10^−12^	6.65 × 10^−12^	0.008992865	0.9999996	6.97 × 10^−20^	0.3936162
UCEC	6.90 × 10^−9^	0.08134059	0.001168213	0.008084847	0.001920156	3.29 × 10^−6^
UCS	2.10 × 10^−5^	3.33 × 10^−6^	1.34 × 10^−7^	1.19 × 10^−5^	0.19475	0.6510826
UVM	0.08105518	0.8726819	0.01450239	0.7265488	0.4072468	3.47 × 10^−6^

## Data Availability

The processed expression matrices for 31 cancer types, the immune-related snoRNA lists per cancer type, the molecular subtype assignment files for NSCLC and HNSC, the full analysis pipeline and the source data for figures and tables have been deposited in the Science Data Bank (ScienceDB, https://www.scidb.cn, accessed 4 May 2026) under https://doi.org/10.57760/sciencedb.35912 and are publicly available as of the date of publication. All raw sequencing data from public sources are accessible via TCGA (https://cancergenome.nih.gov, accessed 15 March 2023) and GEO (https://www.ncbi.nlm.nih.gov/geo, accessed 15 March 2023). The DOI link will become active upon publication; for peer review, a private access link is available at https://doi.org/10.57760/sciencedb.35912. This study did not generate new unique reagents.

## Supplementary Materials

The following supporting information can be downloaded at https://www.mdpi.com/article/10.3390/genes17050581/s1, Table S1. The TCGA cancer types used in our analysis; Table S2. The 17 immunologically significant gene sets; Table S3. Sensitivity analysis of partial correlation, FDR, and *p*-value thresholds on the number of robust snoRNA–immune gene pairs in breast cancer (BRCA); Table S4. The top 500 SnoRNA-pathway pairs in different cancer types; Table S5. Multi omics and clinicopathological distribution of three snoRNA based subtypes in NSCLC; Figure S1. Sensitivity analysis of partial correlation, FDR, and *p* value thresholds in breast cancer (BRCA). The pipeline remained highly robust across all tested thresholds (pair count ≥ 9976, Jaccard ≥ 0.999, top 100 retention ≥ 0.92), with only a partial correlation >0.30 reducing the pair count to 2777; Figure S2. Consensus clustering of NSCLC samples using 46 immune related snoRNAs. (A) Consensus cumulative distribution function (CDF) curves for cluster numbers k = 2 to 12. (B) Relative change in the area under the CDF curve (delta area) for each k. Both panels support k = 3 as the optimal number of stable molecular subtypes; Figure S3. Ten fold cross validation of snoRNA based subtypes. The mean Adjusted Rand Index (ARI) between training and testing folds was 0.501 ± 0.081, demonstrating stable clustering performance; Figure S4. GSVA heatmap of pathway activity across snoRNA subtypes. Pathways were included if they were significantly different in at least one pairwise comparison among the three subtypes. Cluster 1 and Cluster 3 showed enrichment of proliferative pathways, while immune pathways were predominantly enriched in Cluster 1 and particularly Cluster 2.

## Abbreviations

ACC, adrenocortical carcinoma; AD, adenocarcinoma subtype; ALDH, aldehyde dehydrogenase; APC, Antigen Processing and Presentation; ARI, adjusted Rand index; BLCA, bladder urothelial carcinoma; BRCA, breast cancer; CDF, cumulative distribution function; CESC, cervical squamous cell carcinoma and endocervical adenocarcinoma; CHOL, cholangiocarcinoma; COAD, colorectal adenocarcinoma; COCA, ConsensusOmics Classification; CSC, cancer stem cell; CTL, cytotoxic T lymphocyte; CYT, cytolytic activity; DEG, differentially expressed gene; DFI, disease-free interval; DLBC, diffuse large B-cell lymphoma; DSS, disease-specific survival; ECM, extracellular matrix; EMT, epithelial–mesenchymal transition; ESCA, esophageal carcinoma; FDR, false discovery rate; GEO, Gene Expression Omnibus; GO, Gene Ontology; GSEA, gene set enrichment analysis; GSVA, Gene Set Variation Analysis; HNSC, head and neck squamous cell carcinoma; ICI, immune checkpoint inhibitor; IPS, immunophenoscore; KICH, kidney chromophobe; KIRC, kidney renal clear cell carcinoma; KIRP, kidney renal papillary cell carcinoma; LGG, low-grade glioma; LIHC, liver hepatocellular carcinoma; lncRNA, long non-coding RNA; LUAD, lung adenocarcinoma; LUSC, lung squamous cell carcinoma; MAD, median absolute deviation; MESO, malignant mesothelioma; MHC, major histocompatibility complex; ncRNA, non-coding RNA; NSCLC, non-small-cell lung cancer; OR, odds ratio; OS, overall survival; OV, ovarian cancer; PAAD, pancreatic adenocarcinoma; PCPG, pheochromocytoma and paraganglioma; PFI, progression-free interval; PRAD, prostate adenocarcinoma; qPCR, quantitative polymerase chain reaction; READ, rectal adenocarcinoma; SARC, sarcoma; SCC, Spearman correlation coefficient; SKCM, cutaneous melanoma; snoRNA, small nucleolar RNA; SQ, squamous cell carcinoma subtype; STAD, stomach adenocarcinoma; TCGA, The Cancer Genome Atlas; TCIA, The Cancer Immunome Atlas; TGCT, testicular germ cell tumor; THCA, thyroid cancer; THYM, thymoma; TIDE, Tumor Immune Dysfunction and Exclusion; TIMER, Tumor Immune Estimation Resource; TME, tumor microenvironment; TNBC, triple-negative breast cancer; UCEC, uterine corpus endometrial carcinoma; UCS, uterine carcinosarcoma; UVM, uveal melanoma.
