# Supplementary material for: Systematic Identification of Immune-Related SnoRNAs: Potential Dual Roles in Tumor Progression and Immunotherapy Response
Source: Genes (Basel). 2026 May 18;17(5):581. doi: 10.3390/genes17050581 (PMC13205779; doi:10.3390/genes17050581)
Supplement: Supplementary file 1 [file genes-17-00581-s001.zip › genes-4250318-supplementary.pdf]

Supplement Materials and Methods

1.1 Plasmid Construction and Stable Cell Line Establishment

The full-length sequence of SNORD116-19 was amplified by PCR from reverse transcription products and subsequently cloned into the PHY-023 lentiviral vector (Hanyin). The constructed plasmid was verified by DNA sequencing to ensure accuracy. Following sequence confirmation, lentiviruses were packaged and used to transduce BRCA cells. Successfully transduced cells were selected using 0.35 mg/ml puromycin. The overexpression efficiency of SNORD116-19 was ultimately assessed by real-time quantitative RT-PCR (RT-qPCR).

1.2 Mammosphere formation assay

Cells were washed and resuspended with stem cell culture medium, which contained serum-free DMEM-F12 (Hyclone), supplemented with B27 (1:50, Invitrogen), 10 ng/mL EGF (BD Biosciences), 20 ng/mL bFGF (PeproTech), and 4 mg/mL insulin (Sigma). Then single cells were planted on ultralow attachment plates (Corning) at a density of 2*105 cells/cm2 and incubated in a 5% CO2 incubator at 37 C for 7 days. Afterward, the size and number of mammospheres were determined. Only mammospheres that were over 60 μm diameter were recorded.

1.3 Colony formation assay

4000 cells were plated on a 6-well plate and cultured in DMEM containing 10% FBS for 2 weeks. Cell colonies were fixed with 4% paraformaldehyde and stained with crystal violet for 30 min. The colonies were counted digitally using ImageJ software.

1.4 Cell migration and invasion assay

For the cell invasion assay, the transwell plates with 8 μm pore size polycarbonate filters were precoated with 100 ul/well diluted Matrigel. Then, cells (1 × 105 /well) were seeded with serum-free media into the upper chamber of transwell filter. The lower chamber was supplied with 600 μL media with 10% FBS. After incubation for 24 h,

cells on the upper side of the filters were removed. Then, the filters were fixed with 4% paraformaldehyde, stained with crystal violet for 30 min and couted under a microscope. The data were expressed as the average cell number of five randomly chosen fields. For the migration assays, the protocol was similar to the cell invasion assay except that the precoated filters was omitted.

1.5 Aldefluor Assay for ALDH Activity

We performed the Aldefluor assay to quantify ALDH activity using the commercial kit (STEMCELL Technologies). In brief, we resuspended 1 × 10^5 cells in the substrate-containing buffer, with a separate aliquot treated with DEAB as an inhibitor control. After a 30-minute incubation at 37 °C in the dark, cells were washed and analyzed on a FACSCalibur flow cytometer (BD Biosciences).

1.6 Transcriptome Profiling and Functional Enrichment

We performed RNA sequencing on SNORD116-19-overexpressing (OE) and negative control (NC) MDA-MB-231 cells via Majorbio Company (Shanghai, China). Differential expression analysis was conducted with the "limma" package, defining DEGs as genes with $|\log2FC| > 1.5$ and adjusted p-value < 0.05. To interpret the biological impact, we performed GO and KEGG enrichment analyses on the DEGs using "clusterProfiler", considering terms with an adjusted p-value < 0.05 as significant.

1.7 RNA Extraction and snoRNA Quantification by RT-qPCR

Total RNA was isolated from TNBC cell lines using Trizol reagent (Thermo Fisher Scientific) according to the manufacturer's protocol. RNA concentration and purity were assessed by measuring the A260/A280 ratio on a Synergy 2 multi-plate reader (BioTek). Subsequently, 25 ng of small RNA from each sample was reverse-transcribed into cDNA in a 10 μl reaction volume using the Tiangen MicroRNA Reverse Transcription Kit. The resulting cDNA products were diluted 1:9 with nuclease-free water and used as templates for quantitative PCR (qPCR). qPCR was performed using TaqMan Universal PCR Master Mix (Applied Biosystems) with snoRNA-specific

primers and the commercial U6 primer as an endogenous control. All reactions were run in triplicate. The relative expression level of each snoRNA was normalized to U6 and calculated using the 2^(-ΔCt) method, where ΔCt represents the difference in threshold cycles between the target snoRNA and U6.

Supplement Figures and Tables

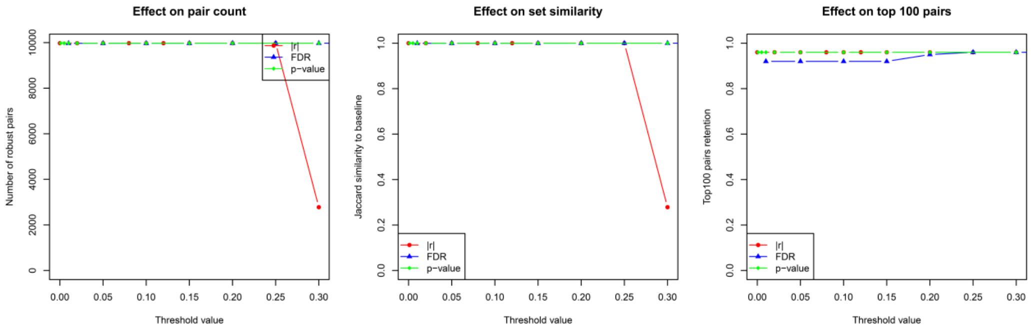

Figure S1. Sensitivity analysis of partial correlation, FDR, and p-value thresholds in breast cancer (BRCA). The pipeline remained highly robust across all tested thresholds (pair count ≥ 9,976, Jaccard ≥ 0.999, top-100 retention ≥ 0.92), with only a partial correlation >0.30 reducing the pair count to 2,777.

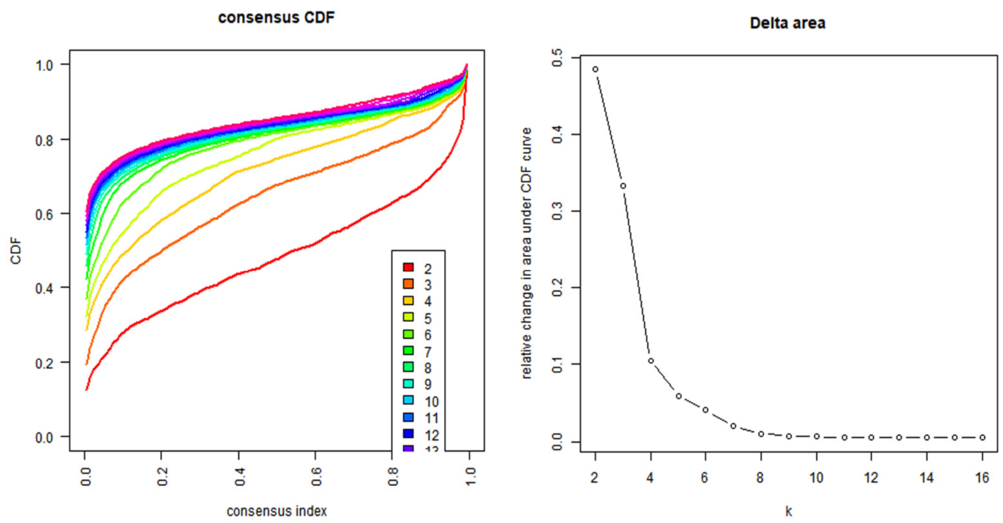

Figure S2. Consensus clustering of NSCLC samples using 46 immune-related snoRNAs. (A) Consensus cumulative distribution function (CDF) curves for cluster numbers k=2 to 12. **(B)** Relative change in the area under the CDF curve (delta area) for each k. Both panels support k=3 as the optimal number of stable molecular subtypes.

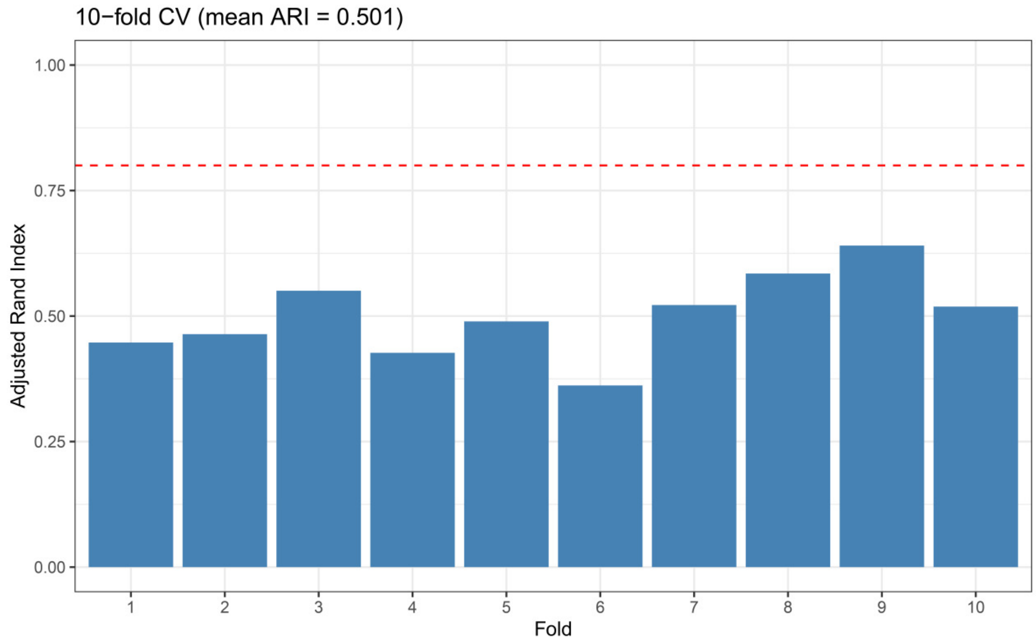

Figure S3. Ten-fold cross-validation of snoRNA-based subtypes.

The mean Adjusted Rand Index (ARI) between training and testing folds was 0.501 ± 0.081, demonstrating stable clustering performance.

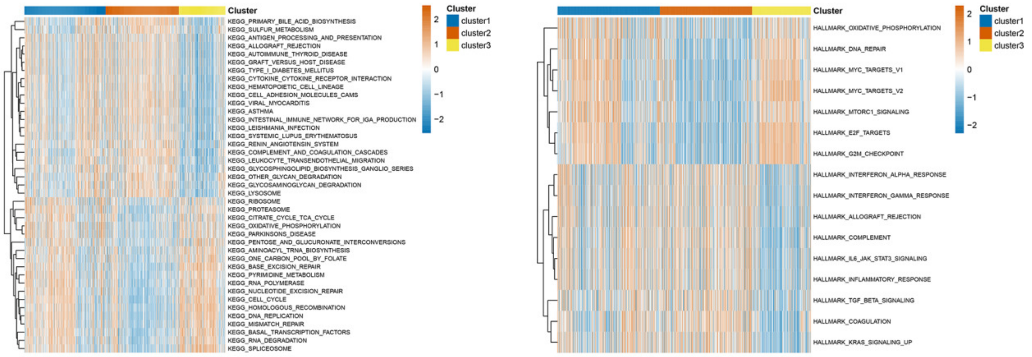

Figure S4. GSVA heatmap of pathway activity across snoRNA subtypes.

Pathways were included if they were significantly different in at least one pairwise comparison among the three subtypes. Cluster 1 and Cluster 3 showed enrichment of proliferative pathways, while immune pathways were predominantly enriched in Cluster 1 and particularly Cluster 2.

Table S1. The TCGA cancer types used in our analysis.

Full name	Abbreviation	Number of Cancer Samples	Number of Normal Samples	ALL
Adrenocortical carcinoma	ACC	80	0	80
Bladder Urothelial Carcinoma	BLCA	397	16	413
Breast invasive carcinoma	BRCA	1077	104	1181
Cervical squamous cell carcinoma and endocervical adenocarcinoma	CESC	295	3	298
Cholangiocarcinoma	CHOL	36	9	45
Colon adenocarcinoma	COAD	433	1	434
Lymphoid Neoplasm Diffuse Large B-cell Lymphoma	DLBC	47	0	47
Esophageal carcinoma	ESCA	184	11	195
Head and Neck squamous cell carcinoma	HNSC	523	44	567
Kidney Chromophobe	KICH	66	25	91
Kidney renal clear cell carcinoma	KIRC	516	71	587
Kidney renal papillary cell carcinoma	KIRP	290	34	324
Brain Lower Grade Glioma	LGG	512	0	512
Liver hepatocellular carcinoma	LIHC	372	50	422
Lung adenocarcinoma	LUAD	513	46	559
Lung squamous cell carcinoma	LUSC	476	45	521
Mesothelioma	MESO	87	0	87
Ovarian serous cystadenocarcinoma	OV	466	0	466
Pancreatic adenocarcinoma	PAAD	178	4	182
Pheochromocytoma and Paraganglioma	PCPG	179	3	182
Prostate adenocarcinoma	PRAD	483	52	535
Rectum adenocarcinoma	READ	160	0	160
Sarcoma	SARC	246	0	246
Skin Cutaneous Melanoma	SKCM	447	2	449
Stomach adenocarcinoma	STAD	409	37	446
Testicular Germ Cell Tumors	TGCT	150	0	150
Thyroid carcinoma	THCA	510	71	581
Thymoma	THYM	124	2	126
Uterine Corpus Endometrial Carcinoma	UCEC	538	33	571
Uterine Carcinosarcoma	UCS	57	0	57
Uveal Melanoma	UVM	80	0	80

Table S2. The 17 immunologically significant gene sets.

Pathway	Gene name	The number of genes
Antigen Processing and Presentation	AZGP1;B2M;CALR;CANX;CD1A;CD1B;CD1C;CD1D;CD1E;CD4;CD8A;CD8B;CD74;CREB1;CTSB;CTSE;CTSS;FCER1G;FCGRT;PDIA3;HFE;HLA-A;HLA-B;HLA-C;HLA-DMA;HLA-DMB;HLA-DOA;HLA-DOB;HLA-DPA1;HLA-DPB1;HLA-DQA1;HLA-DQA2;HLA-DQB1;HLA-DRA;HLA-DRB1;HLA-DRB5;HLA-E;HLA-F;HLA-G;MR1;HSPA1A;HSPA1B;HSPA1L;HSPA2;HSPA4;HSPA5;HSPA6;HSPA8;HSP90AA1;HSP90AB1;ICAM1;IFNA1;IFNA2;IFNA4;IFNA5;IFNA6;IFNA7;IFNA8;IFNA10;IFNA13;IFNA14;IFNA16;IFNA17;IFNA21;IFNG;KIR2DL1;KIR2DL3;KIR2DL4;KIR3DL1;KIR3DL2;KLRC1;KLRC2;KLRC3;KLRD1;LTA;CIITA;MICA;MICB;NFYA;NFYB;NFYC;LGMN;PSMB8;PSMC1;PSMC2;PSMC3;PSMC4;PSMC5;PSMC6;PSMD1;PSMD2;PSMD3;PSMD4;PSMD5;PSMD7;PSMD8;PSMD10;PSMD11;PSMD13;PSME1;PSME2;RELB;RFX5;RFXAP;SLC10A2;TAP1;TAP2;TAPBP;THBS1;KLRC4;AP3B1;RFXANK;PSMD6;PSME3;PSMD14;CLEC4M;IFI30;PROCR;ADRM1;KIAA0368;TRPC4AP;CD209;UBXN1;ERAP1;TAPBPL;ERAP2;ULBP3;ULBP2;ULBP1;KIR3DL3;RAET1E;RAET1L;UBR1;RAET1G;PDIA2	135

Pathway	Gene name	The number of genes
Antimicrobials	HAMP;PI3;CAMP;PPBP;REG3G;CXCL14;CXCL16;SLPI;CXCL10;CXCL9;CXCL5;CXCL11;CXCL6;CXCL1;CXCL12;CXCL13;CXCL2;PF4;XCL1;CXCL3;DEFB103A;CCL13;CCL1;DEFB1;CCL8;ELANE;DEFB103B;DEFA3;DEFA1;TMSB10;DEFA6;DEFA5;DEFA4;LCN2;LCN1;COLEC10;BPI;S100A9;S100A8;DCD;LCN6;S100A12;HTN3;LCN8;CCR10;CELA1;DEFB106A;PENK;MMP12;LEAP2;SFTPD;LCN9;PTGDS;TMSB4X;PGLYRP1;ZC3HAV1;TMSB15A;S100B;S100A13;S100A6;DEFB119;DEFB107A;DEFB105A;SERPIND1;DEFB129;DEFB127;S100P;S100A7;DEFB104A;DEFB126;DEFB106B;DEFB104B;DEFB107B;PGLYRP3;PGLYRP2;S100A10;S100A2;DEFB125;DEFB123;DEFB105B;DEFB132;LCN12;PGLYRP4;S100A11;S100A5;S100A3;S100A1;DEFB128;DEFB108B;HTN1;LMBR1L;S100A7A;DEFB118;COLEC12;TMSB4Y;DEFB131;DEFB134;DEFB130;DEFB124;DEFB121;DEFB116;DEFB115;DEFB114;DEFB113;DEFB112;DEFB110;TMSB15B;DEFB133;S100Z;MAVS;S100A14;LCN10;S100A16;DEFB136;ZC3HAV1L;S100A7L2;IFNAR1;AZU1;S100G;TCHHL1;TINAGL1;IFNGR1;SLC22A17;WFIKKN1;WFDC2;IL6;UMODL1;TGFB1;PF4V1;MMP9;TLR4;IFNG;SPAG11B;A2M;NFKB1;APOBEC3G;FABP6;NOD2;MBL2;RBP1;TLR2;SLC40A1;PLAU;IL1B;PAEP;HFE2;MUC5AC;CTSS;OBP2A;PLTP;MX1;DDX58;IRF3;SFTPA2;LPA;LBP;RBP4;SFTPA1;NOX4;LTF;IFNB1;RBP5;FABP7;FABP5;FABP3;FABP2;FABP4;R3HDML;OASL;CRABP2;CRABP1;RBP7;DUOX1;OBP2B;RBP2;LCN15;CETP;FABP12;FABP9;LCNL1;C8G;SPAG11A;PI15;NOX1;PMP2;APOD;ORM2;ORM1;TNF;CTSG;PRTN3;MAPK1;PML;AEN;CYBB;ISG20;BCL3;ISG20L2;NOX5;NOX3;DUOX2;TLR3;TFRC;IFIH1;LRP1;TRIM5;IDO1;GDF15;NEDD4;ADIPOQ;STAT3;STAT1;SOCS3;SEMG1;TNFSF10;CCL20;SOCS1;RNASEL;IRF1;IL15;APOBEC3F;RARRES3;CHIT1;IFNA1;CD40;TLR7;PPIA;HFE;ZYX;NLRX1;PGC;VEGFA;IKBKE;ISG15;DHX58;TNFAIP3;TFR2;FCN2;MUC4;F2R;ELN;IL27;MAPT;LYZ;CCL5;LEP;CYLD;KLKB1;CST4;CSRP1;MAPK14;JUN;ITGAV;IRF5;CCR6;IL12B;TLR8;GNLY;CD81;EIF2AK2;APOM;CACYBP;NOD1;MAPK8;MAPK3;BST2;BPHL;PLA2G2A;GRN;PDGFRA;GNAI1;WNT5A;FURIN;ADAR;TYK2;NOS2;TRAF3;TPT1;TPM2;NEO1;AHNAK;TLR1;TK2;PRDX2;MX2;FGF2;FGA;TCF7L2;F2RL1;MSR1;NFKBIZ;LMBR1;SRC;MPO;ELAVL1;ROBO3;SP1;SOD1;PDF;DLL4;ECD;SLC11A1;DMBT1;TMEM173;SKIV2L;SEMG2;LTA;DES;DCK;DAXX;TNFRSF10A;TNFRSF10B;EED;CCL4;LIMS1;LALBA;APOBEC3H;TMPRSS6;SPINK5;MARCO;BECN1;TNFSF11;KNG1;CSK;KLRK1;KCNH2;JUND;JAK1;CREB1;CLDN4;CCL28;RNASE3;IRF7;IREB2;ILK;IL18;IL17A;LTB4R;APOBEC3A;MASP2;TRIM27;RELA;IL7R;IL1A;PTX3;IFNAR2;SYTL1;APOBEC3C;DDX17;PTGS2;HTR1A;CD40LG;CD14;CD8A;CD4;MASP1;PROC;MAP2K2;MAP2K1;HRG;NDRG1;IRF9;TRIM22;LANCL1;PPP4C;HMOX1;HMGB1;HLA-B;RNASE7;ABCC4;HGF;HDAC1;PLSCR1;B2M;BACH2;TANK;PIK3CG;ARRB1;RSAD2;STAB2;TBK1;PDYN;PDGFRB;PDCD1;PCSK2;PCSK1;ARG2;AQP9;FASLG;APOH;BIRC5;ANXA6;IL22;VTN;VIM;VCAM1;PRDX1;GFAP;GBP2;ALB;SLC29A3;OAS1;AGER;UNC93B1;TNFSF4;NOS1;ACTG1;ACTA1;ACO1;SERPINA3;CCL15;CCL14;CCL16;CCL19;CCL18;CCL17;CCL26;CCL22;CCR3;CCL4L2;CCR7;CCL27;CCR8;CCL2;CCL21;CCL7;CCL3;CCL11;CCR5;CCL23;CCL25;CCL3L3;CCR1;CCL24;XCL2;CXCR4;CXCR6;CCR4;FAM19A5;FAM19A3;FAM19A4;FAM19A1;FAM19A2;PTK2B;IL4;CDH1;LTBP1;IL13;IL10;IL2;PPARG;FGR;MIF;CRP;JAK2;PTK2;PTGDR;CD86;HCK;VDR;OLR1;TXK;RNASE2	463
BCR Signaling Pathway	CD79A;CD79B;LYN;SYK;BTK;BLNK;VAV3;VAV1;VAV2;RAC1;RAC2;RAC3;PPP3CA;PPP3CB;PPP3CC;PPP3R1;PPP3R2;CHP2;NFAT5;NFATC1;NFATC2;NFATC3;NFATC4;HRAS;KRAS;NRAS;FOS;JUN;CARD11;BCL10;MALT1;CHUK;IKBKB;IKBKG;NFKB1;RELA;NFKBIA;NFKBIB;NFKBIE;CD81;CD19;CR2;PIK3R5;PIK3R1;PIK3R2;PIK3R3;PIK3CA;PIK3CB;PIK3CD;PIK3CG;AKT3;AKT1;AKT2;GSK3B;INPP5D;CD22;CD72;PTPN6;LILRB3;FCGR2B;RASGRP3;PLCG2;PRKCB;IFITM1	64

Pathway	Gene name	The number of genes
Chemokines	C3;C5;CAMP;CCL1;CCL11;CCL13;CCL14;CCL15;CCL16;CCL17;CCL18;CCL19;CCL2;CCL20;CCL21;CCL22;CCL23;CCL24;CCL25;CCL26;CCL27;CCL28;CCL3;CCL3L3;CCL4;CCL4L2;CCL5;CCL7;CCL8;CKLF;CMA1;CTSG;CX3CL1;CXCL1;CXCL10;CXCL11;CXCL12;CXCL13;CXCL14;CXCL16;CXCL17;CXCL2;CXCL3;CXCL5;CXCL6;CXCL9;CYR61;DEFA1;DEFA3;DEFA5;DEFB1;DEFB103A;DEFB104A;EDN1;EDN2;EDN3;FGF10;FGF2;HTN3;LECT2;PF4;PF4V1;PLAU;PPBP;PROK2;RNASE2;SAA1;SAA2;SBDS;SEMA3A;SEMA3B;SEMA3C;SEMA3D;SEMA3E;SEMA3F;SEMA3G;SEMA4A;SEMA4B;SEMA4C;SEMA4D;SEMA4F;SEMA4G;SEMA5A;SEMA5B;SEMA6A;SEMA6B;SEMA6C;SEMA6D;SEMA7A;SLIT1;SLIT2;TNC;TYMP;XCL1;XCL2	95
Chemokine Receptors	C5AR1;CCR1;CCR10;CCR3;CCR4;CCR5;CCR6;CCR7;CCR8;CCR9;CCRL2;CMKLR1;CX3CR1;CXCR3;CXCR4;CXCR5;CXCR6;CYSLTR1;CYSLTR2;EDNRA;EDNRB;FPR1;FPR2;GPR17;GPR32;GPR33;LTB4R;LTB4R2;PLAUR;PLXNA1;PLXNA2;PLXNA3;PLXNA4;PLXNB1;PLXNB2;PLXNB3;PLXNC1;PLXND1;PTAFR;ROBO1;ROBO2;ROBO3;RXFP3;XCR1	44
Cytokines	ADIPOQ;ADM;ADM2;AGRP;AGT;AMBN;AMELX;AMH;ANGPTL5;ANGPTL7;APLN;AREG;ARTN;AVP;AZU1;BDNF;BMP1;BMP10;BMP15;BMP2;BMP3;BMP4;BMP5;BMP6;BMP7;BMP8A;BMP8B;BTC;C3;C5;CALCA;CALCB;CAMP;CAT;CCK;CCL1;CCL11;CCL13;CCL14;CCL15;CCL16;CCL17;CCL18;CCL19;CCL2;CCL20;CCL21;CCL22;CCL23;CCL24;CCL25;CCL26;CCL27;CCL28;CCL3;CCL3L3;CCL4;CCL4L2;CCL5;CCL7;CCL8;CD320;CD40LG;CD70;CECR1;CER1;CGA;CGB1;CGB2;CGB5;CGB7;CGB8;CHGA;CHGB;CKLF;CLCF1;CLEC11A;CMA1;CMTM1;CMTM2;CMTM3;CMTM4;CMTM5;CMTM6;CMTM7;CMTM8;CNTF;CORT;CRH;CSF1;CSF2;CSF3;CSH1;CSH2;CSHL1;CSPG5;CTF1;CTGF;CTSG;CX3CL1;CXCL1;CXCL10;CXCL11;CXCL12;CXCL13;CXCL14;CXCL16;CXCL17;CXCL2;CXCL3;CXCL5;CXCL6;CXCL9;CYR61;DEFA1;DEFA3;DEFA5;DEFB1;DEFB103A;DEFB104A;DKK1;EBI3;EDN1;EDN2;EDN3;EGF;EPGN;EPO;EREG;ESM1;FAM3B;FAM3C;FAM3D;FASLG;FGF1;FGF10;FGF11;FGF12;FGF13;FGF14;FGF16;FGF17;FGF18;FGF19;FGF2;FGF20;FGF21;FGF22;FGF23;FGF3;FGF4;FGF5;FGF6;FGF7;FGF8;FGF9;FIGNL2;FLT3LG;FSHB;GAL;GALP;GAST;GCG;GDF1;GDF10;GDF11;GDF15;GDF2;GDF3;GDF5;GDF6;GDF7;GDF9;GDNF;GH1;GH2;GHRH;GHRL;GIP;GKN1;GMFB;GMFG;GNRH1;GNRH2;GPHA2;GPHB5;GPI;GREM1;GREM2;GRN;GRP;GUCA2A;HAMP;HBEGF;HDGF;HGF;HTN3;IAPP;IFNA1;IFNA10;IFNA13;IFNA14;IFNA16;IFNA17;IFNA2;IFNA21;IFNA4;IFNA5;IFNA6;IFNA7;IFNA8;IFNB1;IFNE;IFNG;IFNK;IFNW1;IGF1;IGF2;IL10;IL11;IL12A;IL12B;IL13;IL15;IL16;IL17A;IL17B;IL17C;IL17D;IL17F;IL18;IL19;IL1A;IL1B;IL1F10;IL1RN;IL2;IL20;IL21;IL22;IL23A;IL24;IL25;IL26;IL27;IL3;IL31;IL32;IL33;IL34;IL4;IL5;IL6;IL6ST;IL7;IL9;INHA;INHBA;INHBB;INHBC;INHBE;INS;INS-IGF2;INSL3;INSL4;INSL5;INSL6;JAG1;JAG2;KITLG;KL;LACRT;LECT2;LEFTY1;LEFTY2;LEP;LHB;LIF;LRSAM1;LTA;LTB;LTBP1;LTBP2;LTBP3;LTBP4;MDK;MIA;MIF;MLN;MSTN;NAMPT;NDP;NENF;NGF;NMB;NODAL;NOV;NPFF;NPPA;NPPB;NPPC;NPY;NRG1;NRG2;NRG3;NRG4;NRTN;NTF3;NTF4;NTS;NUDT6;OGN;OSGIN1;OSM;OSTN;OXT;PDGFA;PDGFB;PDGFC;PDGFD;PDGFRA;PDGFRB;PDGFRL;PDYN;PENK;PF4;PF4V1;PGF;PLAU;PMCH;PNOC;POMC;PPBP;PPY;PRL;PRLH;PROK1;PROK2;PSPN;PTH;PTH2;PTHLH;PTN;PYY;QRFP;RABEP1;RABEP2;REG1A;RETN;RETNLB;RLN1;RLN2;RLN3;RNASE2;S100A6;SAA1;SAA2;SBDS;SCG2;SCGB3A1;SCT;SECTM1;SEMA3A;SEMA3B;SEMA3C;SEMA3D;SEMA3E;SEMA3F;SEMA3G;SEMA4A;SEMA4B;SEMA4C;SEMA4D;SEMA4F;SEMA4G;SEMA5A;SEMA5B;SEMA6A;SEMA6B;SEMA6C;SEMA6D;SEMA7A;SLIT1;SLIT2;SLURP1;SPP1;SST;STC1;STC2;TAC1;TDGF1;TG;TGFA;TGFB1;TGFB2;TGFB3;THPO;TNC;TNF;TNFRSF11B;TNFSF10;TNFSF11;TNFSF12;TNFSF13;TNFSF13B;TNFSF14;TNFSF15;TNFSF18;TNFSF4;TNFSF8;TNFSF9;TOR2A;TRH;TSHB;TSLP;TXLNA;TYMP;UCN;UCN2;UCN3;UTS2;VEGFA;VEGFB;VEGFC;VGF;VIP;XCL1;XCL2	428

Pathway	Gene name	The number of genes
Cytokine Receptors	ACVR1B;ACVR1C;ACVR2A;ACVR2B;ACVRL1;ADCYAP1R1;ADIPOR1;ADIPOR2;ADRB1;ADRB2;AGTR1;AGTR2;AMHR2;ANGPT1;ANGPT4;ANGPTL1;ANGPTL2;ANGPTL3;ANGPTL4;ANGPTL6;APLNR;AR;AVPR1A;AVPR1B;AVPR2;BMPR1A;BMPR1B;BMPR2;BRD8;C3AR1;C5AR1;CALCR;CALCRL;CCR1;CCR10;CCR3;CCR4;CCR5;CCR6;CCR7;CCR8;CCR9;CCRL2;CD40;CMKLR1;CNTFR;CRHR1;CRHR2;CRIM1;CRLF1;CRLF2;CRLF3;CSF1R;CSF2RA;CSF2RB;CSF3R;CX3CR1;CXCR3;CXCR4;CXCR5;CXCR6;CYSLTR1;CYSLTR2;EDNRA;EDNRB;EGFR;ENG;EPOR;ESR1;ESR2;ESRRA;ESRRB;ESRRG;FGFR1;FGFR2;FGFR3;FGFR4;FGFRL1;FLT1;FLT3;FLT4;FPR1;FPR2;FSHR;GALR2;GALR3;GCGR;GHR;GHRHR;GHSR;GIPR;GLP1R;GLP2R;GNRHR;GPR17;GPR32;GPR33;HNF4A;HNF4G;HTR3A;HTR3B;HTR3C;HTR3D;HTR3E;IFNAR1;IFNAR2;IFNGR1;IFNGR2;IGF1R;IGF2R;IL10RA;IL10RB;IL11RA;IL12RB1;IL12RB2;IL13RA1;IL13RA2;IL15RA;IL17RA;IL17RB;IL17RC;IL17RD;IL17RE;IL18R1;IL18RAP;IL1R1;IL1R2;IL1RAP;IL1RL1;IL1RL2;IL20RA;IL20RB;IL21R;IL22RA1;IL22RA2;IL23R;IL27RA;IL2RA;IL2RB;IL2RG;IL31RA;IL3RA;IL4R;IL5RA;IL6R;IL7R;IL9R;INSR;KDR;LEPR;LGR4;LGR5;LGR6;LHCGR;LIFR;LTB4R;LTB4R2;LTBR;MC1R;MC2R;MC3R;MC4R;MCHR1;MCHR2;MET;MLNR;MPL;MTNR1A;MTNR1B;NGFR;NMBR;NPR1;NPR3;NR0B1;NR0B2;NR1D1;NR1D2;NR1H2;NR1H3;NR1H4;NR1I2;NR1I3;NR2C1;NR2C2;NR2E1;NR2E3;NR2F1;NR2F2;NR2F6;NR3C1;NR3C2;NR4A1;NR4A2;NR4A3;NR5A1;NR5A2;NR6A1;NRP1;NRP2;OGFR;OPRD1;OPRK1;OPRL1;OPRM1;OSMR;OXTR;PGR;PGRMC2;PLAUR;PLXNA1;PLXNA2;PLXNA3;PLXNA4;PLXNB1;PLXNB2;PLXNB3;PLXNC1;PLXND1;PPARA;PPARD;PPARG;PRLHR;PRLR;PTAFR;PTGDR;PTGDS;PTGER1;PTGER2;PTGER3;PTGER4;PTGFR;PTH1R;PTH2R;RARA;RARB;RARG;ROBO1;ROBO2;ROBO3;RORA;RORB;RORC;RXFP1;RXFP2;RXFP3;RXRA;RXRB;RXRG;S1PR1;S1PR2;SCTR;SDC1;SDC2;SDC3;SDC4;SORT1;SSTR1;SSTR2;SSTR5;TACR1;TEK;TGFBR1;TGFBR2;TGFBR3;THRA;THRB;TIE1;TNFRSF10A;TNFRSF10B;TNFRSF10C;TNFRSF10D;TNFRSF11A;TNFRSF12A;TNFRSF13B;TNFRSF13C;TNFRSF14;TNFRSF17;TNFRSF18;TNFRSF19;TNFRSF1A;TNFRSF1B;TNFRSF21;TNFRSF25;TNFRSF4;TNFRSF6B;TNFRSF8;TNFRSF9;TRHR;TSHR;TUBB3;VDR;VIPR1;VIPR2;XCR1	294
Interferons	IFNA10;IFNA13;IFNA14;IFNA16;IFNA17;IFNA2;IFNA21;IFNA4;IFNA5;IFNA6;IFNA7;IFNA8;IFNB1;IFNE;IFNG;IFNK;IFNW1	17
Interferon Receptor	IFNAR2;IFNGR1;IFNGR2	3
Interleukins	IL11;IL12A;IL12B;IL13;IL15;IL16;IL17A;IL17B;IL17C;IL17D;IL17F;IL18;IL19;IL1A;IL1B;IL1F10;IL1RN;IL2;IL20;IL21;IL22;IL23A;IL24;IL25;IL26;IL27;IL3;IL31;IL32;IL33;IL34;IL4;IL5;IL6;IL6ST;IL7;IL9;TXLNA	38
Interleukins Receptor	IL10RA;IL10RB;IL11RA;IL12RB1;IL12RB2;IL13RA1;IL13RA2;IL15RA;IL17RA;IL17RB;IL17RC;IL17RD;IL17RE;IL18R1;IL18RAP;IL1R1;IL1R2;IL1RAP;IL1RL1;IL1RL2;IL20RA;IL20RB;IL21R;IL22RA1;IL22RA2;IL23R;IL27RA;IL2RA;IL2RB;IL2RG;IL31RA;IL3RA;IL4R;IL5RA;IL6R;IL7R;IL9R	37

**Pathway**	**Gene name**	**The number of genes**
Natural Killer Cell Cytotoxicity	HLA-A;HLA-B;HLA-C;HLA-E;HLA-G;KIR3DL1;KIR3DL2;KIR2DL1;KIR2DL3;KIR2DL4;KLRC1;KLRC2;KLRC3;KLRD1;PTPN6;PTPN11;ICAM1;ICAM2;ITGAL;ITGB2;PTK2B;VAV3;VAV1;VAV2;RAC1;RAC2;RAC3;PAK1;MAP2K1;MAP2K2;MAPK1;MAPK3;TNF;CSF2;IFNG;NCR2;TYROBP;LCK;FCGR3A;FCGR3B;NCR1;NCR3;FCER1G;CD247;ZAP70;SYK;LCP2;LAT;PLCG1;PLCG2;SH3BP2;PIK3CA;PIK3CB;PIK3CD;PIK3CG;PIK3R5;PIK3R1;PIK3R2;PIK3R3;FYN;SHC2;SHC4;SHC3;SHC1;GRB2;SOS1;SOS2;HRAS;KRAS;NRAS;ARAF;BRAF;RAF1;MICA;MICB;ULBP3;ULBP2;ULBP1;KLRK1;HCST;CD48;CD244;PPP3CA;PPP3CB;PPP3CC;PPP3R1;PPP3R2;CHP2;NFAT5;NFATC1;NFATC2;NFATC3;NFATC4;PRKCA;PRKCB;PRKCG;SH2D1B;SH2D1A;IFNGR1;IFNGR2;IFNA1;IFNA2;IFNA4;IFNA5;IFNA6;IFNA7;IFNA8;IFNA10;IFNA13;IFNA14;IFNA16;IFNA17;IFNA21;IFNB1;IFNAR1;IFNAR2;TNFSF10;TNFRSF10D;TNFRSF10C;TNFRSF10B;TNFRSF10A;FASLG;FAS;GZMB;PRF1;CASP3;BID	127
TCR signaling Pathway	CD3D;CD3E;CD3G;CD247;CD4;CD8A;CD8B;PTPRC;LCK;FYN;ZAP70;LCP2;LAT;ITK;TEC;NCK1;NCK2;VAV3;VAV1;VAV2;GRAP2;GRB2;PAK1;PAK2;PAK3;PAK4;PAK6;RHOA;CDC42;PPP3CA;PPP3CB;PPP3CC;PPP3R1;PPP3R2;CHP2;NFAT5;NFATC1;NFATC2;NFATC3;NFATC4;SOS1;SOS2;HRAS;KRAS;NRAS;FOS;JUN;CARD11;BCL10;MALT1;CHUK;IKBKB;IKBKG;NFKB1;RELA;NFKBIA;NFKBIB;NFKBIE;CD28;ICOS;CD40LG;PIK3R5;PIK3R1;PIK3R2;PIK3R3;PIK3CA;PIK3CB;PIK3CD;PIK3CG;AKT3;AKT1;AKT2;MAP3K8;MAP3K14;PDCD1;CTLA4;PTPN6;CBLC;CBL;CBLB;IL2;IL4;IL5;IL10;IFNG;CSF2;TNF;CDK4;RASGRP1;PDK1;PLCG1;PRKCQ	92
TGFb Family Member	BMP1;BMP10;BMP15;BMP2;BMP3;BMP4;BMP5;BMP6;BMP7;BMP8A;BMP8B;GDF1;GDF10;GDF11;GDF15;GDF2;GDF3;GDF5;GDF6;GDF7;GDF9;GDNF;INHA;INHBA;INHBB;INHBC;INHBE;LEFTY1;LEFTY2;NODAL;TGFB1;TGFB2;TGFB3	33
TGFb Family Member Receptor	ACVR1B;ACVR1C;ACVR2A;ACVR2B;ACVRL1;AMHR2;BMPR1A;BMPR1B;BMPR2;TGFBR1;TGFBR2;TGFBR3	12
TNF Family Members	TNFRSF11B;TNFSF10;TNFSF11;TNFSF12;TNFSF13;TNFSF13B;TNFSF14;TNFSF15;TNFSF18;TNFSF4;TNFSF8;TNFSF9	12
TNF Family Members Receptors	TNFRSF10B;TNFRSF10C;TNFRSF10D;TNFRSF11A;TNFRSF12A;TNFRSF13B;TNFRSF13C;TNFRSF14;TNFRSF17;TNFRSF18;TNFRSF19;TNFRSF1A;TNFRSF1B;TNFRSF21;TNFRSF25;TNFRSF4;TNFRSF6B;TNFRSF8;TNFRSF9	19

Table S3. Sensitivity analysis of partial correlation, FDR, and p-value thresholds on the number of robust snoRNA–immune gene pairs in breast cancer (BRCA)

parameter	threshold	n_robust_pairs	jaccard	top100_retention
partial_cor	0	9976	1	0.96
partial_cor	0.02	9976	1	0.96
partial_cor	0.05	9976	1	0.96
partial_cor	0.08	9976	1	0.96
partial_cor	0.1	9976	1	0.96
partial_cor	0.12	9976	1	0.96
partial_cor	0.15	9976	1	0.96
partial_cor	0.2	9976	1	0.96
partial_cor	0.25	9976	1	0.96
partial_cor	0.3	2777	0.27836808340016	0.96
FDR	0.01	9970	0.999398556535686	0.92
FDR	0.05	9971	0.999498797113071	0.92
FDR	0.1	9971	0.999498797113071	0.92
FDR	0.15	9972	0.999599037690457	0.92
FDR	0.2	9975	0.999899759422614	0.95
FDR	0.25	9976	1	0.96
FDR	0.3	9976	1	0.96
FDR	0.4	9976	1	0.96
FDR	0.5	9976	1	0.96
p_value	0.001	9976	1	0.96
p_value	0.005	9976	1	0.96
p_value	0.01	9976	1	0.96
p_value	0.02	9976	1	0.96
p_value	0.05	9976	1	0.96
p_value	0.1	9976	1	0.96
p_value	0.15	9976	1	0.96
p_value	0.2	9976	1	0.96
p_value	0.3	9976	1	0.96

Table S4. The top 500 SnoRNA-pathway pairs in different cancer types.

SnoRNA-name	Pathway	Number	Cancer types
SCARNA3_chr1_175937532_175937676	APC	19	ACC, BLCA, BRCA, CHOL, HNSC, KIRC, KIRP, LUSC, MESO, OV, PCPG, PRAD, SARC, SKCM, TGCT, THCA, THYM, UCS, UVM
SNORD83A_chr22_39711217_39711312	APC	19	ACC, BLCA, CESC, CHOL, COAD, DLBC, ESCA, HNSC, KIRP, LUSC, OV, PAAD, PCPG, SKCM, TGCT, THCA, THYM, UCEC, UVM
SNORD116-25_chr15_25342808_25342902	APC	17	ACC, BLCA, BRCA, CESC, DLBC, HNSC, KICH, LGG, OV, PRAD, SARC, SKCM, STAD, TGCT, THCA, UCS, UVM
ACA43_chr9_139620555_139620691	APC	17	ACC, BLCA, CESC, COAD, HNSC, KIRC, KIRP, LGG, LUSC, OV, PCPG, READ, SARC, SKCM, THCA, THYM, UVM
SNORD113-6_chr14_101405892_101405968	APC	17	ACC, BLCA, CESC, CHOL, COAD, KICH, KIRP, LIHC, LUAD, LUSC, OV, READ, TGCT, THCA, THYM, UCS, UVM
SNORA58_chr1_154232203_154232338	APC	17	BLCA, CESC, CHOL, COAD, DLBC, ESCA, HNSC, KIRP, LIHC, LUSC, OV, PCPG, SARC, SKCM, STAD, THCA, UCEC
SNORD114-13_chr14_101436215_101436289	APC	16	ACC, BLCA, CESC, COAD, ESCA, KICH, KIRP, LIHC, LUAD, LUSC, OV, STAD, THCA, THYM, UCS, UVM
SNORD18B_chr15_66794358_66794429	APC	16	ACC, BLCA, CESC, KICH, KIRC, LGG, LIHC, LUAD, LUSC, OV, PCPG, READ, SARC, STAD, THCA, UCS
SNORA3_chr16_2846409_2846533	APC	16	ACC, BRCA, CESC, COAD, KICH, KIRC, KIRP, LGG, LUSC, MESO, OV, SARC, SKCM, THCA, THYM, UCEC
snoU13_chr2_178210586_178210689	APC	16	ACC, BLCA, CHOL, COAD, ESCA, KIRP, LGG, LUAD, MESO, OV, SKCM, STAD, THCA, THYM, UCEC, UCS
SNORD99_chr1_28905254_28905334	APC	16	BRCA, CHOL, COAD, KICH, KIRC, KIRP, LGG, LIHC, LUSC, MESO, OV, SARC, THCA, THYM, UCEC, UVM
SNORD38A_chr1_45243513_45243584	Antimicrobials	16	CESC, CHOL, ESCA, HNSC, KIRP, LIHC, LUAD, LUSC, OV, PAAD, PCPG, PRAD, TGCT, THYM, UCEC, UCS
SNORD114-17_chr14_101441142_101441217	APC	15	ACC, BLCA, CESC, COAD, KICH, KIRP, LUAD, OV, PRAD, STAD, THCA, THYM, UCEC, UCS, UVM
SNORD114-16_chr14_101439931_101440001	APC	15	ACC, BLCA, KICH, KIRP, LIHC, LUAD, OV, PRAD, SKCM, STAD, THCA, THYM, UCEC, UCS, UVM
ACA31_chr13_45911614_45911744	APC	15	ACC, BLCA, CESC, COAD, KIRC, KIRP, LGG, LUSC, MESO, OV, SARC, SKCM, STAD, THCA, THYM
SNORD114-23_chr14_101450212_101450284	APC	15	ACC, BLCA, CESC, KICH, LIHC, LUAD, LUSC, OV, SKCM, STAD, TGCT, THCA, THYM, UCS, UVM
SNORD125_chr22_29729151_29729247	APC	15	ACC, BLCA, BRCA, HNSC, KIRC, KIRP, LGG, LUAD, MESO, OV, SARC, SKCM, STAD, THYM, UVM

SnoRNA-name	Pathway	Number	Cancer types
SNORD58B_chr18_47018033_47018099	APC	15	BLCA, COAD, DLBC, HNSC, LGG, LUSC, MESO, OV, PAAD, PRAD, SARC, SKCM, THYM, UCEC, UVM
SNORD13_chr8_33370991_33371096	APC	15	BRCA, CHOL, COAD, DLBC, KICH, KIRC, KIRP, LGG, MESO, READ, SARC, SKCM, THCA, UCEC, UCS
RNU3P3_chr14_85738276_85738405	APC	15	BRCA, CHOL, COAD, HNSC, KICH, KIRP, LGG, LIHC, LUAD, OV, PRAD, SARC, THYM, UCEC, UVM
U3_chr9_90989184_90989274	APC	15	BRCA, CHOL, COAD, HNSC, KICH, KIRP, LGG, LIHC, LUAD, LUSC, MESO, SARC, THYM, UCEC, UVM
SNORD12B_chr20_47896855_47896946	Antimicrobials	14	ACC, CESC, CHOL, LUAD, LUSC, OV, PAAD, PCPG, PRAD, READ, STAD, TGCT, UCEC, UVM
SNORD58A_chr18_47017652_47017717	Antimicrobials	14	ACC, BRCA, CHOL, HNSC, KIRP, LIHC, MESO, PAAD, READ, TGCT, THCA, THYM, UCEC, UCS
SNORD58C_chr18_47015613_47015678	Antimicrobials	14	ACC, BRCA, ESCA, HNSC, KIRP, LIHC, LUAD, MESO, PRAD, STAD, TGCT, THCA, UCEC, UCS
SNORA56_chrX_154003272_154003401	APC	14	ACC, BLCA, BRCA, CESC, COAD, KIRC, KIRP, LUSC, OV, PAAD, SKCM, THCA, THYM, UVM
U28_chr11_62622092_62622167	APC	14	ACC, BRCA, KIRC, KIRP, LIHC, MESO, OV, PRAD, READ, SARC, SKCM, TGCT, THCA, THYM
U31_chr11_62620796_62620867	APC	14	ACC, BRCA, CHOL, HNSC, KICH, LGG, LUAD, LUSC, MESO, PAAD, SARC, STAD, THCA, UCEC
SNORD114-11_chr14_101434447_101434522	APC	14	ACC, BLCA, DLBC, ESCA, KICH, KIRP, LUAD, OV, SKCM, STAD, THCA, THYM, UCS, UVM
SCARNA5_chr2_234184371_234184649	APC	14	ACC, BLCA, COAD, DLBC, HNSC, KIRC, KIRP, LUAD, LUSC, MESO, SKCM, STAD, THCA, UVM
SNORD114-20_chr14_101447340_101447412	APC	14	ACC, BLCA, CESC, COAD, DLBC, HNSC, LIHC, OV, TGCT, THCA, THYM, UCEC, UCS, UVM
SNORD114-5_chr14_101421706_101421776	APC	14	ACC, BLCA, CESC, COAD, KICH, KIRP, LIHC, OV, SKCM, STAD, THCA, THYM, UCEC, UCS
SNORD36_chr13_23377284_23377363	Antimicrobials	14	ACC, BLCA, CESC, CHOL, DLBC, ESCA, HNSC, LIHC, LUAD, OV, TGCT, THYM, UCEC, UCS
SCARNA4_chr1_155895748_155895877	APC	14	ACC, BLCA, CESC, COAD, KIRC, KIRP, LGG, LUSC, OV, SKCM, TGCT, THCA, UCEC, UCS
SNORD69_chr3_52726751_52726828	APC	14	ACC, BLCA, BRCA, CESC, CHOL, COAD, DLBC, HNSC, LGG, LUSC, MESO, OV, THYM, UVM
SNORD114-12_chr14_101435284_101435359	APC	14	ACC, BLCA, CESC, CHOL, KICH, KIRP, LIHC, LUAD, LUSC, STAD, THCA, THYM, UCEC, UVM
SNORD70_chr2_203141153_203141241	Antimicrobials	14	ACC, BRCA, CHOL, ESCA, HNSC, KIRP, LGG, LUAD, LUSC, PAAD, STAD, TGCT, THCA, UVM
snoU13_chr4_17530560_17530663	APC	14	ACC, CESC, CHOL, ESCA, KICH, LGG, LIHC, OV, PAAD, PCPG, SKCM, STAD, THCA, UCEC

SnoRNA-name	Pathway	Number	Cancer types
ACA58_chr3_131197940_131198077	APC	14	ACC, BLCA, CESC, CHOL, COAD, LIHC, LUAD, LUSC, MESO, OV, SARC, SKCM, THCA, UVM
SNORD16_chr15_66795148_66795249	Antimicrobials	14	BLCA, COAD, DLBC, HNSC, KIRP, LUAD, LUSC, PAAD, PCPG, PRAD, STAD, TGCT, UCEC, UCS
SNORD123_chr5_9548947_9549017	APC	14	BLCA, CESC, COAD, HNSC, KICH, LGG, LUAD, MESO, PRAD, SARC, THCA, THYM, UCEC, UCS
ACA62_chr17_62223698_62223831	APC	14	BLCA, CESC, COAD, DLBC, HNSC, KICH, KIRC, KIRP, LIHC, LUSC, MESO, OV, PRAD, SKCM
SNORA74A_chr5_138614468_138614668	APC	14	BLCA, CESC, COAD, DLBC, HNSC, KIRC, KIRP, LUAD, LUSC, OV, SARC, SKCM, THCA, UCS
U76_chr1_173835772_173835852	APC	14	BLCA, CHOL, COAD, DLBC, HNSC, KIRP, LGG, OV, PAAD, PRAD, READ, SKCM, TGCT, UCEC
U3_chr2_114763018_114763232	APC	14	BLCA, BRCA, CHOL, COAD, HNSC, KICH, KIRP, LGG, MESO, OV, SARC, THYM, UCEC, UVM
SNORA81_chr3_186504463_186504641	APC	14	BLCA, BRCA, CESC, COAD, KIRC, KIRP, LGG, LUAD, LUSC, MESO, OV, PCPG, SKCM, UVM
SNORA6_chr3_39449880_39450030	APC	14	BLCA, CESC, COAD, DLBC, KIRC, KIRP, LUSC, OV, SARC, SKCM, THCA, UCEC, UCS, UVM
SNORA66_chr1_93306275_93306408	APC	14	BLCA, BRCA, CESC, CHOL, COAD, DLBC, ESCA, KIRC, KIRP, LUSC, OV, PCPG, READ, SKCM
SNORA70_chr8_4985801_4985934	APC	14	BLCA, CESC, CHOL, COAD, DLBC, ESCA, KIRC, KIRP, LUAD, LUSC, OV, SARC, SKCM, THCA
SNORA70B_chr2_61644378_61644513	APC	14	BLCA, COAD, ESCA, KICH, KIRC, KIRP, LUSC, MESO, OV, PAAD, PRAD, SKCM, UCEC, UVM
ACA44_chr1_28906892_28907024	APC	14	BRCA, CESC, CHOL, COAD, KICH, KIRC, KIRP, LGG, LIHC, MESO, OV, THCA, THYM, UVM
SNORA73B_chr1_28835069_28835274	APC	14	COAD, DLBC, ESCA, KIRC, KIRP, LIHC, LUSC, MESO, OV, PCPG, SARC, SKCM, THCA, THYM
SNORD15B_chr11_75115464_75115610	Antimicrobials	13	ACC, BLCA, BRCA, CESC, ESCA, HNSC, LUAD, LUSC, MESO, PRAD, TGCT, THYM, UCEC
U44_chr1_173835103_173835166	Antimicrobials	13	ACC, BRCA, CHOL, COAD, ESCA, LUSC, OV, PAAD, READ, TGCT, THCA, THYM, UCS
SNORD116-16_chr15_25327913_25328007	APC	13	ACC, BLCA, BRCA, DLBC, LUSC, MESO, OV, SARC, SKCM, STAD, THCA, THYM, UCEC
U26_chr11_62622763_62622838	Antimicrobials	13	ACC, CESC, DLBC, HNSC, KIRP, LGG, LUAD, LUSC, PCPG, PRAD, SKCM, THYM, UCEC
SNORD114-10_chr14_101433388_101433460	APC	13	ACC, BLCA, CESC, COAD, DLBC, KICH, KIRP, LIHC, OV, STAD, THCA, THYM, UCS
SNORD114-22_chr14_101449262_101449334	APC	13	ACC, BLCA, KICH, KIRP, LUSC, OV, READ, SKCM, STAD, THCA, THYM, UCEC, UVM
SNORD114-25_chr14_101452393_101452465	APC	13	ACC, BLCA, CESC, KIRP, LUAD, LUSC, OV, STAD, THCA, THYM, UCEC, UCS, UVM
SNORD116-6_chr15_25310171_25310269	APC	13	ACC, BLCA, BRCA, COAD, DLBC, ESCA, LUAD, LUSC, MESO, OV, SKCM, THCA, THYM
SNORD113-3_chr14_101396255_101396328	APC	13	ACC, BLCA, CESC, COAD, KICH, KIRP, LUAD, LUSC, SKCM, THCA, THYM, UCS, UVM

SnoRNA-name	Pathway	Number	Cancer types
SNORD116-29_chr15_25351666_25351751	APC	13	ACC, BLCA, BRCA, HNSC, KICH, KIRC, LUSC, MESO, PRAD, SKCM, TGCT, THCA, UCEC
SNORD114-26_chr14_101453382_101453454	APC	13	ACC, BLCA, CESC, KICH, KIRP, LIHC, LUSC, OV, STAD, THCA, THYM, UCS, UVM
SNORD14C_chr11_122930043_122930130	Antimicrobials	13	ACC, BLCA, CESC, COAD, DLBC, ESCA, LUAD, LUSC, PAAD, PRAD, TGCT, UCEC, UCS
SNORD116-1_chr15_25296622_25296719	APC	13	ACC, BLCA, CHOL, COAD, DLBC, LGG, LUAD, LUSC, OV, SKCM, THCA, THYM, UCEC
SNORD116-8_chr15_25315577_25315674	APC	13	ACC, BLCA, BRCA, HNSC, LUAD, LUSC, OV, PCPG, SKCM, STAD, THCA, THYM, UVM
SNORD45A_chr1_76253573_76253657	APC	13	ACC, CHOL, KICH, KIRC, KIRP, LGG, LUAD, LUSC, MESO, SARC, SKCM, STAD, UCEC
SNORD14E_chr11_122928785_122928869	APC	13	ACC, BLCA, BRCA, ESCA, KICH, KIRC, KIRP, LGG, LUSC, OV, THCA, UCS, UVM
ACA9_chr7_45024976_45025109	APC	13	ACC, CESC, COAD, KIRC, KIRP, LUAD, LUSC, MESO, OV, READ, SARC, SKCM, TGCT
SNORD114-1_chr14_101416169_101416241	APC	13	ACC, BLCA, COAD, KICH, KIRP, LIHC, OV, SKCM, THCA, THYM, UCEC, UCS, UVM
SNORA46_chr16_58582402_58582537	APC	13	ACC, BLCA, BRCA, CESC, COAD, KIRC, KIRP, LGG, LIHC, LUAD, MESO, OV, SARC
SNORA1_chr8_56815282_56815414	APC	13	ACC, BLCA, BRCA, COAD, HNSC, KIRC, KIRP, LIHC, LUSC, MESO, OV, SARC, SKCM
SNORA64_chr16_2012973_2013107	APC	13	ACC, BLCA, CESC, COAD, HNSC, KIRC, KIRP, LGG, LIHC, OV, SARC, SKCM, THCA
U49A_chr17_16343349_16343420	Antimicrobials	13	BLCA, BRCA, CESC, CHOL, DLBC, ESCA, KIRP, LUAD, PAAD, PRAD, THCA, UCEC, UCS
SNORD114-21_chr14_101448311_101448383	APC	13	BLCA, CESC, KICH, KIRP, LIHC, LUAD, OV, SKCM, THCA, THYM, UCEC, UCS, UVM
SNORD41_chr19_12817262_12817332	Antimicrobials	13	BLCA, CHOL, COAD, ESCA, HNSC, KIRP, LUAD, OV, PAAD, PCPG, PRAD, TGCT, UCEC
SNORA69_chrX_118921315_118921447	APC	13	BLCA, CESC, COAD, KIRC, KIRP, LGG, LIHC, LUAD, LUSC, MESO, OV, SARC, SKCM
U85_chr12_6619387_6619717	APC	13	BLCA, CESC, COAD, ESCA, KIRC, KIRP, LUSC, MESO, SKCM, THYM, UCEC, UCS, UVM
U79_chr1_173834485_173834570	APC	13	BLCA, CHOL, COAD, DLBC, KIRC, KIRP, LGG, LUSC, OV, PRAD, SKCM, THCA, UCEC
SNORD3C_chr17_19092978_19093558	APC	13	BLCA, BRCA, KICH, KIRC, KIRP, LGG, LUSC, MESO, OV, PRAD, SKCM, UCS, UVM
ACA16_chr1_28907431_28907565	APC	13	BLCA, DLBC, KICH, KIRC, KIRP, LIHC, LUAD, LUSC, OV, SARC, THCA, THYM, UCEC
U3_chr8_124192551_124192765	APC	13	BLCA, BRCA, CHOL, COAD, HNSC, KICH, KIRP, LIHC, OV, SARC, THYM, UCEC, UVM
SNORA80_chr21_33749495_33749631	APC	13	BLCA, CESC, COAD, DLBC, ESCA, HNSC, LUSC, OV, PRAD, READ, SKCM, THCA, UVM
SNORA7_chrX_15734331_15734469	APC	13	BLCA, BRCA, KICH, KIRC, KIRP, LGG, LIHC, OV, PRAD, SARC, THCA, THYM, UVM
SNORD42A_chr17_27050447_27050510	Antimicrobials	13	BRCA, CHOL, HNSC, KIRP, LIHC, LUAD, LUSC, PAAD, SARC, TGCT, THYM, UCEC, UCS
SNORA48_chr17_7478030_7478165	Antimicrobials	13	BRCA, CESC, COAD, DLBC, ESCA, HNSC, LUAD, MESO, PAAD, PRAD, STAD, THYM, UCEC

SnoRNA-name	Pathway	Number	Cancer types
SNORD111_chr16_70571907_70572001	APC	13	BRCA, CESC, COAD, DLBC, HNSC, KIRC, LIHC, LUSC, OV, PRAD, SARC, UCEC, UCS
SNORA64_chr16_2012973_2013107	Antimicrobials	13	BRCA, CHOL, DLBC, ESCA, LUAD, LUSC, PAAD, PCPG, PRAD, READ, STAD, TGCT, UCEC
U17a_chr1_28833876_28834083	APC	13	BRCA, CESC, COAD, DLBC, KIRC, KIRP, LUSC, OV, PCPG, SARC, THCA, THYM, UCEC
U30_chr11_62621134_62621204	Antimicrobials	13	CESC, COAD, DLBC, ESCA, KIRP, LUAD, PCPG, PRAD, READ, SKCM, TGCT, THCA, UCEC
SNORD105_chr19_10218326_10218411	Antimicrobials	13	CESC, CHOL, DLBC, KIRP, LUSC, OV, PAAD, PCPG, PRAD, READ, TGCT, THCA, UCEC
SNORD70_chr2_203142831_203142915	APC	13	CESC, COAD, DLBC, KICH, LIHC, MESO, OV, PCPG, PRAD, SARC, SKCM, UCEC, UCS
ACA24_chr4_119200344_119200475	APC	13	CHOL, COAD, DLBC, HNSC, KIRP, LGG, OV, PCPG, SARC, SKCM, THYM, UCEC, UCS
SNORA71B_chr20_37053731_37054002	Antimicrobials	13	CHOL, ESCA, HNSC, LIHC, LUAD, LUSC, MESO, PAAD, STAD, TGCT, THCA, UCEC, UCS
mgU2-25-61_chr1_109642814_109643234	Antimicrobials	13	CHOL, COAD, DLBC, ESCA, HNSC, KIRP, LUAD, MESO, PRAD, READ, TGCT, THCA, UCEC
hTR_chr3_169482397_169482945	Antimicrobials	13	CHOL, DLBC, ESCA, HNSC, KIRP, LUAD, PRAD, READ, STAD, TGCT, THCA, UCEC, UCS
SNORD33_chr19_49993872_49993956	APC	12	ACC, BLCA, COAD, KIRC, LGG, LIHC, MESO, PRAD, TGCT, THCA, UCEC, UVM
SNORD46_chr7_132437783_132437886	APC	12	ACC, BLCA, COAD, KIRC, KIRP, LGG, LUSC, MESO, OV, READ, THYM, UVM
SNORD36A_chr9_136217310_136217383	Antimicrobials	12	ACC, BRCA, CESC, DLBC, ESCA, KIRP, LUAD, LUSC, PAAD, TGCT, THYM, UCEC
HBII-85-22_chr15_25335068_25335162	APC	12	ACC, BLCA, BRCA, CESC, DLBC, LUSC, OV, SARC, SKCM, STAD, THCA, UCEC
U29_chr11_62621375_62621440	Antimicrobials	12	ACC, BLCA, BRCA, CHOL, DLBC, KIRP, PAAD, PCPG, SKCM, TGCT, THCA, UCEC
SNORD113-5_chr14_101404523_101404601	APC	12	ACC, CESC, KICH, LGG, LIHC, LUAD, OV, PCPG, STAD, THCA, UCS, UVM
SNORD114-4_chr14_101420710_101420785	APC	12	ACC, BLCA, COAD, DLBC, KICH, LGG, OV, SKCM, STAD, THCA, THYM, UCS
HBII-85-20_chr15_25332807_25332901	APC	12	ACC, BLCA, BRCA, DLBC, HNSC, LUSC, MESO, OV, SKCM, THCA, THYM, UCEC
SNORD115-5_chr15_25423884_25423966	APC	12	ACC, BLCA, BRCA, COAD, KIRP, LGG, LIHC, MESO, PRAD, SKCM, THCA, UVM
HBII-436_chr15_25227140_25227215	APC	12	ACC, BLCA, HNSC, KIRC, LUAD, LUSC, OV, PCPG, THCA, UCEC, UCS, UVM
HBII-438A_chr15_25287120_25287187	APC	12	ACC, BLCA, BRCA, COAD, HNSC, KIRP, LGG, OV, SKCM, TGCT, UCEC, UCS
SNORD102_chr13_27829200_27829272	Antimicrobials	12	ACC, COAD, DLBC, HNSC, KIRP, OV, PAAD, PCPG, SKCM, TGCT, THCA, THYM
SNORD114-14_chr14_101438439_101438514	APC	12	ACC, CESC, COAD, KICH, KIRP, MESO, OV, THCA, THYM, UCEC, UCS, UVM
SNORD83B_chr22_39709823_39709916	APC	12	ACC, BLCA, CESC, DLBC, ESCA, HNSC, KIRC, KIRP, LUSC, MESO, SKCM, THCA

SnoRNA-name	Pathway	Number	Cancer types
U80_chr1_173833966_173834044	Antimicrobials	12	ACC, BRCA, COAD, DLBC, LGG, LUAD, LUSC, OV, PAAD, PRAD, THCA, THYM
SNORD53_chr2_29149932_29150010	APC	12	ACC, CHOL, COAD, DLBC, ESCA, HNSC, LIHC, SKCM, TGCT, THYM, UCEC, UCS
SNORD94_chr2_86362992_86363129	Antimicrobials	12	ACC, BLCA, BRCA, CHOL, DLBC, KIRP, LUAD, LUSC, PCPG, READ, TGCT, THCA
SNORD93_chr7_22896231_22896305	APC	12	ACC, BLCA, BRCA, COAD, HNSC, KIRP, LGG, LUAD, PAAD, SARC, THCA, UVM
SNORA55_chr1_40033045_40033182	APC	12	ACC, BLCA, CESC, COAD, ESCA, HNSC, KIRC, LUAD, MESO, OV, THCA, UCS
SNORA65_chr9_130210779_130210916	APC	12	ACC, ESCA, KIRC, KIRP, LGG, LIHC, MESO, OV, SARC, SKCM, THYM, UCEC
SNORA71D_chr20_37062504_37062642	Antimicrobials	12	ACC, BLCA, BRCA, CHOL, DLBC, ESCA, HNSC, LUAD, MESO, STAD, THCA, UCEC
SNORA8_chr11_93465526_93465665	APC	12	ACC, CESC, COAD, DLBC, KICH, KIRC, KIRP, LGG, MESO, SARC, UCEC, UVM
SNORA71B_chr20_37053731_37054002	APC	12	ACC, BLCA, CESC, COAD, DLBC, KIRC, KIRP, LGG, OV, PRAD, SKCM, THYM
SNORD14B_chr11_17097324_17097415	Antimicrobials	12	BLCA, CESC, COAD, DLBC, ESCA, HNSC, LUAD, PRAD, READ, TGCT, THYM, UCS
SNORA36B_chr1_220373887_220374018	APC	12	BLCA, BRCA, COAD, KICH, KIRP, LGG, LIHC, LUSC, OV, READ, THCA, UCS
SNORD119_chr20_2443604_2443686	Antimicrobials	12	BLCA, CESC, CHOL, KIRP, LUAD, LUSC, OV, PCPG, STAD, TGCT, THCA, UCEC
SNORD46_chr1_45242162_45242265	APC	12	BLCA, CESC, COAD, KIRC, KIRP, LGG, LUSC, OV, SKCM, THYM, UCEC, UCS
SNORD5_chr11_93466393_93466466	APC	12	BLCA, BRCA, COAD, KICH, KIRC, KIRP, OV, SARC, SKCM, THYM, UCS, UVM
SNORD92_chr2_29136527_29136616	APC	12	BLCA, CESC, COAD, ESCA, HNSC, KIRP, LIHC, LUSC, SARC, SKCM, THCA, UCS
SNORA33_chr6_133138357_133138490	APC	12	BLCA, COAD, KICH, KIRC, KIRP, LGG, OV, SARC, SKCM, THCA, THYM, UCEC
SNORD3B-2_chr17_18966659_18967449	APC	12	BLCA, BRCA, KICH, KIRC, KIRP, LGG, LUAD, MESO, PRAD, SARC, SKCM, UVM
SCARNA1_chr1_28160911_28161077	APC	12	BLCA, COAD, KICH, KIRC, LIHC, LUAD, LUSC, OV, READ, UCEC, UCS, UVM
snR38A_chr17_74557714_74557786	APC	12	BLCA, CESC, COAD, ESCA, KIRP, LIHC, LUAD, LUSC, MESO, SKCM, THCA, UVM
SNORD42B_chr17_27047567_27047634	Antimicrobials	12	BRCA, CHOL, DLBC, KIRP, LUAD, LUSC, PAAD, PCPG, PRAD, SKCM, THYM, UCS
SNORD91A_chr17_2233474_2233664	Antimicrobials	12	BRCA, CESC, CHOL, COAD, DLBC, HNSC, KIRP, OV, PAAD, PRAD, TGCT, UCEC
SNORD89_chr2_101889397_101889511	APC	12	BRCA, COAD, KIRP, LGG, LUAD, MESO, OV, PCPG, SARC, SKCM, THCA, UCEC
SNORA28_chr14_103804185_103804311	APC	12	BRCA, COAD, KICH, KIRP, LGG, LUSC, OV, SARC, SKCM, THYM, UCEC, UVM
14q(0)_chr14_101364256_101364333	APC	12	CESC, HNSC, KIRP, LIHC, LUSC, MESO, OV, THCA, THYM, UCEC, UCS, UVM
SNORA70_chrX_153628621_153628756	APC	12	CESC, CHOL, COAD, KICH, KIRC, KIRP, LIHC, OV, THCA, UCEC, UCS, UVM
SNORD88C_chr19_51305581_51305678	Antimicrobials	12	CESC, CHOL, DLBC, KIRP, LUAD, LUSC, PRAD, SARC, STAD, TGCT, THCA, UCEC

SnoRNA-name	Pathway	Number	Cancer types
U56_chr20_2637269_2637340	Antimicrobials	12	CHOL, HNSC, KIRP, LUSC, OV, PAAD, PCPG, PRAD, STAD, TGCT, THCA, UCEC
SNORA36A_chrX_153996802_153996934	APC	12	CHOL, COAD, HNSC, KIRP, LGG, OV, SARC, SKCM, STAD, UCEC, UCS, UVM
SNORD18C_chr15_66793588_66793656	Antimicrobials	12	COAD, DLBC, KIRP, LIHC, LUAD, MESO, PAAD, PRAD, TGCT, UCEC, UCS, UVM
SNORD45C_chr1_76252756_76252835	APC	12	KICH, KIRC, KIRP, LGG, LUAD, LUSC, MESO, SARC, SKCM, STAD, THCA, THYM
SCARNA14_chr15_66639543_66639680	APC	11	ACC, BRCA, CESC, KIRC, KIRP, LGG, LUSC, OV, SARC, SKCM, THYM
SNORD10_chr17_7480128_7480276	APC	11	ACC, BLCA, KICH, KIRC, KIRP, LGG, LIHC, LUSC, SARC, THYM, UCEC
SNORD38B_chr1_45244061_45244130	APC	11	ACC, BLCA, CESC, COAD, DLBC, LGG, MESO, OV, READ, SKCM, THYM
SNORD78_chr1_173834759_173834824	APC	11	ACC, BLCA, COAD, DLBC, HNSC, LGG, LUAD, MESO, SKCM, TGCT, UCS
SNORD9_chr14_21860309_21860412	APC	11	ACC, BLCA, CESC, COAD, LUAD, LUSC, OV, READ, STAD, UCEC, UCS
U27_chr11_62622483_62622555	APC	11	ACC, BLCA, COAD, DLBC, HNSC, LUAD, LUSC, SARC, SKCM, STAD, THCA
U62A_chr9_134361051_134361137	Antimicrobials	11	ACC, BLCA, CESC, DLBC, HNSC, LUSC, PAAD, PRAD, TGCT, THCA, UCEC
SNORD113-7_chr14_101407462_101407539	APC	11	ACC, BLCA, CESC, COAD, KICH, KIRP, OV, THCA, UCEC, UCS, UVM
SNORD113-8_chr14_101409787_101409861	APC	11	ACC, BLCA, CESC, COAD, KICH, KIRP, LUAD, THCA, THYM, UCS, UVM
SNORD114-24_chr14_101451113_101451185	APC	11	ACC, BLCA, CESC, COAD, KICH, LIHC, LUSC, OV, THCA, THYM, UCS
SNORD114-28_chr14_101455466_101455538	APC	11	ACC, BLCA, CESC, COAD, KIRP, LUSC, OV, THCA, THYM, UCS, UVM
SNORD114-3_chr14_101419685_101419760	APC	11	ACC, BLCA, CESC, CHOL, COAD, KIRP, OV, THCA, THYM, UCS, UVM
SNORD114-27_chr14_101454497_101454567	APC	11	ACC, CESC, DLBC, KICH, LUSC, OV, SKCM, THCA, THYM, UCS, UVM
SNORD115-44_chr15_25496005_25496087	APC	11	ACC, BRCA, CESC, KIRP, LGG, LIHC, LUAD, PAAD, SKCM, THCA, UCS
SNORD116-9_chr15_25318252_25318349	APC	11	ACC, BLCA, BRCA, COAD, DLBC, LGG, LUSC, SKCM, THCA, THYM, UCEC
SNORD103A_chr1_31408532_31408623	Antimicrobials	11	ACC, CESC, CHOL, COAD, HNSC, LGG, MESO, OV, PRAD, READ, THCA
SNORD114-6_chr14_101423502_101423574	APC	11	ACC, CESC, KICH, KIRP, LIHC, OV, SKCM, THCA, THYM, UCS, UVM
SNORD21_chr1_93302845_93302940	Antimicrobials	11	ACC, BLCA, CHOL, COAD, DLBC, KIRP, PAAD, PCPG, PRAD, TGCT, UCEC
SNORD67_chr11_46783938_46784049	Antimicrobials	11	ACC, BLCA, BRCA, CHOL, DLBC, HNSC, LUAD, LUSC, MESO, THYM, UCS
SNORD115-14_chr15_25440067_25440148	APC	11	ACC, BRCA, CHOL, KIRC, KIRP, LGG, LIHC, LUAD, OV, UCEC, UVM
SNORD116-5_chr15_25307478_25307575	APC	11	ACC, BLCA, BRCA, DLBC, LGG, OV, SKCM, THCA, THYM, UCEC, UVM
SNORA1_chr11_19612703_19612838	APC	11	ACC, BLCA, BRCA, COAD, DLBC, KIRC, KIRP, LIHC, MESO, OV, SARC

SnoRNA-name	Pathway	Number	Cancer types
SNORA5C_chr7_45144504_45144641	APC	11	ACC, CHOL, COAD, KIRC, LGG, LUAD, MESO, OV, SARC, SKCM, UCS
SNORA31_chr13_45910449_45910582	APC	11	ACC, BLCA, COAD, DLBC, KIRC, KIRP, OV, PAAD, SARC, SKCM, UCEC
SNORD114-19_chr14_101442813_101442888	APC	11	ACC, BLCA, CESC, KICH, LUSC, MESO, OV, SKCM, THCA, UCS, UVM
SNORD116-2_chr15_25299355_25299452	APC	11	ACC, BLCA, BRCA, DLBC, HNSC, LUAD, LUSC, MESO, OV, THCA, THYM
SNORD3A_chr17_19091328_19092027	Antimicrobials	11	ACC, CESC, CHOL, COAD, DLBC, LIHC, LUAD, PAAD, PRAD, READ, THCA
SNORD3B-2_chr17_18966659_18967449	Antimicrobials	11	ACC, CESC, CHOL, COAD, DLBC, HNSC, LIHC, OV, TGCT, THCA, UCEC
SNORD115-29_chr15_25468392_25468474	APC	11	ACC, BLCA, CHOL, COAD, LGG, LIHC, MESO, OV, THCA, UCEC, UVM
SNORD13_chr8_33370991_33371096	Antimicrobials	11	ACC, BLCA, CESC, ESCA, HNSC, LUSC, OV, PAAD, PCPG, PRAD, THYM
U3_chrX_70065931_70066145	APC	11	ACC, BLCA, ESCA, KICH, KIRP, LUSC, SKCM, THCA, THYM, UCEC, UCS
SCARNA17_chr18_47340392_47340813	APC	11	ACC, CESC, CHOL, COAD, KIRP, LGG, LUAD, MESO, SARC, THCA, UCEC
SCARNA18_chr18_47340392_47340813	APC	11	ACC, CESC, CHOL, COAD, KIRP, LGG, LUAD, MESO, SARC, THCA, UCEC
HBII-276_chr8_67834708_67834784	APC	11	ACC, BLCA, CESC, HNSC, KIRC, KIRP, LGG, LUAD, LUSC, THCA, UCEC
snR38C_chr17_74554871_74554951	APC	11	ACC, COAD, ESCA, KIRP, LIHC, OV, READ, SKCM, THCA, UCS, UVM
U3_chr14_68212084_68212191	APC	11	ACC, CHOL, COAD, HNSC, KIRP, LGG, LIHC, OV, SARC, THCA, UVM
SNORD70_chr2_203141153_203141241	APC	11	BLCA, CESC, COAD, DLBC, LIHC, MESO, PCPG, PRAD, SKCM, UCEC, UCS
U81_chr1_173833283_173833360	APC	11	BLCA, CESC, HNSC, LIHC, LUSC, MESO, OV, SKCM, THCA, THYM, UVM
SNORD114-9_chr14_101432365_101432437	APC	11	BLCA, COAD, KIRP, OV, PRAD, SKCM, STAD, THCA, THYM, UCS, UVM
SNORA1_chr11_93465169_93465299	APC	11	BLCA, BRCA, DLBC, KIRP, LIHC, MESO, OV, SARC, SKCM, THYM, UCS
SNORA23_chr11_9450312_9450501	Antimicrobials	11	BLCA, BRCA, CHOL, DLBC, HNSC, LIHC, LUAD, LUSC, PAAD, THYM, UCEC
SNORD15A_chr11_75111434_75111582	APC	11	BLCA, COAD, KIRC, KIRP, LGG, OV, PRAD, SKCM, TGCT, THCA, THYM
SNORD18_chr2_12170429_12170498	APC	11	BLCA, KICH, KIRC, KIRP, LUSC, OV, SKCM, STAD, THCA, THYM, UCS
ACA9_chr7_45024976_45025109	Antimicrobials	11	BLCA, DLBC, ESCA, HNSC, KICH, PAAD, PRAD, STAD, THCA, THYM, UCEC
SNORA24_chr3_128433414_128433548	APC	11	BLCA, COAD, DLBC, KIRC, KIRP, OV, PAAD, SARC, SKCM, THCA, THYM
SNORA14B_chr1_235291117_235291252	APC	11	BLCA, CESC, COAD, ESCA, KICH, KIRP, LUAD, LUSC, MESO, SKCM, UCEC
SNORD53_SNORD92_chr2_29150849_29150926	APC	11	BLCA, CESC, COAD, LGG, LUSC, PAAD, SKCM, TGCT, THYM, UCEC, UCS
SNORA19_chr10_120819522_120819650	APC	11	BLCA, HNSC, KIRC, KIRP, LUAD, LUSC, MESO, OV, PAAD, SARC, STAD

SnoRNA-name	Pathway	Number	Cancer types
SNORD118_chr17_8076770_8076906	Antimicrobials	11	BLCA, CHOL, COAD, ESCA, HNSC, LUSC, MESO, PAAD, PRAD, UCEC, UCS
SCARNA13_chr14_95999691_95999966	APC	11	BLCA, CESC, COAD, HNSC, LGG, LUAD, LUSC, MESO, THCA, UCEC, UVM
SCARNA20_chr17_58308876_58309007	APC	11	BLCA, CESC, CHOL, COAD, LUAD, OV, PAAD, READ, SKCM, TGCT, UCS
SNORA74B_chr5_172447728_172447932	APC	11	BLCA, HNSC, KIRC, KIRP, LUAD, MESO, OV, PCPG, SKCM, UCS, UVM
SNORD127_chr14_45580085_45580171	APC	11	BLCA, BRCA, CESC, CHOL, COAD, LUAD, MESO, OV, THCA, UCEC, UCS
SNORD91B_chr17_2232310_2232531	Antimicrobials	11	BLCA, BRCA, CHOL, DLBC, HNSC, LGG, LUAD, LUSC, PAAD, PRAD, TGCT
SNORD14C_chr11_122930043_122930130	APC	11	BRCA, HNSC, KIRC, KIRP, LGG, LIHC, MESO, OV, SARC, SKCM, THYM
SNORA18_chr11_93466631_93466763	APC	11	BRCA, CESC, COAD, KICH, KIRC, KIRP, LIHC, OV, SARC, SKCM, THCA
SNORD124_chr17_38183794_38183898	Antimicrobials	11	BRCA, CHOL, DLBC, LIHC, LUAD, LUSC, MESO, OV, SKCM, THCA, UCEC
SNORD46_chr7_132437783_132437886	Antimicrobials	11	BRCA, CESC, CHOL, DLBC, HNSC, PAAD, PRAD, SKCM, TGCT, UCEC, UCS
HBII-13_chr15_25230246_25230313	Antimicrobials	11	BRCA, DLBC, ESCA, KIRP, OV, PCPG, PRAD, READ, TGCT, UCEC, UCS
SNORD82_chr2_232325078_232325153	Antimicrobials	11	BRCA, CESC, CHOL, DLBC, KIRP, LIHC, OV, PAAD, PCPG, PRAD, THYM
SNORA6_chr3_39449880_39450030	Antimicrobials	11	BRCA, CHOL, ESCA, HNSC, LIHC, LUAD, MESO, PAAD, PRAD, TGCT, THYM
SNORA68_chr19_17973396_17973529	Antimicrobials	11	BRCA, CHOL, COAD, HNSC, LUSC, PAAD, PRAD, SKCM, STAD, TGCT, UCS
SNORD104_chr17_62223437_62223517	APC	11	BRCA, CESC, HNSC, LGG, LIHC, OV, PAAD, PRAD, THCA, THYM, UCEC
U3_chr8_98370493_98370702	APC	11	BRCA, CHOL, HNSC, KICH, KIRP, LIHC, LUAD, LUSC, OV, UCEC, UVM
SNORD95_chr5_180670312_180670379	APC	11	BRCA, CESC, CHOL, HNSC, LUAD, LUSC, MESO, STAD, THCA, THYM, UVM
U49B_chr17_16342822_16342870	Antimicrobials	11	BRCA, CHOL, COAD, DLBC, KIRP, MESO, PAAD, PCPG, TGCT, THCA, UCEC
U67_chr17_7481272_7481409	APC	11	CESC, COAD, ESCA, HNSC, LIHC, LUSC, OV, PAAD, READ, SKCM, UVM
SNORA71D_chr20_37062504_37062642	APC	11	CESC, COAD, KIRC, KIRP, LGG, LUSC, OV, PRAD, SARC, SKCM, THYM
SNORA75_chr2_232320510_232320647	Antimicrobials	11	CESC, CHOL, DLBC, LIHC, LUAD, LUSC, MESO, OV, PRAD, READ, TGCT
SNORD3C_chr17_19092978_19093558	Antimicrobials	11	CESC, CHOL, COAD, DLBC, HNSC, LIHC, LUAD, READ, TGCT, THCA, UCEC
SNORD88A_chr19_51302695_51302792	Antimicrobials	11	CESC, DLBC, HNSC, KIRC, READ, SARC, STAD, TGCT, THCA, THYM, UCEC
SNORD3B-1_chr17_18965224_18965982	Antimicrobials	11	CESC, CHOL, COAD, DLBC, LIHC, LUAD, PAAD, PRAD, READ, THCA, UCEC
SNORD33_chr19_49993872_49993956	Antimicrobials	11	CESC, CHOL, DLBC, ESCA, HNSC, KIRP, LUAD, LUSC, PAAD, PCPG, UCS
SNORD51_chr2_207026602_207026681	APC	11	CESC, CHOL, HNSC, KICH, LGG, LUAD, LUSC, OV, THCA, UCEC, UVM

SnoRNA-name	Pathway	Number	Cancer types
SNORD86_chr20_2636742_2636828	APC	11	CESC, CHOL, COAD, DLBC, LUAD, OV, PCPG, SKCM, STAD, TGCT, THYM
SNORD20_chr2_232321154_232321234	Antimicrobials	11	CESC, CHOL, ESCA, LGG, LUSC, OV, PAAD, PRAD, TGCT, THCA, UCS
SNORD36B_chr9_136216948_136217023	APC	11	CESC, CHOL, COAD, DLBC, OV, PRAD, READ, SARC, SKCM, THCA, UVM
HBII-382_chr1_109643154_109643236	Antimicrobials	11	CHOL, COAD, DLBC, ESCA, HNSC, KIRP, LUAD, PRAD, READ, TGCT, THCA
SNORA52_chr11_811680_811814	APC	11	CHOL, COAD, KIRP, LIHC, LUAD, LUSC, MESO, OV, SKCM, THCA, UCEC
SNORD83B_chr22_39709823_39709916	Antimicrobials	11	CHOL, COAD, LUAD, OV, PAAD, PRAD, READ, STAD, TGCT, THYM, UCEC
SNORD46_chr1_45242162_45242265	Antimicrobials	10	ACC, BRCA, CHOL, DLBC, ESCA, HNSC, LUAD, PAAD, PRAD, TGCT
snoU2_19_chrX_20154424_20154503	APC	10	ACC, BLCA, CHOL, HNSC, KICH, LIHC, LUSC, MESO, OV, PCPG
U3_chr3_45296902_45297112	Antimicrobials	10	ACC, BLCA, CESC, CHOL, DLBC, ESCA, HNSC, LUAD, READ, UVM
HBII-382_chr1_109643154_109643236	APC	10	ACC, BLCA, CESC, LGG, LUSC, MESO, OV, SKCM, THYM, UCEC
U50_chr6_86387011_86387086	Antimicrobials	10	ACC, BLCA, CESC, COAD, ESCA, HNSC, LUAD, PRAD, STAD, UCEC
U75_chr1_173836016_173836076	Antimicrobials	10	ACC, BRCA, CHOL, DLBC, LUAD, LUSC, PCPG, PRAD, TGCT, THCA
SNORD116-14_chr15_25325287_25325381	APC	10	ACC, BLCA, BRCA, CHOL, OV, SKCM, TGCT, THCA, THYM, UCEC
SNORD34_chr19_49994161_49994231	APC	10	ACC, BLCA, BRCA, ESCA, KIRC, LGG, MESO, PCPG, STAD, THCA
SNORD85_chr1_31441009_31441084	APC	10	ACC, COAD, HNSC, KICH, LGG, LUAD, LUSC, SARC, SKCM, UVM
SNORD92_chr2_29136527_29136616	Antimicrobials	10	ACC, BRCA, DLBC, LUAD, PRAD, READ, STAD, TGCT, UCEC, UVM
U62B_chr9_134365872_134365958	Antimicrobials	10	ACC, BLCA, DLBC, HNSC, LUAD, PAAD, PCPG, PRAD, TGCT, THCA
SNORD114-15_chr14_101439006_101439078	APC	10	ACC, BLCA, KIRP, SKCM, STAD, THCA, THYM, UCEC, UCS, UVM
SNORD116-26_chr15_25344644_25344742	APC	10	ACC, BLCA, BRCA, CESC, KICH, PRAD, SKCM, THCA, UCEC, UVM
SCARNA11_chr12_6690638_6690775	APC	10	ACC, BLCA, COAD, HNSC, KIRP, LUAD, OV, READ, SKCM, THCA
SNORD115-15_chr15_25442722_25442803	APC	10	ACC, BLCA, BRCA, CESC, KICH, KIRP, LGG, LIHC, LUAD, UVM
SNORD115-43_chr15_25494344_25494426	APC	10	ACC, BRCA, CESC, CHOL, COAD, KIRP, LGG, LIHC, THCA, UCS
SNORD113-4_chr14_101402827_101402902	APC	10	ACC, KICH, LGG, LIHC, LUSC, OV, PAAD, THYM, UCS, UVM
SNORD15A_chr11_75111434_75111582	Antimicrobials	10	ACC, BRCA, CESC, DLBC, HNSC, LUAD, LUSC, STAD, UCEC, UCS
SNORD55_chr1_45241536_45241615	Antimicrobials	10	ACC, COAD, DLBC, ESCA, KIRP, LUAD, LUSC, OV, PRAD, READ
SNORD70_chr2_203142831_203142915	Antimicrobials	10	ACC, BLCA, BRCA, CHOL, HNSC, KIRP, LGG, LUSC, TGCT, THCA

SnoRNA-name	Pathway	Number	Cancer types
SNORD109B_chr15_25523489_25523556	APC	10	ACC, BLCA, COAD, LGG, MESO, OV, PCPG, SKCM, TGCT, UCS
SNORD115-6_chr15_25425643_25425725	APC	10	ACC, BRCA, CESC, CHOL, COAD, KIRP, LGG, LIHC, LUAD, THCA
SNORD116-7_chr15_25312933_25313030	APC	10	ACC, BLCA, BRCA, KIRP, LGG, MESO, THCA, THYM, UCEC, UVM
SNORD19_chr3_52725394_52725469	APC	10	ACC, BLCA, CESC, COAD, KIRC, LUAD, OV, PRAD, SARC, THCA
SNORD43_chr22_39715055_39715118	APC	10	ACC, BLCA, HNSC, KIRP, LIHC, LUAD, MESO, PRAD, SARC, SKCM
SCARNA8_chr9_19063653_19063784	APC	10	ACC, BLCA, CHOL, HNSC, KIRC, KIRP, OV, PCPG, THYM, UVM
SNORA3_chr11_8705773_8705903	Antimicrobials	10	ACC, CHOL, DLBC, ESCA, LUSC, PCPG, TGCT, THCA, THYM, UCEC
SNORD121A_chr9_33952762_33952852	Antimicrobials	10	ACC, CHOL, COAD, DLBC, HNSC, LUAD, LUSC, OV, READ, TGCT
SCARNA6_chr2_234197321_234197587	APC	10	ACC, BLCA, CESC, COAD, KICH, KIRP, LUAD, LUSC, MESO, UCS
SNORA57_chr11_62432893_62433042	APC	10	ACC, BLCA, CESC, KICH, KIRC, LUSC, OV, PRAD, SKCM, UCEC
SNORA71A_chr20_37055948_37056086	APC	10	ACC, CESC, COAD, DLBC, HNSC, KIRC, LIHC, LUSC, OV, SKCM
snoZ6_chr21_45858918_45858993	APC	10	ACC, DLBC, KICH, MESO, OV, PAAD, PCPG, SKCM, UCEC, UVM
SNORD84_chr6_31508877_31508955	APC	10	ACC, BLCA, CESC, ESCA, HNSC, LIHC, LUSC, THYM, UCEC, UVM
SNORA63_chr3_186505087_186505222	Antimicrobials	10	ACC, BRCA, CESC, CHOL, COAD, ESCA, HNSC, LUSC, PAAD, SKCM
SNORD113-9_chr14_101411985_101412057	APC	10	ACC, COAD, KICH, KIRP, LUAD, LUSC, OV, THCA, THYM, UVM
SNORA60_chr20_37078011_37078147	APC	10	ACC, BRCA, CHOL, DLBC, LIHC, PCPG, SKCM, THCA, UCEC, UVM
SNORD115-10_chr15_25432682_25432763	APC	10	ACC, BLCA, BRCA, CHOL, DLBC, KIRP, LGG, LIHC, SKCM, UVM
SNORD115-42_chr15_25492491_25492573	APC	10	ACC, BLCA, BRCA, CESC, COAD, KIRP, LGG, LIHC, OV, UVM
SNORD96A_chr5_180668814_180668892	Antimicrobials	10	ACC, BLCA, BRCA, COAD, DLBC, HNSC, PAAD, PRAD, TGCT, THCA
SNORA77_chr1_203698708_203698833	APC	10	ACC, BLCA, CESC, CHOL, COAD, LUSC, OV, PRAD, SARC, UVM
SNORD11B_chr2_203156054_203156144	Antimicrobials	10	ACC, BLCA, BRCA, CESC, HNSC, KIRP, MESO, OV, TGCT, UCEC
SNORA38_chr6_31590855_31590987	Antimicrobials	10	ACC, DLBC, ESCA, HNSC, LUAD, PAAD, PRAD, READ, UCEC, UCS
SNORD115-36_chr15_25481231_25481313	APC	10	ACC, BRCA, CESC, CHOL, KICH, KIRP, LGG, LIHC, UCS, UVM
SCARNA12_chr12_7076499_7076769	Antimicrobials	10	ACC, CESC, COAD, DLBC, ESCA, HNSC, KIRP, LUAD, PAAD, UCEC
SNORD111B_chr16_70563411_70563498	APC	10	ACC, BRCA, KIRC, LIHC, LUAD, LUSC, OV, SARC, STAD, THYM
U59B_chr12_57037463_57037538	APC	10	ACC, BLCA, DLBC, HNSC, KIRC, LIHC, LUAD, OV, PCPG, SKCM

SnoRNA-name	Pathway	Number	Cancer types
ACA47_chr17_75085388_75085575	APC	10	ACC, BLCA, BRCA, CHOL, KIRP, LUAD, LUSC, OV, UCEC, UVM
SNORA26_chr4_53579415_53579537	APC	10	ACC, BLCA, CESC, DLBC, KIRC, KIRP, LIHC, LUAD, OV, THCA
SNORD115-18_chr15_25448373_25448455	APC	10	ACC, KIRP, LGG, LIHC, READ, SKCM, THCA, UCEC, UCS, UVM
SNORA10_chr16_2012334_2012467	APC	10	ACC, CESC, COAD, DLBC, KIRP, LIHC, LUSC, OV, SKCM, THYM
U28_chr11_62622092_62622167	Antimicrobials	10	BLCA, CHOL, COAD, DLBC, ESCA, HNSC, LUAD, LUSC, PAAD, UCEC
SNORD14A_chr11_17096199_17096291	Antimicrobials	10	BLCA, ESCA, HNSC, KIRP, LUAD, PAAD, PRAD, READ, TGCT, UCS
SNORD63_chr5_137896731_137896799	APC	10	BLCA, CESC, CHOL, HNSC, LUAD, LUSC, MESO, OV, STAD, THCA
SNORA3_chr11_8705773_8705903	APC	10	BLCA, CESC, COAD, KICH, KIRC, KIRP, LUAD, MESO, OV, SKCM
SNORA51_chr20_2635712_2635844	Antimicrobials	10	BLCA, BRCA, CESC, CHOL, ESCA, LUSC, MESO, PAAD, READ, UCEC
SNORA70_chr18_3025432_3025564	APC	10	BLCA, COAD, KICH, KIRC, KIRP, LUSC, MESO, OV, SKCM, UVM
SNORD54_chr8_56986394_56986460	Antimicrobials	10	BLCA, CESC, CHOL, DLBC, LUSC, MESO, PAAD, PRAD, SKCM, TGCT
SNORD55_chr1_45241536_45241615	APC	10	BLCA, CESC, HNSC, LGG, MESO, SKCM, TGCT, UCEC, UCS, UVM
SNORD58C_chr18_47015613_47015678	APC	10	BLCA, CESC, COAD, DLBC, LGG, LUSC, OV, SARC, SKCM, THYM
SNORD97_chr11_10823013_10823155	Antimicrobials	10	BLCA, CHOL, DLBC, LUSC, MESO, PAAD, PRAD, READ, TGCT, UCEC
snoU13_chrX_23525328_23525430	Antimicrobials	10	BLCA, CESC, CHOL, COAD, DLBC, HNSC, LIHC, READ, THYM, UCEC
U3-4_chr17_19015732_19015949	Antimicrobials	10	BLCA, CHOL, COAD, DLBC, HNSC, OV, PAAD, THCA, UCEC, UCS
U49B_chr17_16342822_16342870	APC	10	BLCA, CESC, KIRC, LGG, LUAD, LUSC, OV, PRAD, SKCM, STAD
SNORA41_chr2_207026951_207027083	APC	10	BLCA, BRCA, CESC, COAD, DLBC, KIRP, LUSC, OV, SKCM, THYM
SNORD117_chr6_31504150_31504226	Antimicrobials	10	BLCA, BRCA, CHOL, DLBC, KIRP, LUAD, PAAD, SKCM, TGCT, UCEC
SNORA80B_chr2_10586839_10586975	Antimicrobials	10	BLCA, CESC, CHOL, COAD, DLBC, MESO, PAAD, PRAD, STAD, UCEC
SNORA36C_chr2_69747174_69747306	APC	10	BLCA, COAD, KIRP, MESO, OV, READ, SARC, THCA, UCEC, UCS
SNORD38A_chr1_45243513_45243584	APC	10	BLCA, COAD, DLBC, LGG, MESO, READ, SARC, SKCM, THCA, UVM
SNORD12C_chr20_47895477_47895565	APC	10	BLCA, CESC, COAD, DLBC, KIRP, PCPG, SARC, SKCM, UCS, UVM
SNORA76_chr22_34100772_34100906	APC	10	BLCA, BRCA, CHOL, COAD, DLBC, KICH, MESO, OV, SKCM, UVM
SNORD3A_chr17_19091328_19092027	APC	10	BLCA, BRCA, HNSC, KICH, KIRP, LGG, MESO, OV, SARC, UCEC
SNORA7A_chr3_12881810_12881949	APC	10	BLCA, COAD, KIRC, KIRP, LGG, MESO, OV, SARC, SKCM, THCA

SnoRNA-name	Pathway	Number	Cancer types
SNORA7B_chr3_129116052_129116191	APC	10	BLCA, CHOL, COAD, KIRC, KIRP, LGG, OV, SARC, SKCM, THCA
SNORD90_chr9_125642491_125642602	APC	10	BLCA, COAD, HNSC, KIRC, KIRP, LUAD, LUSC, SARC, SKCM, STAD
SCARNA18_chr5_82360022_82360156	APC	10	BLCA, CHOL, COAD, KIRP, LIHC, PCPG, SKCM, THYM, UCS, UVM
SNORD126_chr14_20794608_20794685	APC	10	BLCA, CESC, COAD, DLBC, ESCA, KIRC, LUAD, SKCM, UCS, UVM
SNORA24_chr15_65577799_65577929	APC	10	BLCA, COAD, DLBC, LGG, OV, PRAD, SARC, SKCM, THYM, UCS
SNORA47_chr5_76376258_76376396	APC	10	BLCA, COAD, KIRC, LUSC, MESO, SARC, SKCM, THCA, UCEC, UVM
SNORA34_chr12_49048164_49048301	Antimicrobials	10	BLCA, DLBC, HNSC, KIRP, LUAD, MESO, PAAD, PCPG, READ, SKCM
SNORA4_chr3_186505401_186505538	APC	10	BLCA, BRCA, DLBC, ESCA, KIRC, KIRP, OV, UCEC, UCS, UVM
SNORA50_chr16_58593699_58593835	APC	10	BLCA, CESC, COAD, KIRC, LUAD, LUSC, MESO, OV, SARC, THCA
SNORD121B_chr9_33934294_33934374	Antimicrobials	10	BLCA, BRCA, DLBC, KIRP, LUAD, OV, PRAD, SKCM, TGCT, THCA
SNORD10_chr17_7480128_7480276	Antimicrobials	10	BRCA, CESC, CHOL, COAD, DLBC, OV, PAAD, PRAD, TGCT, THCA
SNORD61_chrX_135961357_135961430	Antimicrobials	10	BRCA, CESC, COAD, DLBC, HNSC, KIRP, PAAD, PCPG, PRAD, SARC
SNORD9_chr14_21860309_21860412	Antimicrobials	10	BRCA, CHOL, DLBC, ESCA, HNSC, KIRP, LGG, MESO, TGCT, THCA
SNORD1B_chr17_74557189_74557275	APC	10	BRCA, CHOL, HNSC, KICH, KIRP, LIHC, LUAD, LUSC, OV, SARC
SNORA21_chr17_37009115_37009248	Antimicrobials	10	BRCA, COAD, DLBC, ESCA, LIHC, LUAD, READ, SKCM, TGCT, UCEC
SNORD97_chr11_10823013_10823155	APC	10	BRCA, CESC, COAD, KICH, LUAD, OV, PCPG, THCA, THYM, UVM
SNORD4A_chr17_27049599_27049671	Antimicrobials	10	BRCA, CHOL, COAD, DLBC, KIRP, OV, PRAD, STAD, TGCT, THCA
SNORA7B_chr3_129116052_129116191	Antimicrobials	10	BRCA, CESC, DLBC, HNSC, KICH, LIHC, LUAD, LUSC, MESO, PRAD
SNORD35B_chr19_50000975_50001063	Antimicrobials	10	BRCA, CESC, DLBC, ESCA, KIRP, LUAD, LUSC, PRAD, TGCT, UCEC
SNORD43_chr22_39715055_39715118	Antimicrobials	10	BRCA, CHOL, COAD, LUSC, PAAD, STAD, TGCT, THCA, UCEC, UCS
SNORD12_chr20_47897219_47897309	Antimicrobials	10	BRCA, CESC, CHOL, ESCA, LUSC, PRAD, READ, TGCT, THYM, UCEC
U27_chr11_62622483_62622555	Antimicrobials	10	BRCA, CESC, CHOL, KIRP, OV, PAAD, PCPG, PRAD, TGCT, UCEC
SNORD78_chr1_173834759_173834824	Antimicrobials	10	CESC, CHOL, ESCA, KIRP, LUSC, PRAD, STAD, THCA, THYM, UCEC
SNORD21_chr1_93302845_93302940	APC	10	CESC, LGG, LUAD, LUSC, MESO, SARC, SKCM, THYM, UCS, UVM
snoU2_19_chrX_20154424_20154503	Antimicrobials	10	CESC, DLBC, LUAD, PAAD, PRAD, SKCM, STAD, TGCT, THCA, UCEC
U59B_chr12_57037463_57037538	Antimicrobials	10	CESC, KICH, KIRP, LUSC, PRAD, TGCT, THCA, UCEC, UCS, UVM

SnoRNA-name	Pathway	Number	Cancer types
SNORD7_chr17_33900675_33900772	Antimicrobials	10	CESC, CHOL, HNSC, LUAD, LUSC, PRAD, TGCT, THCA, THYM, UCEC
SNORD96A_chr5_180668814_180668892	APC	10	CESC, ESCA, KIRP, LGG, LIHC, LUAD, MESO, SKCM, UCEC, UCS
SNORA45_chr11_8706985_8707116	APC	10	CESC, COAD, KICH, KIRP, MESO, OV, PCPG, SARC, UCEC, UVM
SNORD4B_chr17_27050698_27050772	APC	10	CESC, COAD, DLBC, KICH, KIRC, OV, PAAD, SKCM, UCEC, UVM
U3_chr17_56709003_56709197	APC	10	CHOL, COAD, HNSC, KICH, LGG, LUAD, LUSC, PAAD, STAD, THYM
SNORD59A_chr12_57038810_57038885	APC	10	CHOL, DLBC, HNSC, KIRC, LIHC, LUAD, LUSC, OV, SKCM, THYM
ACA43_chr9_139620555_139620691	Antimicrobials	10	CHOL, DLBC, ESCA, LIHC, LUAD, PAAD, PRAD, TGCT, UCEC, UCS
SNORD5_chr11_93466393_93466466	Antimicrobials	10	CHOL, DLBC, HNSC, LUAD, LUSC, MESO, PCPG, PRAD, TGCT, UCEC
SNORA46_chr16_58582402_58582537	Antimicrobials	10	CHOL, DLBC, HNSC, LUSC, PAAD, PCPG, PRAD, READ, STAD, THCA
SNORD37_chr19_3982504_3982570	Antimicrobials	10	CHOL, COAD, DLBC, KIRP, LUSC, PAAD, TGCT, THCA, THYM, UVM
SNORA4_chr3_186505401_186505538	Antimicrobials	10	CHOL, COAD, HNSC, LUAD, LUSC, MESO, READ, SKCM, TGCT, THYM
U22_chr11_62620381_62620507	Antimicrobials	10	COAD, DLBC, HNSC, LUAD, LUSC, PCPG, PRAD, SKCM, TGCT, THYM
SNORD91B_chr17_2232310_2232531	APC	10	COAD, ESCA, KIRC, KIRP, OV, SKCM, STAD, UCEC, UCS, UVM
SNORD23_chr19_48259109_48259219	APC	10	COAD, ESCA, KICH, KIRP, MESO, OV, READ, SKCM, UCS, UVM
SNORA80B_chr2_10586839_10586975	APC	10	ESCA, HNSC, LGG, LUAD, LUSC, OV, READ, SKCM, UCS, UVM
SNORD18A_chr15_66795581_66795652	APC	10	ESCA, KIRC, KIRP, LGG, LUAD, OV, SARC, THCA, UCEC, UVM
SNORD71_chr16_71792304_71792390	APC	10	ESCA, HNSC, KICH, LUAD, LUSC, OV, SARC, SKCM, STAD, UVM
ACA61_chr1_28906275_28906405	APC	10	KIRC, KIRP, LIHC, MESO, OV, SARC, SKCM, THCA, THYM, UVM
SNORD101_chr6_133136445_133136518	APC	9	ACC, COAD, LUSC, MESO, OV, PAAD, SKCM, THCA, UCEC
SNORD41_chr19_12817262_12817332	APC	9	ACC, CESC, DLBC, LGG, LUSC, READ, SARC, THYM, UCS
SNORD58B_chr18_47018033_47018099	Antimicrobials	9	ACC, BRCA, CESC, CHOL, ESCA, KIRP, LIHC, TGCT, THCA
SNORD116-19_chr15_25331672_25331766	APC	9	ACC, BLCA, BRCA, DLBC, LUSC, MESO, SKCM, THCA, THYM
SNORD116-17_chr15_25328733_25328827	APC	9	ACC, BLCA, CHOL, HNSC, LUSC, OV, THCA, THYM, UCEC
SNORD116-18_chr15_25330530_25330624	APC	9	ACC, BLCA, BRCA, CHOL, DLBC, LUSC, OV, THCA, UCEC
SNORD116-24_chr15_25339182_25339276	APC	9	ACC, BLCA, LUAD, LUSC, OV, SARC, SKCM, THCA, THYM
HBII-85-21_chr15_25333949_25334043	APC	9	ACC, BLCA, BRCA, DLBC, LUSC, OV, SKCM, THCA, UCEC

SnoRNA-name	Pathway	Number	Cancer types
SNORA11_chrX_54840802_54840933	APC	9	ACC, BRCA, COAD, MESO, OV, PCPG, SARC, UCS, UVM
SNORD115-12_chr15_25436562_25436644	APC	9	ACC, BLCA, BRCA, ESCA, KIRP, LGG, OV, UCEC, UVM
SNORD116-27_chr15_25346720_25346814	APC	9	ACC, BLCA, LUSC, MESO, OV, SKCM, TGCT, THCA, UCEC
HBII-13_chr15_25230246_25230313	APC	9	ACC, BLCA, CESC, HNSC, LUAD, LUSC, STAD, THCA, THYM
SNORD114-29_chr14_101456427_101456497	APC	9	ACC, CESC, COAD, LUAD, LUSC, OV, SKCM, STAD, UCS
SNORD32A_chr19_49993222_49993305	Antimicrobials	9	ACC, BLCA, BRCA, COAD, DLBC, HNSC, LUSC, MESO, SKCM
ACA17_chr9_139621198_139621331	Antimicrobials	9	ACC, CHOL, COAD, LIHC, LUAD, LUSC, PRAD, UCEC, UCS
U47_chr1_173833507_173833572	Antimicrobials	9	ACC, BRCA, CHOL, ESCA, HNSC, PAAD, PRAD, TGCT, UCS
U8_chr3_153725156_153725291	APC	9	ACC, COAD, KICH, PAAD, SKCM, STAD, UCEC, UCS, UVM
SNORD115-8_chr15_25429452_25429534	APC	9	ACC, BRCA, KICH, KIRP, LGG, LIHC, OV, SKCM, UVM
SNORD116-15_chr15_25326432_25326526	APC	9	ACC, BLCA, BRCA, LUSC, MESO, OV, THCA, THYM, UCEC
SNORD116-3_chr15_25302005_25302102	APC	9	ACC, BLCA, COAD, LGG, LUSC, OV, PAAD, THCA, THYM
SNORD72_chr5_40832757_40832837	Antimicrobials	9	ACC, BLCA, CHOL, ESCA, HNSC, LUAD, PAAD, PRAD, TGCT
SNORA13_chr5_111497181_111497314	APC	9	ACC, BLCA, CESC, KIRC, KIRP, MESO, OV, READ, SKCM
mgU2-19-30_chr11_93454679_93455032	APC	9	ACC, COAD, HNSC, LIHC, LUAD, LUSC, OV, THYM, UCS
SNORD66_chr3_184043483_184043559	APC	9	ACC, BRCA, CESC, LIHC, LUAD, OV, THCA, THYM, UVM
SNORD115-39_chr15_25486892_25486974	APC	9	ACC, BRCA, CESC, CHOL, KIRP, LGG, LIHC, MESO, UVM
SNORA34_chr12_49048164_49048301	APC	9	ACC, CESC, CHOL, COAD, LUSC, OV, PRAD, THYM, UVM
SNORD88A_chr19_51302695_51302792	APC	9	ACC, BLCA, COAD, LGG, LIHC, LUAD, PRAD, SKCM, UVM
SNORD61_chrX_135961357_135961430	APC	9	ACC, BLCA, LUAD, LUSC, OV, STAD, THCA, THYM, UCEC
SNORD115-20_chr15_25451408_25451490	APC	9	ACC, BRCA, CESC, KIRP, LGG, LIHC, MESO, SKCM, UVM
SNORD115-34_chr15_25477533_25477615	APC	9	ACC, ESCA, KIRP, LGG, LIHC, OV, SKCM, UCS, UVM
SNORA14A_chr7_75573100_75573234	APC	9	ACC, COAD, DLBC, OV, PAAD, SKCM, THCA, UCEC, UVM
SNORA49_chr12_132515768_132515905	Antimicrobials	9	ACC, BRCA, CHOL, ESCA, MESO, OV, READ, THYM, UCEC
SNORD2_chr10_58355723_58355791	APC	9	ACC, KIRC, LIHC, LUAD, MESO, OV, THCA, THYM, UCEC
SCARNA18_chr18_47340730_47340813	APC	9	ACC, BLCA, CHOL, KIRP, LGG, LUAD, MESO, THCA, UCEC

SnoRNA-name	Pathway	Number	Cancer types
SNORA73_chr18_53746625_53746825	APC	9	ACC, CHOL, KICH, OV, PRAD, SARC, SKCM, UCEC, UCS
SNORD17_chr20_17943352_17943589	APC	9	ACC, CHOL, ESCA, HNSC, LGG, LUSC, SARC, THCA, UVM
SNORA69_chr17_8232901_8233037	APC	9	ACC, CESC, COAD, KIRC, KIRP, LIHC, MESO, UCS, UVM
SNORA45_chr11_8706985_8707116	Antimicrobials	9	ACC, BLCA, CHOL, DLBC, ESCA, LUAD, PRAD, SKCM, THCA
SNORA21_chr17_37007777_37007912	APC	9	BLCA, CESC, COAD, DLBC, HNSC, KICH, LUAD, OV, SKCM
SCARNA14_chr15_66639543_66639680	Antimicrobials	9	BLCA, CHOL, COAD, DLBC, HNSC, LIHC, LUAD, UCEC, UCS
SNORA5C_chr7_45144504_45144641	Antimicrobials	9	BLCA, DLBC, HNSC, LUSC, PCPG, PRAD, TGCT, THCA, UCEC
SNORD58A_chr18_47017652_47017717	APC	9	BLCA, CESC, COAD, DLBC, ESCA, LGG, LUSC, PRAD, SKCM
SNORD18C_chr15_66793588_66793656	APC	9	BLCA, KICH, KIRC, LGG, OV, PCPG, SKCM, THCA, THYM
SNORD2_chr10_58355723_58355791	Antimicrobials	9	BLCA, CESC, CHOL, COAD, DLBC, HNSC, LGG, PRAD, UVM
SNORD88C_chr19_51305581_51305678	APC	9	BLCA, COAD, KIRC, LGG, LIHC, OV, READ, UCS, UVM
ACA61_chr1_28906275_28906405	Antimicrobials	9	BLCA, BRCA, COAD, DLBC, HNSC, PAAD, PRAD, STAD, UCS
U44_chr1_173835103_173835166	APC	9	BLCA, CESC, DLBC, HNSC, KIRP, LUAD, PRAD, SKCM, UCEC
SNORD89_chr2_101889397_101889511	Antimicrobials	9	BLCA, CHOL, DLBC, HNSC, KIRC, LUSC, PRAD, READ, UVM
SNORD60_chr16_2205023_2205106	APC	9	BLCA, DLBC, LUSC, OV, PRAD, SARC, STAD, THCA, UVM
SNORD110_chr20_2634857_2634932	Antimicrobials	9	BLCA, CHOL, COAD, DLBC, KIRP, LUAD, PRAD, THCA, UCEC
U56_chr20_2637269_2637340	APC	9	BLCA, CESC, COAD, DLBC, KIRC, LGG, LUAD, SKCM, THYM
U57_chr20_2637584_2637656	Antimicrobials	9	BLCA, CHOL, COAD, HNSC, PAAD, PRAD, TGCT, THCA, UCEC
SNORA10_chr16_2012334_2012467	Antimicrobials	9	BLCA, BRCA, CHOL, ESCA, LUAD, PAAD, PRAD, READ, UCEC
ACA45_chr15_83424696_83424823	APC	9	BLCA, COAD, KIRC, LUSC, PCPG, READ, SKCM, THCA, UVM
SNORA71A_chr20_37055948_37056086	Antimicrobials	9	BLCA, ESCA, KIRP, LUAD, PRAD, STAD, TGCT, UCEC, UCS
SNORA65_chr9_130210779_130210916	Antimicrobials	9	BLCA, BRCA, CHOL, COAD, DLBC, LUAD, LUSC, PRAD, TGCT
SNORA32_chr11_93464144_93464265	APC	9	BLCA, BRCA, KIRC, KIRP, LGG, LUSC, MESO, OV, SKCM
SNORD19B_chr3_52724759_52724843	APC	9	BLCA, COAD, HNSC, KIRP, LUSC, OV, SKCM, THCA, UVM
SNORA21_chr17_37009115_37009248	APC	9	BLCA, CESC, KICH, KIRC, KIRP, LUSC, OV, PRAD, UCS
SCARNA7_chr3_160232694_160233024	APC	9	BLCA, CESC, COAD, KIRP, LUSC, MESO, OV, PCPG, UCS

SnoRNA-name	Pathway	Number	Cancer types
SNORA31_chr1_67568328_67568462	APC	9	BLCA, KICH, KIRC, KIRP, LIHC, OV, SARC, SKCM, UCEC
SNORA70_chr21_34214171_34214305	APC	9	BLCA, CESC, COAD, KIRC, LIHC, LUSC, OV, SKCM, UVM
SNORD103B_chr1_31421961_31422052	Antimicrobials	9	BLCA, CESC, CHOL, COAD, HNSC, OV, PAAD, READ, THCA
SNORD68_chr16_89627837_89627925	APC	9	BLCA, CESC, COAD, DLBC, LGG, LIHC, LUSC, UCS, UVM
SNORD73A_chr4_152024978_152025043	APC	9	BLCA, COAD, DLBC, KIRP, LUAD, LUSC, THYM, UCEC, UVM
SNORD85_chr1_31441009_31441084	Antimicrobials	9	BLCA, CHOL, DLBC, OV, PAAD, PRAD, STAD, THCA, UCEC
U3_chr10_120545264_120545475	APC	9	BLCA, CHOL, HNSC, KIRP, LIHC, OV, THCA, UCEC, UVM
SNORD116-17_chr15_25328733_25328827	Antimicrobials	9	BRCA, CESC, COAD, DLBC, KIRC, KIRP, LGG, PRAD, SKCM
SNORD116-23_chr15_25336931_25337025	Antimicrobials	9	BRCA, DLBC, HNSC, KIRC, KIRP, LGG, PCPG, PRAD, READ
ACA45_chr15_83424696_83424823	Antimicrobials	9	BRCA, CHOL, DLBC, HNSC, KIRP, LUAD, PAAD, PRAD, UCEC
SNORA70_chr18_3025432_3025564	Antimicrobials	9	BRCA, CESC, DLBC, ESCA, LIHC, LUAD, PCPG, UCEC, UCS
ACA62_chr17_62223698_62223831	Antimicrobials	9	BRCA, ESCA, LUAD, READ, TGCT, THCA, THYM, UCEC, UCS
SNORA53_chr12_98993412_98993662	APC	9	BRCA, CHOL, DLBC, KIRP, LUSC, MESO, OV, THCA, THYM
snoU13_chrX_23525328_23525430	APC	9	BRCA, KIRC, KIRP, LGG, LUAD, PAAD, SARC, UCS, UVM
HBII-436_chr15_25227140_25227215	Antimicrobials	9	BRCA, CESC, CHOL, DLBC, ESCA, KIRP, PRAD, SKCM, TGCT
U8_chr9_38147425_38147558	APC	9	BRCA, CHOL, HNSC, LGG, LUAD, SKCM, STAD, THCA, UCEC
SNORD123_chr5_9548947_9549017	Antimicrobials	9	BRCA, CHOL, DLBC, KIRP, LIHC, LUSC, PAAD, PCPG, UVM
SNORD114-10_chr14_101433388_101433460	Antimicrobials	9	BRCA, CHOL, HNSC, LUAD, LUSC, PAAD, SKCM, TGCT, UCEC
SNORD12C_chr20_47895477_47895565	Antimicrobials	9	BRCA, HNSC, LUAD, LUSC, PAAD, PRAD, STAD, TGCT, UCEC
SNORD16_chr15_66795148_66795249	APC	9	CESC, ESCA, KIRC, LGG, LIHC, MESO, SKCM, THCA, THYM
SNORA70_chr17_26349356_26349490	Antimicrobials	9	CESC, CHOL, KIRP, LIHC, LUAD, PAAD, TGCT, THYM, UCEC
SNORA84_chr9_95054742_95054875	APC	9	CESC, COAD, DLBC, KIRP, LUSC, MESO, OV, SARC, SKCM
SNORD14A_chr11_17096199_17096291	APC	9	CESC, COAD, DLBC, KICH, LUSC, OV, SARC, SKCM, UCEC
SNORD72_chr5_40832757_40832837	APC	9	CESC, COAD, KIRC, LGG, LUSC, MESO, SARC, SKCM, UCEC
SNORD115-12_chr15_25436562_25436644	Antimicrobials	9	CESC, CHOL, COAD, DLBC, LIHC, PAAD, READ, THCA, UCS
SNORA63_chr3_183169645_183169776	APC	9	CESC, CHOL, DLBC, KIRC, MESO, OV, SKCM, UCS, UVM

SnoRNA-name	Pathway	Number	Cancer types
SNORD38B_chr1_45244061_45244130	Antimicrobials	9	CHOL, HNSC, KIRP, LIHC, LUAD, LUSC, PRAD, TGCT, THCA
HBII-276_chr8_67834708_67834784	Antimicrobials	9	CHOL, COAD, DLBC, LIHC, OV, PAAD, PRAD, THYM, UCS
SNORD6_chr11_93464668_93464740	Antimicrobials	9	CHOL, COAD, DLBC, HNSC, KIRP, PAAD, PRAD, READ, UVM
SCARNA6_chr2_234197321_234197587	Antimicrobials	9	CHOL, DLBC, KIRC, PAAD, PRAD, READ, TGCT, THCA, UCEC
SNORA55_chr1_40033045_40033182	Antimicrobials	9	CHOL, DLBC, LUSC, PAAD, PRAD, READ, SKCM, STAD, UCEC
SNORD115-38_chr15_25484984_25485066	Antimicrobials	9	CHOL, DLBC, KIRC, MESO, PAAD, PCPG, READ, THCA, UCEC
snoU2-30_chrX_20154184_20154253	APC	9	COAD, KIRP, LUAD, LUSC, MESO, OV, PCPG, THYM, UCS
SNORD2_chr3_186502584_186502654	Antimicrobials	9	COAD, DLBC, HNSC, KIRP, LUSC, MESO, PRAD, SKCM, UCEC
U25_chr11_62623036_62623103	Antimicrobials	9	DLBC, KIRP, OV, PAAD, PCPG, PRAD, READ, TGCT, UCEC
SNORA11_chrX_54953738_54953866	APC	9	DLBC, OV, PAAD, READ, SARC, SKCM, THCA, UCS, UVM
SNORD119_chr20_2443604_2443686	APC	9	DLBC, HNSC, KIRC, LGG, LIHC, PRAD, SARC, SKCM, THYM
SNORD100_chr6_133137940_133138016	APC	9	HNSC, KIRP, LIHC, LUAD, LUSC, OV, THYM, UCEC, UVM
U76_chr1_173835772_173835852	Antimicrobials	8	ACC, BRCA, CESC, ESCA, LUAD, LUSC, THCA, UCS
SNORD105B_chr19_10220432_10220511	APC	8	ACC, BLCA, KIRP, LGG, LUAD, PRAD, SKCM, THCA
SNORD24_chr9_136216250_136216325	APC	8	ACC, BLCA, CESC, COAD, LIHC, LUAD, LUSC, PRAD
SNORD52_chr6_31804852_31804919	Antimicrobials	8	ACC, COAD, LUAD, OV, PAAD, PRAD, THCA, THYM
SNORD114-9_chr14_101432365_101432437	Antimicrobials	8	ACC, CHOL, HNSC, LIHC, LUSC, PAAD, READ, UCEC
SNORD115-23_chr15_25456942_25457024	APC	8	ACC, CHOL, COAD, ESCA, KIRP, LGG, UCEC, UVM
SNORA42_chr1_155889699_155889836	Antimicrobials	8	ACC, BLCA, CHOL, ESCA, HNSC, PRAD, READ, STAD
SNORA48_chr17_7478030_7478165	APC	8	ACC, BLCA, KIRC, LGG, LIHC, LUSC, OV, SKCM
SNORD8_chr14_21865451_21865560	APC	8	ACC, CESC, MESO, PAAD, STAD, UCEC, UCS, UVM
U25_chr11_62623036_62623103	APC	8	ACC, CESC, CHOL, KIRC, LUAD, LUSC, SKCM, THCA
SNORD115-17_chr15_25446469_25446551	APC	8	ACC, BRCA, CHOL, COAD, LGG, LIHC, OV, UVM
SNORD115-19_chr15_25449503_25449585	APC	8	ACC, BRCA, CESC, KIRP, LGG, LIHC, OV, UVM
SNORD116-23_chr15_25336931_25337025	APC	8	ACC, BLCA, LUSC, OV, SKCM, TGCT, THCA, THYM
SNORA70_chr21_34214171_34214305	Antimicrobials	8	ACC, BRCA, CHOL, DLBC, ESCA, LUAD, PAAD, READ

SnoRNA-name	Pathway	Number	Cancer types
SNORA38B_chr17_65736784_65736915	APC	8	ACC, CESC, COAD, HNSC, LUSC, OV, SKCM, THCA
SNORA54_chr11_2985000_2985123	APC	8	ACC, KIRP, LUAD, MESO, OV, PCPG, SKCM, STAD
SNORD31_chr13_107973243_107973311	APC	8	ACC, BLCA, CESC, LUSC, OV, PAAD, STAD, UCEC
SNORD73A_chr4_152024978_152025043	Antimicrobials	8	ACC, BRCA, CESC, PAAD, PRAD, READ, SKCM, TGCT
SNORA47_chr5_76376258_76376396	Antimicrobials	8	ACC, CESC, CHOL, DLBC, HNSC, LUAD, OV, READ
SNORD35B_chr19_50000975_50001063	APC	8	ACC, BLCA, COAD, KIRC, PCPG, SKCM, THCA, UCS
SNORD45_chr6_38175050_38175121	Antimicrobials	8	ACC, COAD, DLBC, LUSC, PRAD, READ, SKCM, UCEC
U3_chr15_37144841_37145059	APC	8	ACC, DLBC, KICH, OV, SKCM, UCEC, UCS, UVM
U3_chr6_53012606_53012727	APC	8	ACC, CHOL, DLBC, KICH, SKCM, UCEC, UCS, UVM
HBII-52-13_chr15_25438467_25438549	APC	8	ACC, COAD, KIRP, LGG, OV, READ, UCS, UVM
snoU13_chr9_97934777_97934880	APC	8	ACC, KIRP, LGG, SKCM, THYM, UCEC, UCS, UVM
HBII-85-4_chr15_25304683_25304781	APC	8	ACC, CHOL, COAD, DLBC, HNSC, LIHC, OV, UCEC
SNORD115-35_chr15_25479393_25479475	APC	8	ACC, BLCA, COAD, ESCA, LGG, LIHC, READ, UVM
SNORA74_chr5_138611869_138612009	APC	8	ACC, CESC, COAD, KIRP, MESO, OV, SKCM, UCEC
SNORD100_chr6_133137940_133138016	Antimicrobials	8	BLCA, COAD, DLBC, PRAD, READ, SKCM, TGCT, THCA
SNORD4A_chr17_27049599_27049671	APC	8	BLCA, CESC, HNSC, LGG, LUAD, LUSC, SKCM, UVM
SNORA3_chr16_2846409_2846533	Antimicrobials	8	BLCA, CHOL, DLBC, ESCA, HNSC, LUAD, PAAD, PRAD
SNORA84_chr9_95054742_95054875	Antimicrobials	8	BLCA, CHOL, ESCA, HNSC, LUAD, PRAD, THCA, THYM
SNORD19B_chr3_52722898_52722977	APC	8	BLCA, COAD, KIRC, KIRP, LUAD, PRAD, THCA, THYM
SNORD7_chr17_33900675_33900772	APC	8	BLCA, COAD, DLBC, LGG, LIHC, MESO, SKCM, UCS
SNORA20_chr6_160201281_160201413	APC	8	BLCA, COAD, KICH, KIRP, MESO, OV, SARC, SKCM
SNORD111_chr16_70571907_70572001	Antimicrobials	8	BLCA, CHOL, KIRP, LUAD, PAAD, READ, TGCT, UVM
SNORD42A_chr17_27050447_27050510	APC	8	BLCA, CESC, COAD, MESO, OV, PRAD, SKCM, THCA
SNORD4B_chr17_27050698_27050772	Antimicrobials	8	BLCA, BRCA, KIRP, LUAD, LUSC, PRAD, TGCT, THYM
U75_chr1_173836016_173836076	APC	8	BLCA, COAD, HNSC, KIRP, LIHC, MESO, SKCM, UVM
ACA11_chr4_1976362_1976487	Antimicrobials	8	BLCA, CHOL, ESCA, LUAD, MESO, SKCM, STAD, UCEC

SnoRNA-name	Pathway	Number	Cancer types
U74_chr1_173836811_173836883	APC	8	BLCA, CESC, LUSC, PRAD, SARC, SKCM, THYM, UVM
SNORA38B_chr17_65736784_65736915	Antimicrobials	8	BLCA, CHOL, DLBC, ESCA, LUAD, MESO, READ, UCEC
SNORD12B_chr20_47896855_47896946	APC	8	BLCA, COAD, DLBC, KIRC, KIRP, SARC, SKCM, THYM
SNORD3B-1_chr17_18965224_18965982	APC	8	BLCA, KICH, KIRP, LGG, MESO, OV, SARC, SKCM
SNORA27_chr13_27829537_27829663	APC	8	BLCA, CESC, COAD, LIHC, LUSC, OV, SKCM, UCEC
SNORD20_chr2_232321154_232321234	APC	8	BLCA, COAD, DLBC, PCPG, SARC, SKCM, THYM, UCEC
SNORD50_chr12_110934157_110934226	APC	8	BLCA, COAD, KIRC, LUAD, OV, SKCM, STAD, UVM
SNORA75_chr2_232320510_232320647	APC	8	BLCA, COAD, KIRC, KIRP, LGG, SKCM, THYM, UCEC
SNORA70_chr17_26349356_26349490	APC	8	BLCA, COAD, DLBC, KIRC, LUSC, OV, PRAD, SKCM
SCARNA12_chr12_7076499_7076769	APC	8	BLCA, KICH, LUSC, MESO, OV, SKCM, TGCT, UCS
SNORA77_chr22_20113925_20114049	APC	8	BLCA, CESC, COAD, ESCA, LUSC, OV, SKCM, UCEC
hTR_chr3_169482397_169482945	APC	8	BLCA, CESC, COAD, LUSC, MESO, OV, SKCM, UVM
SNORD66_chr6_51329488_51329563	APC	8	BLCA, BRCA, COAD, LUSC, MESO, PCPG, UCEC, UVM
SNORD116-27_chr15_25346720_25346814	Antimicrobials	8	BRCA, CESC, COAD, DLBC, HNSC, KIRP, LGG, PRAD
SNORD116-3_chr15_25302005_25302102	Antimicrobials	8	BRCA, CESC, CHOL, DLBC, HNSC, KIRP, PRAD, SKCM
SCARNA5_chr2_234184371_234184649	Antimicrobials	8	BRCA, CESC, CHOL, OV, PAAD, PRAD, READ, UCS
SNORA31_chr1_67568328_67568462	Antimicrobials	8	BRCA, COAD, LUAD, MESO, PAAD, READ, STAD, UCS
SNORD113-9_chr14_101411985_101412057	Antimicrobials	8	BRCA, CHOL, DLBC, LIHC, PAAD, READ, SKCM, STAD
SNORD118_chr17_8076770_8076906	APC	8	BRCA, CESC, DLBC, KIRP, LUAD, SARC, SKCM, THCA
U50_chr6_86387011_86387086	APC	8	BRCA, CHOL, DLBC, KIRP, OV, SKCM, TGCT, THCA
HBII-135_chr17_16344539_16344612	Antimicrobials	8	BRCA, CHOL, DLBC, LIHC, MESO, PAAD, PRAD, UCEC
mgU2-19-30_chr11_93454679_93455032	Antimicrobials	8	BRCA, CESC, DLBC, KIRP, PAAD, PCPG, PRAD, READ
SNORD19B_chr3_52724759_52724843	Antimicrobials	8	BRCA, CESC, DLBC, LUAD, PRAD, TGCT, THYM, UCEC
SNORA20_chr7_39368602_39368733	Antimicrobials	8	BRCA, COAD, HNSC, LUSC, MESO, PRAD, READ, UCEC
SNORA57_chr11_62432893_62433042	Antimicrobials	8	BRCA, CHOL, COAD, DLBC, LUAD, PCPG, READ, STAD
ACA47_chr17_75085388_75085575	Antimicrobials	8	CESC, COAD, DLBC, ESCA, HNSC, READ, SKCM, THCA

SnoRNA-name	Pathway	Number	Cancer types
SNORD109B_chr15_25523489_25523556	Antimicrobials	8	CESC, CHOL, DLBC, HNSC, KIRP, PAAD, PRAD, UCEC

APC, Antigen Processing and Presentation.

Table S5. Multi-omics and clinicopathological distribution of three snoRNA-based subtypes in NSCLC.

snoRNA Cluster	Omics type	Subgroup	Count	Proportion (%)	P_value	Test_method
Cluster1	copy	1	147	37	5e-04	Fisher
Cluster1	copy	2	55	13.9	5e-04	Fisher
Cluster1	copy	3	71	17.9	5e-04	Fisher
Cluster1	copy	4	35	8.8	5e-04	Fisher
Cluster1	copy	5	53	13.4	5e-04	Fisher
Cluster1	copy	6	36	9.1	5e-04	Fisher
Cluster1	copy	Unknown	0	0	5e-04	Fisher
Cluster2	copy	1	42	11.7	5e-04	Fisher
Cluster2	copy	2	52	14.5	5e-04	Fisher

snoRNA Cluster	Omics type	Subgroup	Count	Proportion (%)	P_value	Test_method
Cluster2	copy	3	18	5	5e-04	Fisher
Cluster2	copy	4	47	13.1	5e-04	Fisher
Cluster2	copy	5	107	29.9	5e-04	Fisher
Cluster2	copy	6	89	24.9	5e-04	Fisher
Cluster2	copy	Unknown	3	0.8	5e-04	Fisher
Cluster3	copy	1	88	38.6	5e-04	Fisher
Cluster3	copy	2	26	11.4	5e-04	Fisher
Cluster3	copy	3	52	22.8	5e-04	Fisher
Cluster3	copy	4	24	10.5	5e-04	Fisher
Cluster3	copy	5	13	5.7	5e-04	Fisher
Cluster3	copy	6	22	9.6	5e-04	Fisher
Cluster3	copy	Unknown	3	1.3	5e-04	Fisher
Cluster1	Methylation	1	47	11.8	5e-04	Fisher
Cluster1	Methylation	2	69	17.4	5e-04	Fisher
Cluster1	Methylation	3	58	14.6	5e-04	Fisher
Cluster1	Methylation	4	110	27.7	5e-04	Fisher
Cluster1	Methylation	5	64	16.1	5e-04	Fisher
Cluster1	Methylation	6	46	11.6	5e-04	Fisher
Cluster1	Methylation	Unknown	3	0.8	5e-04	Fisher
Cluster2	Methylation	1	90	25.1	5e-04	Fisher
Cluster2	Methylation	2	104	29.1	5e-04	Fisher
Cluster2	Methylation	3	117	32.7	5e-04	Fisher
Cluster2	Methylation	4	30	8.4	5e-04	Fisher
Cluster2	Methylation	5	8	2.2	5e-04	Fisher
Cluster2	Methylation	6	9	2.5	5e-04	Fisher
Cluster2	Methylation	Unknown	0	0	5e-04	Fisher
Cluster3	Methylation	1	16	7	5e-04	Fisher
Cluster3	Methylation	2	44	19.3	5e-04	Fisher
Cluster3	Methylation	3	19	8.3	5e-04	Fisher
Cluster3	Methylation	4	57	25	5e-04	Fisher
Cluster3	Methylation	5	61	26.8	5e-04	Fisher
Cluster3	Methylation	6	31	13.6	5e-04	Fisher
Cluster3	Methylation	Unknown	0	0	5e-04	Fisher
Cluster1	miRNA	1	78	19.6	6.45e-57	Chi-square
Cluster1	miRNA	2	22	5.5	6.45e-57	Chi-square
Cluster1	miRNA	3	10	2.5	6.45e-57	Chi-square
Cluster1	miRNA	4	50	12.6	6.45e-57	Chi-square
Cluster1	miRNA	5	83	20.9	6.45e-57	Chi-square
Cluster1	miRNA	6	154	38.8	6.45e-57	Chi-square
Cluster2	miRNA	1	89	24.9	6.45e-57	Chi-square
Cluster2	miRNA	2	25	7	6.45e-57	Chi-square
Cluster2	miRNA	3	25	7	6.45e-57	Chi-square
Cluster2	miRNA	4	165	46.1	6.45e-57	Chi-square
Cluster2	miRNA	5	12	3.4	6.45e-57	Chi-square
Cluster2	miRNA	6	42	11.7	6.45e-57	Chi-square
Cluster3	miRNA	1	30	13.2	6.45e-57	Chi-square
Cluster3	miRNA	2	17	7.5	6.45e-57	Chi-square
Cluster3	miRNA	3	8	3.5	6.45e-57	Chi-square
Cluster3	miRNA	4	17	7.5	6.45e-57	Chi-square
Cluster3	miRNA	5	90	39.5	6.45e-57	Chi-square
Cluster3	miRNA	6	66	28.9	6.45e-57	Chi-square
Cluster1	mRNA	1	45	11.3	2.05e-63	Chi-square

snoRNA Cluster	Omics type	Subgroup	Count	Proportion (%)	P_value	Test_method
Cluster1	mRNA	2	119	30	2.05e-63	Chi-square
Cluster1	mRNA	3	87	21.9	2.05e-63	Chi-square
Cluster1	mRNA	4	58	14.6	2.05e-63	Chi-square
Cluster1	mRNA	5	52	13.1	2.05e-63	Chi-square
Cluster1	mRNA	6	36	9.1	2.05e-63	Chi-square
Cluster2	mRNA	1	11	3.1	2.05e-63	Chi-square
Cluster2	mRNA	2	34	9.5	2.05e-63	Chi-square
Cluster2	mRNA	3	16	4.5	2.05e-63	Chi-square
Cluster2	mRNA	4	64	17.9	2.05e-63	Chi-square
Cluster2	mRNA	5	101	28.2	2.05e-63	Chi-square
Cluster2	mRNA	6	132	36.9	2.05e-63	Chi-square
Cluster3	mRNA	1	0	0	2.05e-63	Chi-square
Cluster3	mRNA	2	51	22.4	2.05e-63	Chi-square
Cluster3	mRNA	3	102	44.7	2.05e-63	Chi-square
Cluster3	mRNA	4	36	15.8	2.05e-63	Chi-square
Cluster3	mRNA	5	25	11	2.05e-63	Chi-square
Cluster3	mRNA	6	14	6.1	2.05e-63	Chi-square
Cluster1	Protein	1	31	7.8	1.05e-25	Chi-square
Cluster1	Protein	2	44	11.1	1.05e-25	Chi-square
Cluster1	Protein	3	69	17.4	1.05e-25	Chi-square
Cluster1	Protein	4	85	21.4	1.05e-25	Chi-square
Cluster1	Protein	5	16	4	1.05e-25	Chi-square
Cluster1	Protein	6	11	2.8	1.05e-25	Chi-square
Cluster1	Protein	Unknown	141	35.5	1.05e-25	Chi-square
Cluster2	Protein	1	49	13.7	1.05e-25	Chi-square
Cluster2	Protein	2	96	26.8	1.05e-25	Chi-square
Cluster2	Protein	3	52	14.5	1.05e-25	Chi-square
Cluster2	Protein	4	29	8.1	1.05e-25	Chi-square
Cluster2	Protein	5	34	9.5	1.05e-25	Chi-square
Cluster2	Protein	6	3	0.8	1.05e-25	Chi-square
Cluster2	Protein	Unknown	95	26.5	1.05e-25	Chi-square
Cluster3	Protein	1	17	7.5	1.05e-25	Chi-square
Cluster3	Protein	2	5	2.2	1.05e-25	Chi-square
Cluster3	Protein	3	49	21.5	1.05e-25	Chi-square
Cluster3	Protein	4	44	19.3	1.05e-25	Chi-square
Cluster3	Protein	5	12	5.3	1.05e-25	Chi-square
Cluster3	Protein	6	24	10.5	1.05e-25	Chi-square
Cluster3	Protein	Unknown	77	33.8	1.05e-25	Chi-square
Cluster1	COCA	AD1	43	10.8	5.71e-49	Chi-square
Cluster1	COCA	AD2	34	8.6	5.71e-49	Chi-square
Cluster1	COCA	AD3	41	10.3	5.71e-49	Chi-square
Cluster1	COCA	AD4	29	7.3	5.71e-49	Chi-square
Cluster1	COCA	AD5a	9	2.3	5.71e-49	Chi-square
Cluster1	COCA	AD5b	19	4.8	5.71e-49	Chi-square
Cluster1	COCA	SQ1	142	35.8	5.71e-49	Chi-square
Cluster1	COCA	SQ2a	26	6.5	5.71e-49	Chi-square
Cluster1	COCA	SQ2b	54	13.6	5.71e-49	Chi-square
Cluster2	COCA	AD1	43	12	5.71e-49	Chi-square
Cluster2	COCA	AD2	56	15.6	5.71e-49	Chi-square
Cluster2	COCA	AD3	49	13.7	5.71e-49	Chi-square
Cluster2	COCA	AD4	85	23.7	5.71e-49	Chi-square
Cluster2	COCA	AD5a	32	8.9	5.71e-49	Chi-square

snoRNA Cluster	Omics type	Subgroup	Count	Proportion (%)	P_value	Test_method
Cluster2	COCA	AD5b	45	12.6	5.71e-49	Chi-square
Cluster2	COCA	SQ1	35	9.8	5.71e-49	Chi-square
Cluster2	COCA	SQ2a	4	1.1	5.71e-49	Chi-square
Cluster2	COCA	SQ2b	9	2.5	5.71e-49	Chi-square
Cluster3	COCA	AD1	33	14.5	5.71e-49	Chi-square
Cluster3	COCA	AD2	14	6.1	5.71e-49	Chi-square
Cluster3	COCA	AD3	15	6.6	5.71e-49	Chi-square
Cluster3	COCA	AD4	7	3.1	5.71e-49	Chi-square
Cluster3	COCA	AD5a	2	0.9	5.71e-49	Chi-square
Cluster3	COCA	AD5b	7	3.1	5.71e-49	Chi-square
Cluster3	COCA	SQ1	67	29.4	5.71e-49	Chi-square
Cluster3	COCA	SQ2a	27	11.8	5.71e-49	Chi-square
Cluster3	COCA	SQ2b	56	24.6	5.71e-49	Chi-square
Cluster1	Pathology	LUAD	147	37	9.27e-51	Chi-square
Cluster1	Pathology	LUSC	250	63	9.27e-51	Chi-square
Cluster2	Pathology	LUAD	299	83.5	9.27e-51	Chi-square
Cluster2	Pathology	LUSC	59	16.5	9.27e-51	Chi-square
Cluster3	Pathology	LUAD	64	28.1	9.27e-51	Chi-square
Cluster3	Pathology	LUSC	164	71.9	9.27e-51	Chi-square
